# Convenient Synthesis of Thiohydantoins, Imidazole-2-thiones and Imidazo[2,1-*b*]thiazol-4-iums from Polymer-Supported α-Acylamino Ketones

**DOI:** 10.3390/molecules23040976

**Published:** 2018-04-23

**Authors:** Petra Králová, Michal Maloň, Hiroyuki Koshino, Miroslav Soural

**Affiliations:** 1 Department of Organic Chemistry, Faculty of Science, Palacký University, 17. Listopadu 12, 771 46 Olomouc, Czech Republic; petra.kralova@upol.cz; 2 JEOL Ltd., Musashino 3-1-2, Akishima, Tokyo 196-8558, Japan; mmichal@jeol.co.jp; 3 RIKEN Center for Sustainable Resource Science, Hirosawa 2-1, Wako, Saitama 351-0198, Japan; koshino@riken.jp; 4 Institute of Molecular and Translational Medicine, Faculty of Medicine and Dentistry, Palacký University, Hnevotinska 5, 779 00 Olomouc, Czech Republic

**Keywords:** heterocycle, thiohydantoin, imidazole, serine, bromoketone, solid-phase synthesis

## Abstract

The preparation of 5-methylene-thiohydantoins using solid-phase synthesis is reported in this paper. After sulfonylation of immobilized Ser (*t*-Bu)-OH with 4-nitrobenzenesulfonyl chloride followed by alkylation with various bromoketones, the 4-Nos group was removed and the resulting polymer-supported α-acylamino ketones reacted with Fmoc-isothiocyanate. Cleavage of the Fmoc protecting group was followed by the spontaneous cyclative cleavage releasing the 5-methylene-thiohydantoin derivatives from the polymer support. Reduction with triethylsilane (TES) yielded the corresponding 5-methyl-thiohydantoins. When Fmoc-isothiocyanate was replaced with alkyl isothiocyanates, the trifluoroacetic acid (TFA) mediated cleavage from the polymer support, which was followed by the cyclization reaction and the imidazo[2,1-*b*]thiazol-4-iums were obtained. Their conversion in deuterated dimethylsulfoxide led to imidazole-2-thiones.

## 1. Introduction

**Scheme 1:**
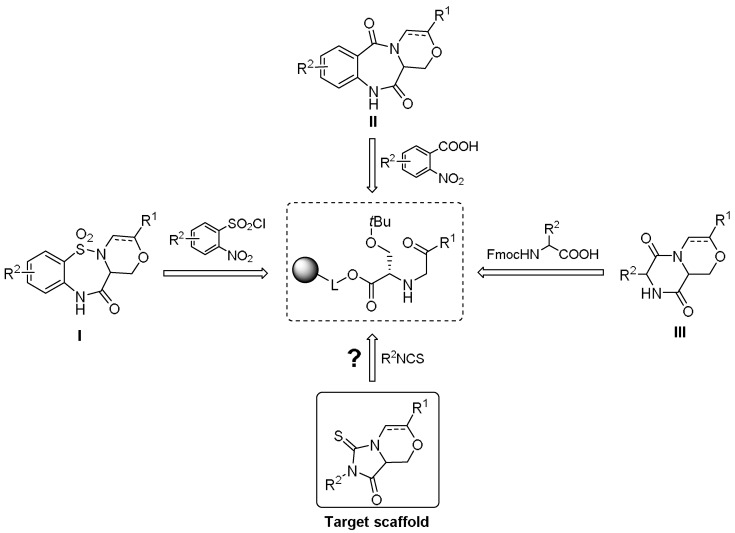
Previous applications of serine-based immobilized *α*-acylamino ketones and the proposed [5 + 6] heterocyclic scaffold.

The reaction of polymer-supported 2- or 4-nitrobenzensulfonamides with *α*-haloketones allows the simple production of many different heterocycles using the concept of diversity-oriented synthesis [1]. Recently, this chemistry was used to prepare morpholines and thiomorpholines from polymer-supported serine and cysteine via the corresponding *α*-acylamino ketones [2,3,4]. In addition, fused benzodiazepine-oxazines **I**, **II** [7 + 6] or pyrazino-oxazines **III** [6 + 6] were obtained via the acylation or sulfonylation of *α*-acylamino ketones with different reagents (see Scheme 1) [5,6,7]. As a continuation of our ongoing research in the field of solid-phase syntheses of pharmacologically promising heterocycles, we suggested the application of immobilized *α*-acylamino ketones for preparing novel fused [5 + 6] heterocycles bearing a thiohydantoin scaffold. 2-Thiohydantoins are present in the structure of many natural and synthetic compounds that can serve as antiandrogens [8], immunomodulators [9], or agricultural agents [10,11]. These derivatives also possess antimycobacterial [12], anti-inflammatory [13], anticonvulsant [14,15], antiarrhythmic [16], and anti-tumour properties [17,18]. A variety of approaches for accessing 2-thiohydantoins using both traditional solution-phase synthesis [10,19,20,21,22] and solid-phase synthesis [23,24,25,26] have been reported. In contrast, 2-thiohydantoins fused with morpholine or oxazine scaffolds and structurally related compounds have rarely been studied. Inspired by these findings, we attempted a simple synthesis to prepare the desired heterocycles from polymer-supported *α*-acylamino ketones. The actual course of reaction based on careful structural elucidation of isolated products is reported in this paper.

## 2. Results

**Scheme 2:**
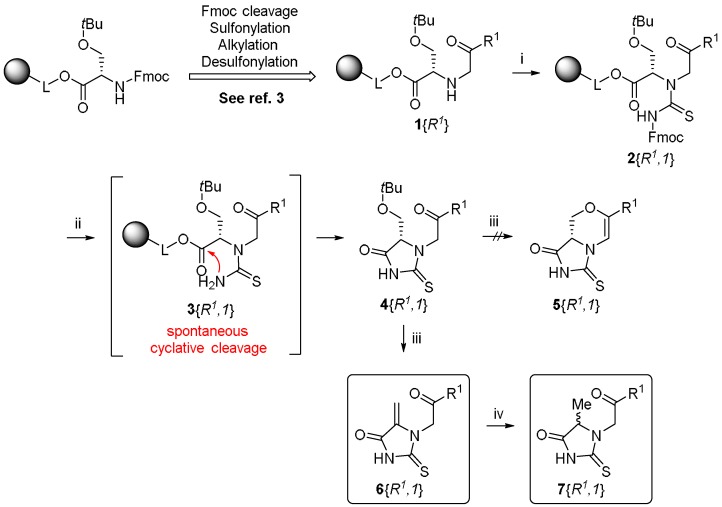
Synthesis of 5-methylidene and 5-methyl-thiohydantoins. Reagents and conditions: (i) Fmoc-NCS, anhydrous THF, 2 h, rt; (ii) 35% piperidine/DMF, 24 h, rt (or 10% piperidine/DMF for derivative **2**{*4*,*1*}); (iii) neat TFA, 20 h, 35 °C; (iv) TFA/TES/DCM (5:3:5), 24 h, rt.

The proposed synthetic pathway (see Scheme 2) was tested with 2-bromo-1-phenylethan-1-one and Fmoc-isothiocyanate as representative substrates. First, *α*-acylamino ketone **1**{*1*} (Chemset Numbering System is used hereafter) was synthetized in four steps from Wang resin-supported Fmoc-Ser(*t*-Bu)-OH based on previously reported procedures. [2] Reaction with Fmoc-NCS yielded the corresponding Fmoc-thiourea **2**{*1,1*}. After cleaving the Fmoc-protecting group, liberated thiourea **3**{*1,1*} underwent spontaneous cyclative cleavage, which released 2-thiohydantoin intermediate **3**{*1,1*} from the polymer matrix. The cleavage cocktail (DMF/piperidine) was freeze dried and the residual compound **4**{*1,1*} was subjected to TFA cyclization. However, the previously reported formation of the oxazine scaffold **5**{*1,1*} [7] was not observed and 5-methylidene-thiohydantoin **6**{*1,1*} was obtained in 80% crude purity (measured by LC-UV traces at 205–400 nm) as a result of the *t*-Bu protecting group cleavage and dehydration. We also tested TFA/TES reduction of the methylidene derivative **6**{*1,1*}, which was reported earlier to reduce dihydroxazines [2]. This provided 5-methyl-thiohydantoin **7**{*1,1*} in excellent crude purity (85%) and had an overall yield of 19%. Similar results were obtained for other 5-methyl-thiohydantoins **7**{*R*^1^,*1*}, which were obtained in crude purities of 71% to 85% and overall yields of 15% to 19% (see Table 1). 

**Scheme 3:**
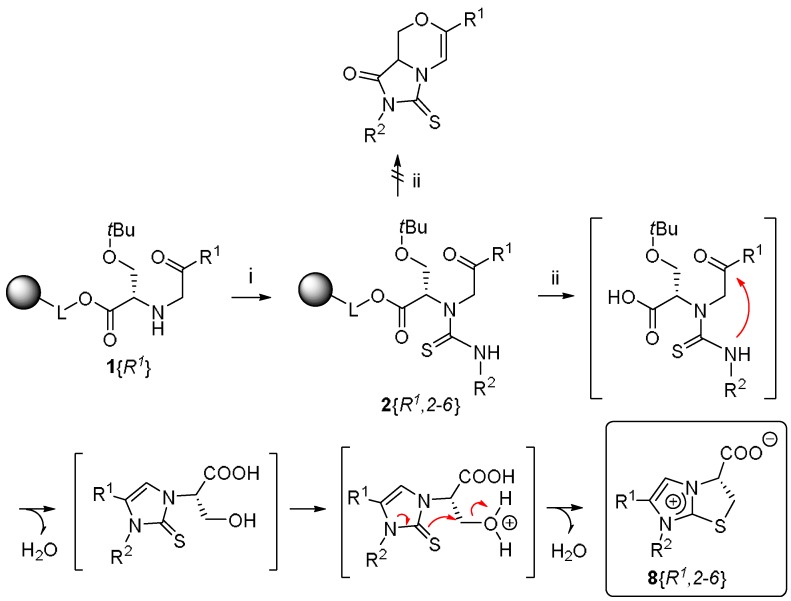
Synthesis and applicability of *N*-substituted immobilized thioureas. Reagents and conditions: (i) R^2^-NCS, anhydrous THF, 44 h, rt (or 20 h for derivatives 2{*R*^1^,*2*} and 64 h for derivatives 2{*R*^1^,*5*}); (ii) (a) neat TFA, 80 °C, 20 h (or 48 h for derivatives 2{*R*^1^,*3*}), (b) evaporation of TFA, (c) reverse-phase HPLC purification using ammonium acetate buffer, (d) freeze-drying.

To test the applicability of the reaction sequence for *N*-substituted derivatives, we subsequently replaced Fmoc-NCS with alkyl-isothiocyanates. The reaction of **1**{*1*} with benzyl-isothiocyanate yielded the corresponding *N*-benzyl-thiourea **2**{*1,2*} (see Scheme 3). In contrast to intermediates **3**{*R*^1^,*1*}, the immobilized *N*-benzyl-thiourea **2**{*1,2*} did not undergo the cyclative cleavage and trifluoroacetic acid (TFA) was used to release the compound from the resin. Further exposure to TFA under an elevated temperature led to a cyclization reaction. Although the mass spectrometry data corresponded to the expected thiohydantoin-dihydrooxazine derivative (see Scheme 3), the careful structural NMR elucidation of the isolated compound revealed that the scaffold was not formed. The cyclization to imidazole-2-thione took place followed by the dehydration and ring closure, which yielded an imidazo[2,1-*b*]thiazol-4-ium derivative **8**{*1,2*}. The product was obtained in an overall crude purity of 80% and overall yield of 53% (after semipreparative HPLC purification).

**Figure 1:**
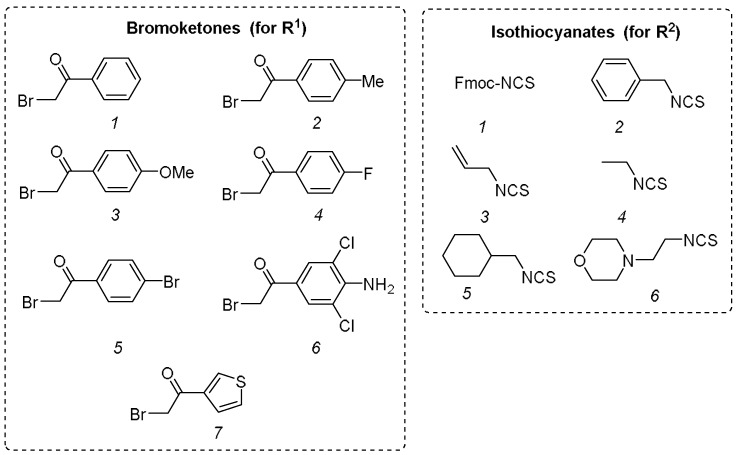
List of successfully tested building blocks.

We further tested how applicable the reaction sequence is for various α-bromoketones and isothiocyanates. Structurally diverse building blocks (see Figure 1) with electron-donating and electron-withdrawing substituents were successfully used to prepare key intermediates **2**{*R*^1^,*2*−*6*}. The reaction time was dependent on the reactivity of the used isothiocyanate. For instance, in the case of resin-bound compounds **2**{*R*^1^,*2*}, which were prepared from benzyl isothiocyanate, the formation of the corresponding thioureas was relatively fast and the reaction time was only 20 h. However, the reaction with cyclohexylmethyl isothiocyanate required noticeably longer to reach quantitative conversion (see supplementary materials for more details). The subsequent TFA-mediated cyclization was also dependent on the type of *N*-substituent, and for allyl derivatives **8**{*R*^1^,*3*}, the reaction time had to be extended from 20 h to 48 h. In total, 16 final compounds were synthesized by combining different *α*-bromoketones and isothiocyanates (see Table 1 for the list of products). The desired products were obtained and they had very good to excellent overall crude purities of 69% to 97% and good overall yields of 17% to 59%.

**Figure 2:**
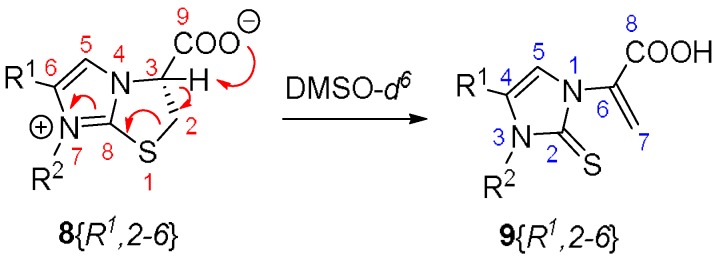
Isomerization of imidazo[2,1-b]thiazol-4-iums to imidazole-2-thiones.

When semipreparative HPLC purified compounds **8**{*R*^1^,*R*^2^} were submitted to NMR analysis in deuterated DMSO. ^1^H-NMR spectra of some derivatives showed the presence of impurity in quantities up to 18%. Compound **8**{*3,3*}, which was synthesized from allyl isothiocyanate (16% of impurity detected), was subjected to detailed NMR characterization. The data indicated ring opening may have led to the formation of **9**{*3,3*} as the corresponding impurity (see Figure 2). 

**Figure 3:**
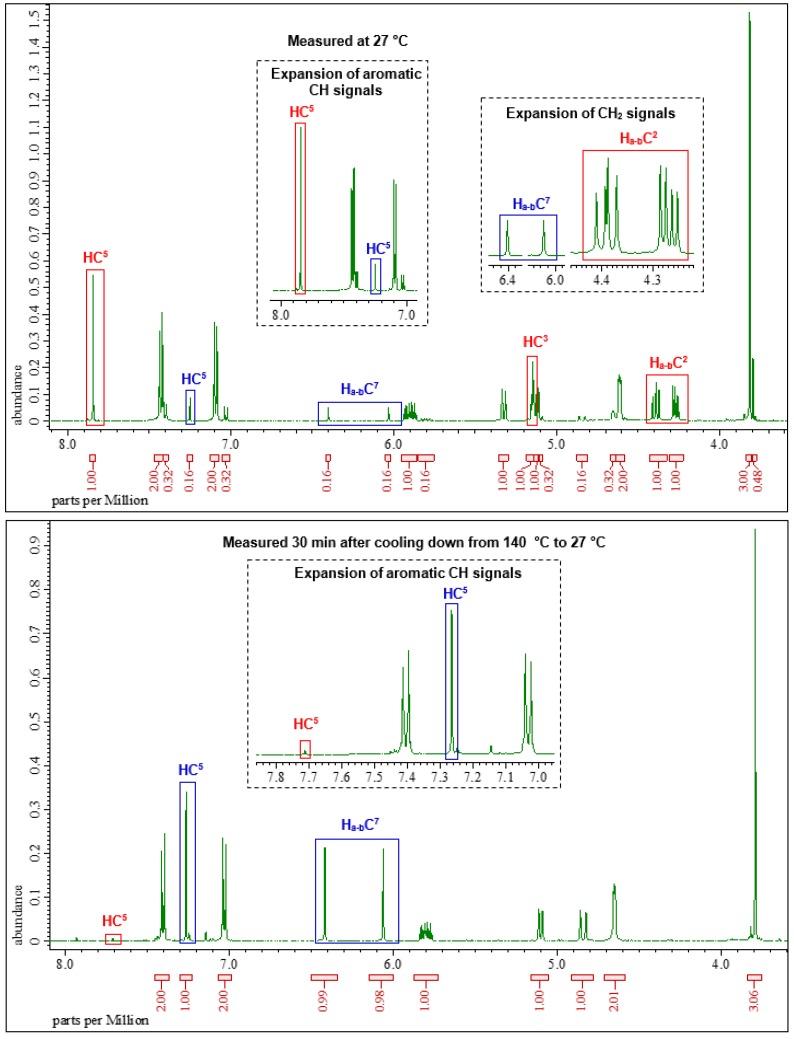
Characteristic signals of imidazo[2,1-*b*]thiazol-4-ium **8**{*3*,*3*} (highlighted in red) and imidazole-2-thione **9**{*3*,*3*} (highlighted in blue) (measured at 27 °C and after heating to 140 °C and cooling to 27 °C, respectively).

To prove the structure, **8**{*3,3*} was analyzed using gradual increase temperature ^1^H-NMR. Elevated temperatures led to a gradual increase in the content of the minor compound from 16% to 43% (80 °C) and 84% (100 °C). A nearly full conversion (97%) to the side product was observed at 120 °C. Cooling the sample to room temperature did not change the content of the new product. HPLC-MS analysis of the isolated compound confirmed its molecular weight was identical to that of the original product, which supported the putative formation of compound **9**{*3,3*} (see Figure 2). Further NMR investigations of both compounds (**8**{*3,3*} and **9**{*3,3*}) using 2D experiments confirmed their structures by identifying characteristic signals (see Figure 3). 

In the case of compound **8**{*1,2*}, which was synthesized from benzyl isothiocyanate, the slow conversion to **9**{*1,2*} was observed at 27 °C in DMSO-*d*_6_. The reaction quickly reached full conversion to **9**{*1,2*} at a higher temperature (see Supporting Information for more details). In most cases, the heating of derivatives **8**{*R*^1^,*2*–*6*} for 30 min provided compounds **9**{*R*^1^,*2*–*6*} as major products (71–100% as measured by ^1^H NMR spectra; see Table 1). If the conversion to imidazole-2-thiones **9** was not quantitative, heating for a longer time was also tested and the ratio remained unchanged, but slow decomposition of products was observed. In just one case, heating of **8**{*7,6*} did not change the initial ratio of compounds even after a prolonged time of 17 h. In the cases of **8**{*1,4*}, **8**{*3,6*}, and **8**{*4,5*}, the solubilities of the purified samples in DMSO-*d*_6_ at room temperature were limited. Therefore, short heating to 50 °C was necessary to dissolve the material for analysis. For this reason, the NMR spectra of these products are significantly enriched in the compounds **9** (40% to 92%).

## 3. Conclusions

In conclusion, we have reported a convenient method for preparing novel thiohydantoins, imidazole-2-thiones, and imidazo[2,1-*b*]thiazol-4-iums using a solid-phase synthesis technique. Depending on the type of isothiocyanate and cleavage conditions used, different products were obtained. In total, we synthetized and fully characterized more than 36 original compounds, which were generally isolated and had very good overall purities and acceptable yields. The developed strategy can be combined with previously reported protocols to synthesize different heterocycles starting from immobilized *α*-acylamino ketones by using diversity-oriented reagent-based synthesis. Derivatives **7** and **9** represent Michael acceptors and can be considered potential inhibitors of various enzymes through the irreversible Michael addition reaction, which currently represents an area of growing interest in the field of drug discovery and development [27,28,29].

## Figures and Tables

**Table 1:**
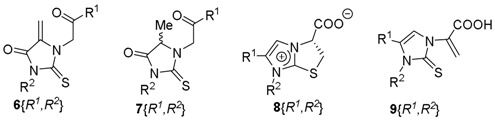
List of prepared compounds.

Cmpd	R^1^	R^2^	Crude Purity (%) ^a^	Final Purity (%) ^b^	OverallVield (%) ^c^	Ratio 8:9 At rt (%) ^c^	Ratio 8:9 after Heating (%) ^c,d^
6{*1,1*}	Ph	H	80	99	11	-	-
6{*4,1*}	4-F-Ph	H	69	98	11	-	-
6{*5,1*}	4-Br-Ph	H	75	97	7	-	-
6{*6,1*}	4-NH_2_-3,5-diClPh	H	68	98	8	-	-
6{*7,1*}	3-thiophenyl	H	85	97	8	-	-
7{*1,1*}	Ph	H	85	99	19	-	-
7{*6,1*}	4-Br-Ph	H	71	98	16	-	-
7{*7,1*}	3-thiophenyl	H	72	99	15	-	-
8{*1,2*}	Ph	Bn	80	98	53	93:7	0:100
8{*1,3*}	Ph	allyl	90	98	34	100:0	8:92
8{*1,4*}	Ph	Et	91	99	23	45:55 ^e^	11:89
8{*2,2*}	4-Me-Ph	Bn	96	99	38	97:3	3:97
8{*2,5*}	4-Me-Ph	cyclohexylmethyl	77	99	49	100:0	NT
8{*3,3*}	4-MeO-Ph	allyl	87	99	32	84:16	3:97
8{*3,4*}	4-MeO-Ph	Et	92	99	59	100:0	13:87
8{*3,6*}	4-MeO-Ph	2-(4-morpholino)ethyl	95	99	39	60:40 ^e^	0:100
8{*4,4*}	4-F-Ph	Et	85	99	57	100:0	16:84
8{*4,5*}	4-F-Ph	cyclohexylmethyl	70	99	19	8:92 ^e^	8:92
8{*5,2*}	4-Br-Ph	Bn	91	98	28	98:2	9:91
8{*5,3*}	4-Br-Ph	allyl	69	99	18	100:0	29:71
8{*5,6*}	4-Br-Ph	2-(4-morpholino)ethyl	90	98	17	88:12	0:100
8{*7,2*}	3-thiophenyl	Bn	97	98	26	97:3	0:100
8{*7,3*}	3-thiophenyl	allyl	83	98	24	89:11	0:100
8{*7,6*}	3-thiophenyl	2-(4-morpholino)ethyl	95	98	39	82:18	82:18

^a^ Overall purity after the entire reaction sequence calculated from HPLC-UV (205–400 nm); ^b^ HPLC-UV after purification (205–400 nm); ^c^ Calculated from the ^1^H-NMR spectrum of the purified product; ^d^ Sample heated at 120 °C for 30 min and measured after cooling to 27 °C; ^e^ Sample was shortly heated prior to NMR analysis to dissolve, NT—not tested.

## Supplementary Materials

The following are available online: Experimental procedures and analytical data of products.
