# Supplementary material for: Convenient Synthesis of Thiohydantoins, Imidazole-2-thiones and Imidazo[2,1-b]thiazol-4-iums from Polymer-Supported α-Acylamino Ketones
Source: Molecules. 2018 Apr 23;23(4):976. doi: 10.3390/molecules23040976 (PMC6017016; doi:10.3390/molecules23040976)
Supplement: Supplementary file 1 [file molecules-23-00976-s001.pdf]

Convenient Synthesis of Thiohydantoins, Imidazole-2-thiones and Imidazo [2,1-b]thiazol-4-iums from Polymer-supported α-Acylamino Ketones

Petra Králová[a], Michal Maloň[b], Hiroyuki Koshino[c] and Miroslav Soural[d]*

[a] Department of Organic Chemistry, Faculty of Science, Palacký University, 771 46 Olomouc, Czech Republic

[b] JEOL Ltd., Musashino 3-1-2, Akishima, Tokyo 196-8558, Japan

[c] RIKEN Center for Sustainable Resource Science, Hirosawa 2-1, Wako, Saitama 351-0198, Japan

[d] Institute of Molecular and Translational Medicine, Faculty of Medicine and Dentistry, Palacký University, Hněvotínská 5, 779 00, Olomouc, Czech Republic

*Corresponding author. E-mail: miroslav.soural@upol.cz

Content

General Information.. 2

Experimental Procedures 3

Analytical Data of Final Compounds.. 5

General Information

Solvents and chemicals were purchased from Sigma-Aldrich (Milwaukee, WI, www.sigmaaldrich.com) and Acros (Geel, Belgium, www.across.cz). The Wang resin (100-200 mesh, 1% DVB, 0.9 mmol/g) was obtained from AAPPTec (Louisville, KY, www.aapptec.com). The synthesis was carried out in plastic reaction vessels (syringes, each equipped with a porous disk) using a manually operated synthesizer (Torviq, Niles, MI, www.torviq.com). All reactions were carried out at ambient temperature (25 °C) unless stated otherwise. The volume of wash solvent was 10 mL per 1 g of resin. For washing, resin slurry was shaken with the fresh solvent for at least 1 min before changing the solvent. Resin-bound intermediates were dried by a stream of nitrogen for prolonged storage and/or quantitative analysis. For the LC/MS analysis, a sample of resin (~5 mg) was treated with TFA in DCM, the cleavage cocktail was evaporated under a stream of nitrogen, and cleaved compounds extracted into MeCN (1 mL).

The LC/MS analyses were carried out on UHPLC-MS system consisting of UHPLC chromatograph Acquity with photodiode array detector and single quadrupole mass spectrometer (Waters), using X-Select C18 column at 30 °C and flow rate of 600 *μ*L/min. The mobile phase was (A) 10 mM ammonium acetate in H_2O, and (B) MeCN, linearly programmed from 20% to 80% B over 2.5 min, kept for 1.5 min. The column was re-equilibrated with 20% of solution B for 1 min. The ESI source operated at discharge current of 5 *μ*A, vaporizer temperature of 350 °C and capillary temperature of 200 °C.

Purification was carried out on C18 reverse phase column (YMC Pack ODS-A, 20 × 100 mm, 5 *μ*m particles), the gradient was formed from 10 mM aqueous ammonium acetate and MeCN, flow rate 15 mL/min.

For lyophilization of residual solvents (H_2O, ammonium acetate buffer, DMSO, DMF) the ScanVac Coolsafe 110-4 working at -110 °C was used.

All 1D and 2D NMR experiments were performed with using ECX500 spectrometer (JEOL RESONANCE, Tokyo, Japan) at magnetic field strength of 11.75 T corresponding to ^{1}H and ^{13}C resonance frequencies of 500.16 MHz and 125.77 MHz at 27 ºC and/or higher temperature (80-140 °C). Chemical shifts (δ) are reported in parts per million (ppm) and coupling constants (*J*) are reported in Hertz (Hz). The signal of DMSO-d_6 was set at 2.50 ppm in ^{1}H NMR spectra and at 39.50

ppm in ^{13}C NMR spectra. ^{15}N chemical shifts were referenced to external 90% formamide in DMSO-d_6 (112.00 ppm) (see Martin, G. E.; Hadden, C. E., Long-Range $^{1}H-^{15}N$ Heteronuclear Shift Correlation at Natural Abundance. *J. Nat. Prod.* **2000**, *63* (4), 543-585). Acetate salt (residual agent from the semipreparative HPLC purification and/or complexed with ethylmorpholine substituents) exhibited singlet at 1.88 ppm in the ^{1}H NMR spectrum and two resonances at 21.55 ppm and 172.20 ppm in ^{13}C NMR spectrum. The assignment of ^{1}H, ^{13}C and ^{15}N signals was done by using ^{13}C DEPT, ^{1}H-^{1}H COSY, ^{1}H-^{1}H NOESY, ^{1}H-^{13}C HSQC, ^{1}H-^{13}C HMBC, ^{1}H-^{15}N HMBC, and ^{1}H-^{15}N HMQC.

Characteristic signals of imidazo[2,1-b]thiazol-4-iums **8**{R^1,*2-6*} are highlighted in red and imidazole-2-thiones **9**{R^1,*2-6*} highlighted in blue in 1D NMR spectra.

HRMS analysis was performed using LC-MS (Dionex Ultimate 3000) with Orbitrap Elite high-resolution mass spectrometer (Thermo Exactive plus, MA, USA) operating at positive full scan mode (120 000 FWMH) in the range of 100-1000 m/z. The settings for electrospray ionization were as follows: oven temperature of 150 °C and the source voltage of 3.6 kV. The acquired data were internally calibrated with diisooctylphthalate as a contaminant in MeOH (m/z 297.15909). Samples were diluted to a final concentration of 0.1 mg/mL in MeCN. Before HPLC separation (column Phenomenex Gemini, 50 × 2.00 mm, 3 μm particles, C18), the samples were injected using direct infusion into the mass spectrometer using autosampler. The mobile phase was isocratic MeCN/10 mM ammonium acetate (80:20) and flow 0.3 mL/min.

Experimental Procedures

Synthesis of α-acylamino ketones **1**{R^1} was performed starting polymer-supported Fmoc-Ser(*t*Bu)-OH according previously reported procedures (see Králová, P.; Fülöpová, V.; Maloň, M.; Volná, T.; Popa, I.; Soural, M. Stereoselective Polymer-Supported Synthesis of Morpholine- and Thiomorpholine-3-Carboxylic Acid Derivatives. *ACS Comb. Sci.* **2017**, *19* (3), 173-180).

1. Preparation of thioureas: 2{R^1,R^2}

N-unsubstituted derivatives: Fmoc-Cl (388 mg, 1.5 mmol) and KSCN (146 mg, 1.5 mmol) in anhydrous THF (5 mL) was stirred 24 h at room temperature and the solution of resulting Fmoc-isothiocyanate was filtrated using microfilter. Then the resin **1**{R^1} (500 mg), pre-washed three

times with anhydrous THF, was added to the solution. After shaking 2 h at room temperature, the resin **2**{*R*[1]*,1*} was washed three times with THF and five times with DCM.

N-alkyl derivatives: The resin **1**{*R*[1]} (500 mg) was washed three times with DCM and three times with anhydrous THF and a solution of alkyl-isothiocyanate (1.5 mmol) in anhydrous THF (5 mL) was added. After 44 h at room temperature (or 20 h for derivatives **2**{*R*[1]*,2*} and 72 h for derivatives **2**{*R*[1]*,5*}), the resin was washed three times with THF and three times with DCM.

2. Cleavage of the Fmoc-protecting group and cyclative cleavage: 3{*R*[1]*,1*}

To the resin **2**{*R*[1]*,1*} (500 mg) 5 mL of 35% piperidine in DMF (or 10% for derivative **2**{*4,1*}) was added and the resin was shaken for 24 h at room temperature. Then the resin was washed five times with 5% piperidine/DMF (5 mL) and combined washes containing product **3**{*R*[1]*,1*} were lyophilized overnight.

3. Removal of the *t*Bu-protecting group and spontaneous dehydration: 6{*R*[1]*,1*}

The crude lyophilized compounds **3**{*R*[1]*,1*} were dissolved in neat TFA (2 mL) and heated for 20 h at 35 °C. Then the reaction mixture was evaporated to dryness using a stream of nitrogen.

4. TES reduction: 7{*R*[1]*,1*}

To the crude derivatives **6**{*R*[1]*,1*} the mixture of TFA/TES/DCM (5:3:5, 3.9 mL) was added and shaken for 24 h at room temperature. The solvents and reagents were evaporated to dryness using a stream of nitrogen, the residual material was dissolved in acetonitrile and purified using semipreparative HPLC.

5. Acid-mediated cyclization: 8{*R*[1]*,2-6*}

To the resin **2**{*R*[1]*,2-6*} (500 mg) neat TFA (5 mL) was added and the mixture was heated for 20 h at 80 °C (or 48 h for derivatives **2**{*R*[1]*,3*}). The resulting solution containing products **8**{*R*[1]*,2-6*} was filtrated using a syringe with porous disk and the resin was washed five times with fresh cleaving cocktail (2 mL). Combined washes were evaporated to dryness using a stream of nitrogen, the resulting material was dissolved in acetonitrile and purified using semipreparative HPLC.

6. Conversion of imidazo[2,1-b]thiazol-4-iums to imidazole-2-thiones: 8{*R¹,2-6*} to 9{*R¹,2-6*}

Purified compounds **8**{*R¹,2-6*} were dissolved in DMSO-d_6 (600 μL) and submitted to 1D NMR spectrometry at 27 °C to collect the NMR data for **8**{*R¹,2-6*}. Then the samples were heated for 30 min at 120 °C and measured after cooling to 27 °C to collect NMR data for **9**{*R¹,2-6*}.

Analytical Data of Final Compounds

5-methylene-1-(2-oxo-2-phenylethyl)-2-thioxoimidazolidin-4-one 6{*1,1*}

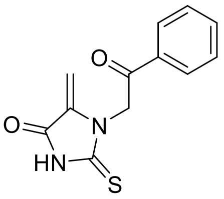

Creme amorphous solid, 7.3 mg (11%, 0.026 mmol). Cleaved from 724.8 mg of resin **2**{*1,1*} (0.475 mmol/g, 0.344 mmol of substrate). HPLC purity 98%. ^{1}H NMR (500 MHz, DMSO-d_6): δ = 12.50 (br. s, 1H), 8.06-8.09 (m, 2H), 7.71-7.75 (m, 1H), 7.58-7.62 (m, 2H), 5.59 (s, 2H), 5.35 (d, *J* = 2.9 Hz, 1H), 5.32 (d, *J* = 2.9 Hz, 1H). ^{13}C NMR (126 MHz, DMSO-d_6): δ = 191.41, 179.15, 163.40, 136.93, 134.24, 134.09, 128.89, 128.11, 98.20, 49.39. HRMS (ESI-TOF, pos.): *m/z* calcd for $C_{12}H_{11}N_2O_2S$ $[M+H]^+$ 247.0536, found 247.0535.

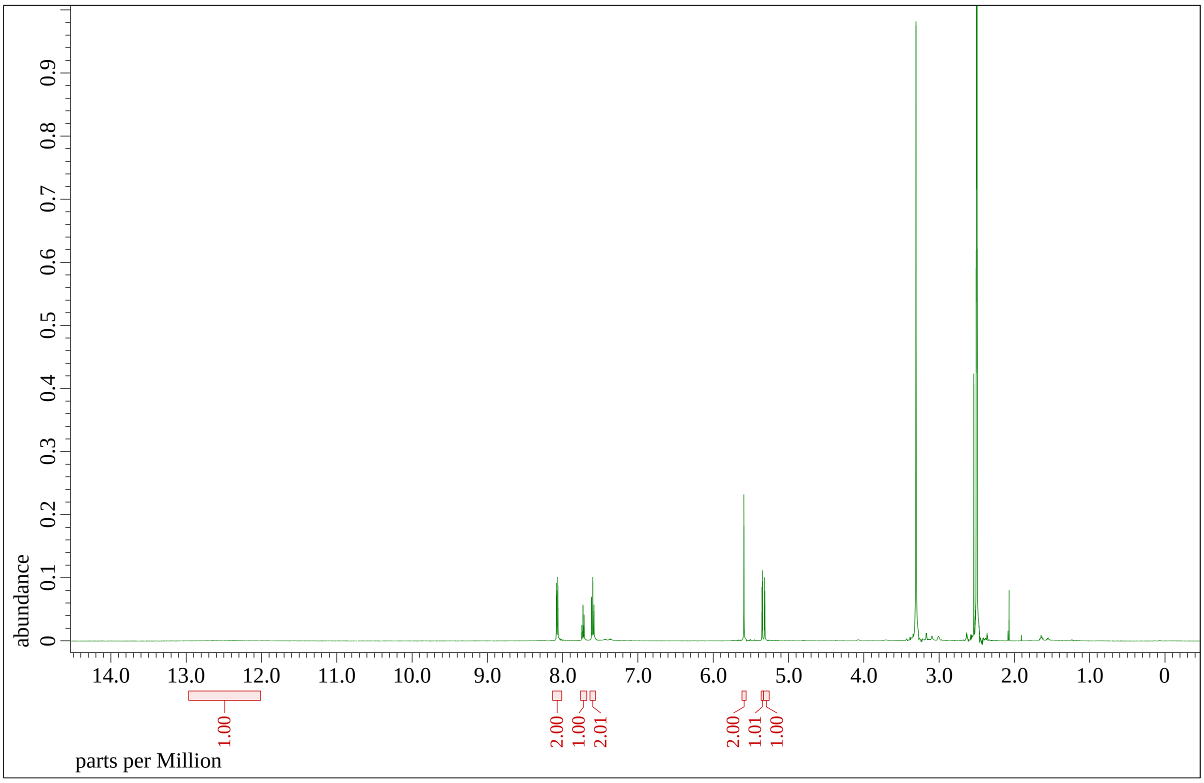

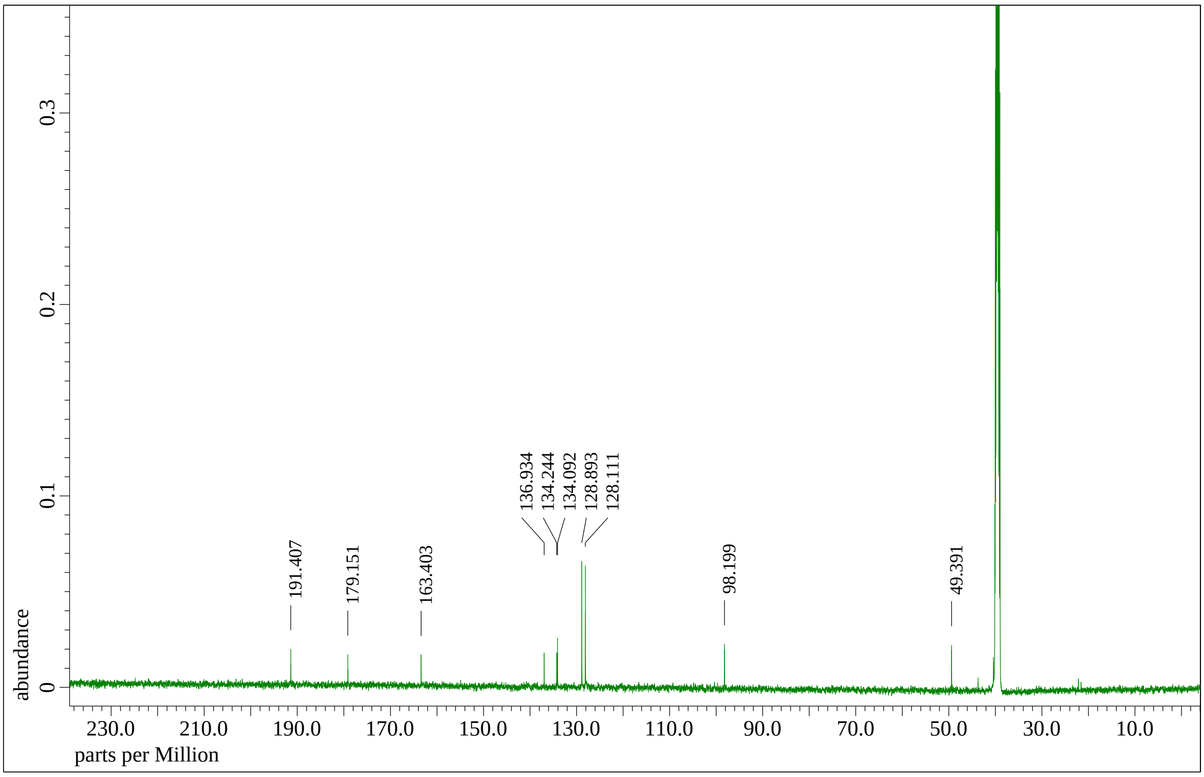

1-(2-(4-fluorophenyl)-2-oxoethyl)-5-methylene-2-thioxoimidazolidin-4-one 6{*4,1*}

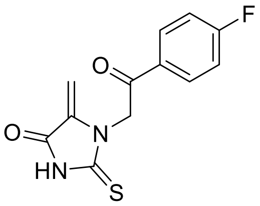

Creme amorphous solid, 6.9 mg (11%, 0.026 mmol). Cleaved from 505.7 mg of resin **2**{*4,1*} (0.460 mmol/g, 0.233 mmol of substrate). HPLC purity 98%. ^{1}H NMR (500 MHz, DMSO-d_6): δ = 12.61 (br. s, 1H), 8.07 (br. dd, J = 8.9, 5.5 Hz, 2H), 7.42 (br. dd, J = 8.9, 8.9 Hz, 2H), 5.58 (s, 2H), 5.35 (d, J = 2.8 Hz, 1H), 5.31 (d, J = 2.8 Hz, 1H). ^{13}C NMR (126 MHz, DMSO-d_6): δ = 190.10, 179.15, 165.49 (d, J = 253.1 Hz), 163.38, 136.91, 131.26 (d, J = 9.6 Hz), 131.02 (d, J = 2.4 Hz), 115.99 (d, J = 22.8 Hz), 98.20, 49.33. HRMS (ESI-TOF, pos.): *m/z* calcd for $C_{12}H_{10}FN_2O_2S$ [M+H]$^+$ 265.0442, found 265.0439.

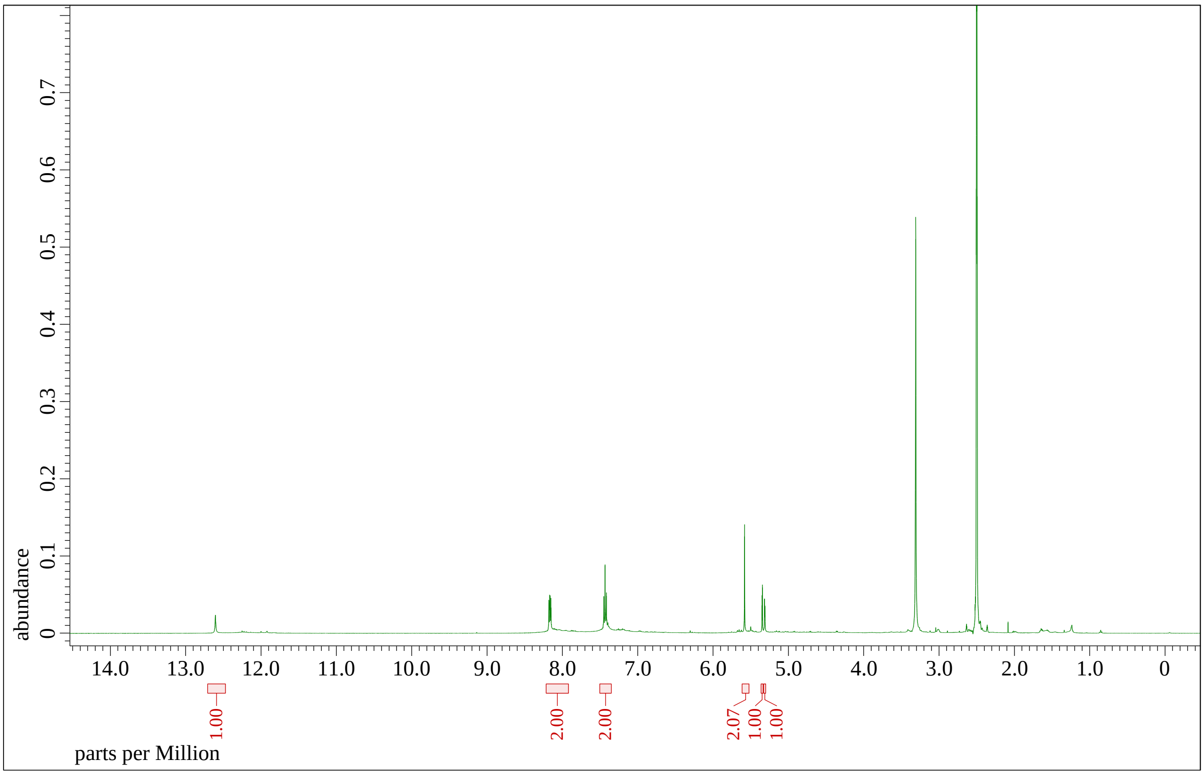

abundance
parts per Million
1.00
2.00
2.00
2.07
1.00
1.00

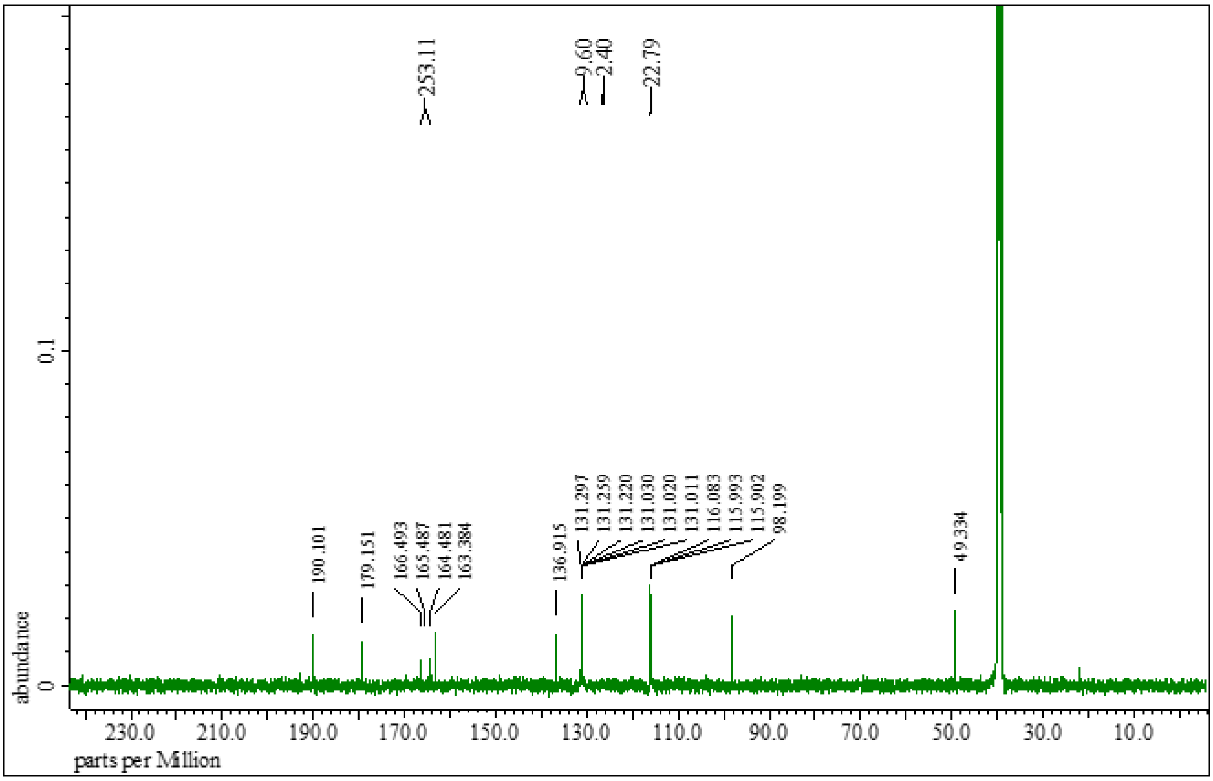

abundance
parts per Million
190.101
179.151
166.493
165.487
164.481
163.384
136.915
131.297
131.259
131.220
131.030
131.020
131.011
116.083
115.993
115.902
98.199
49.334

1-(2-(4-bromophenyl)-2-oxoethyl)-5-methylene-2-thioxoimidazolidin-4-one 6{*5,1*}

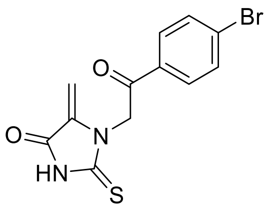

Creme amorphous solid, 6.0 mg (7%, 0.019 mmol). Cleaved from 546.6 mg of resin **2**{*5,1*} (0.503 mmol/g, 0.275 mmol of substrate). HPLC purity 97%. ^{1}H NMR (500 MHz, DMSO-d_6): δ = 12.61 (br. s, 1H), 8.01 (br. d, J = 8.6 Hz, 2H), 7.82 (br. d, J = 8.6 Hz, 2H), 5.57 (s, 2H), 5.35 (d, J = 2.8 Hz, 1H), 5.30 (d, J = 2.8 Hz, 1H). ^{13}C NMR (126 MHz, DMSO-d_6): δ = 190.85, 179.12, 163.36, 136.89, 133.23, 131.98, 130.11, 128.25, 98.24, 49.37. HRMS (ESI-TOF, neg.): m/z calcd for $C_{12}H_8BrN_2O_2S$ $[M-H]^-$ 322.9484, found 322.9483.

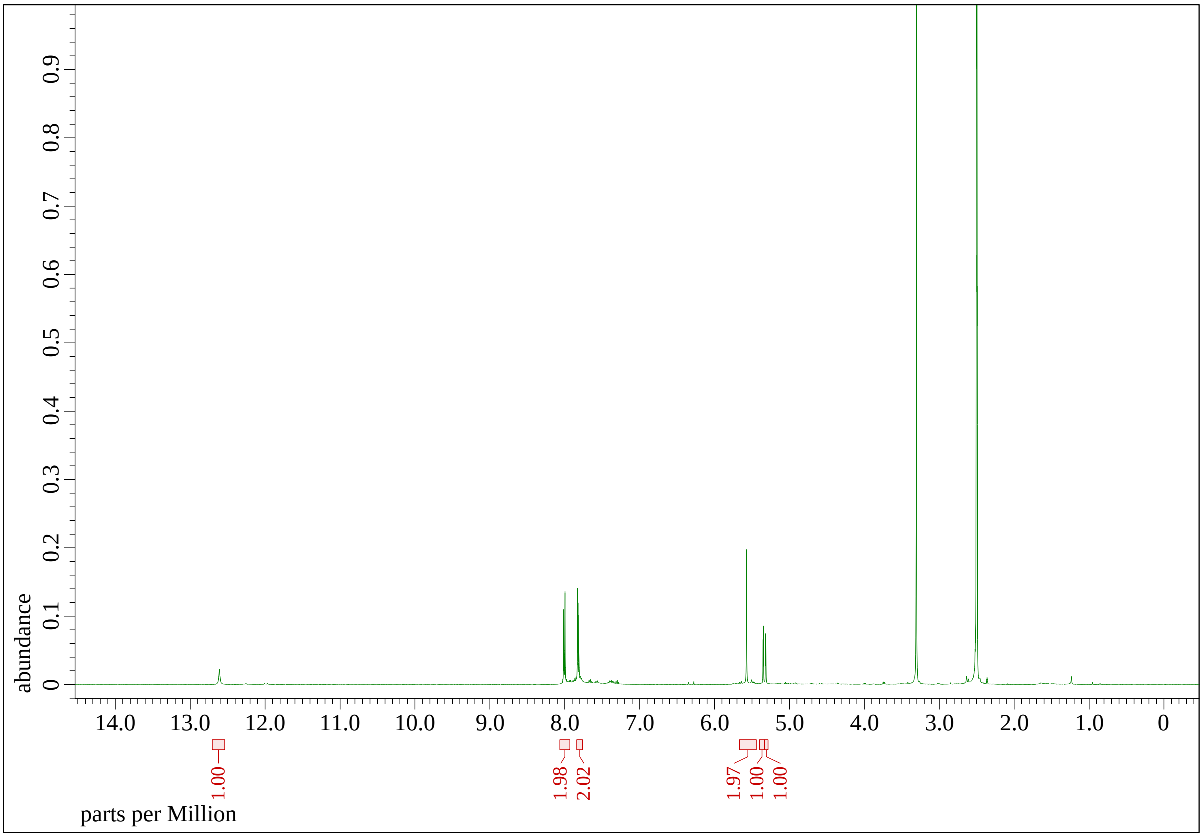

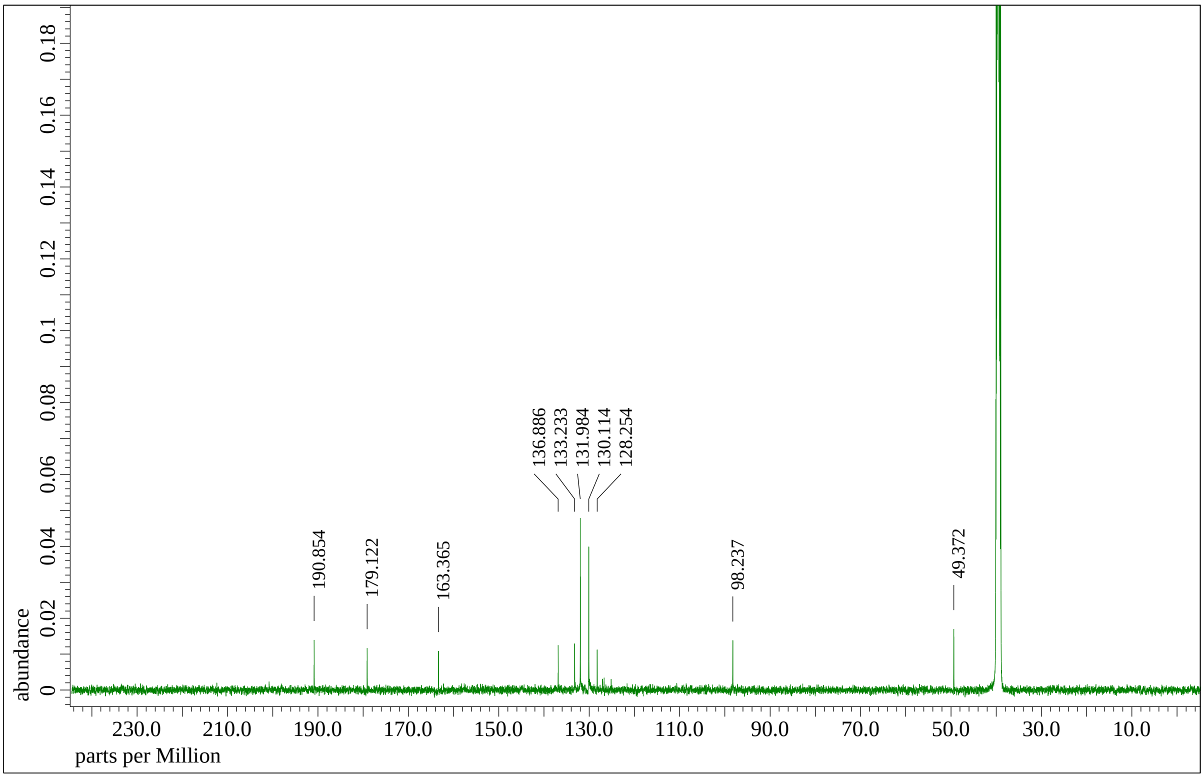

1-(2-(4-amino-3,5-dichlorophenyl)-2-oxoethyl)-5-methylene-2-thioxoimidazolidin-4-one 6{*6,1*}

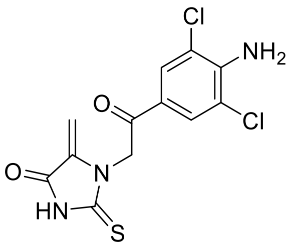

Creme amorphous solid, 7.8 mg (8%, 0.024 mmol). Cleaved from 490.8 mg of resin **2**{*6,1*} (0.460 mmol/g, 0.226 mmol of substrate). HPLC purity 98%. ^{1}H NMR (500 MHz, DMSO-d_6): δ = 12.52 (br. s, 1H), 7.94 (s, 2H), 6.60 (br. s, 2H), 5.47 (s, 2H), 5.31 (d, J = 2.8 Hz, 1H), 5.24 (d, J = 2.8 Hz, 1H). ^{13}C NMR (126 MHz, DMSO-d_6): δ = 187.54, 179.15, 163.39, 146.19, 136.94, 128.52, 122.60, 117.33, 98.04, 48.79. HRMS (ESI-TOF, pos.): *m/z* calcd for $C_{12}H_{10}Cl_2N_3O_2S$ $[M+H]^+$ 329.9865, found 329.9863.

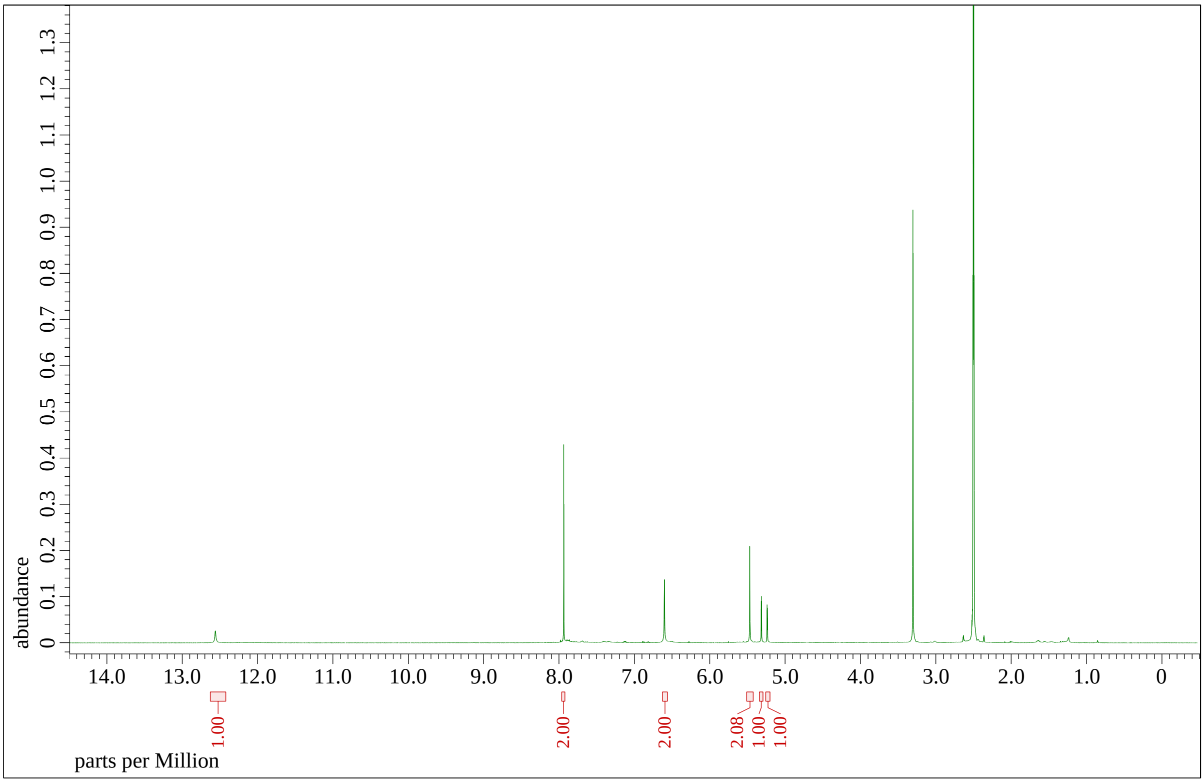
abundance
0
0.1
0.2
0.3
0.4
0.5
0.6
0.7
0.8
0.9
1.0
1.1
1.2
1.3
14.0
13.0
12.0
11.0
10.0
9.0
8.0
7.0
6.0
5.0
4.0
3.0
2.0
1.0
0
1.00
2.00
2.00
2.08
1.00
1.00
parts per Million

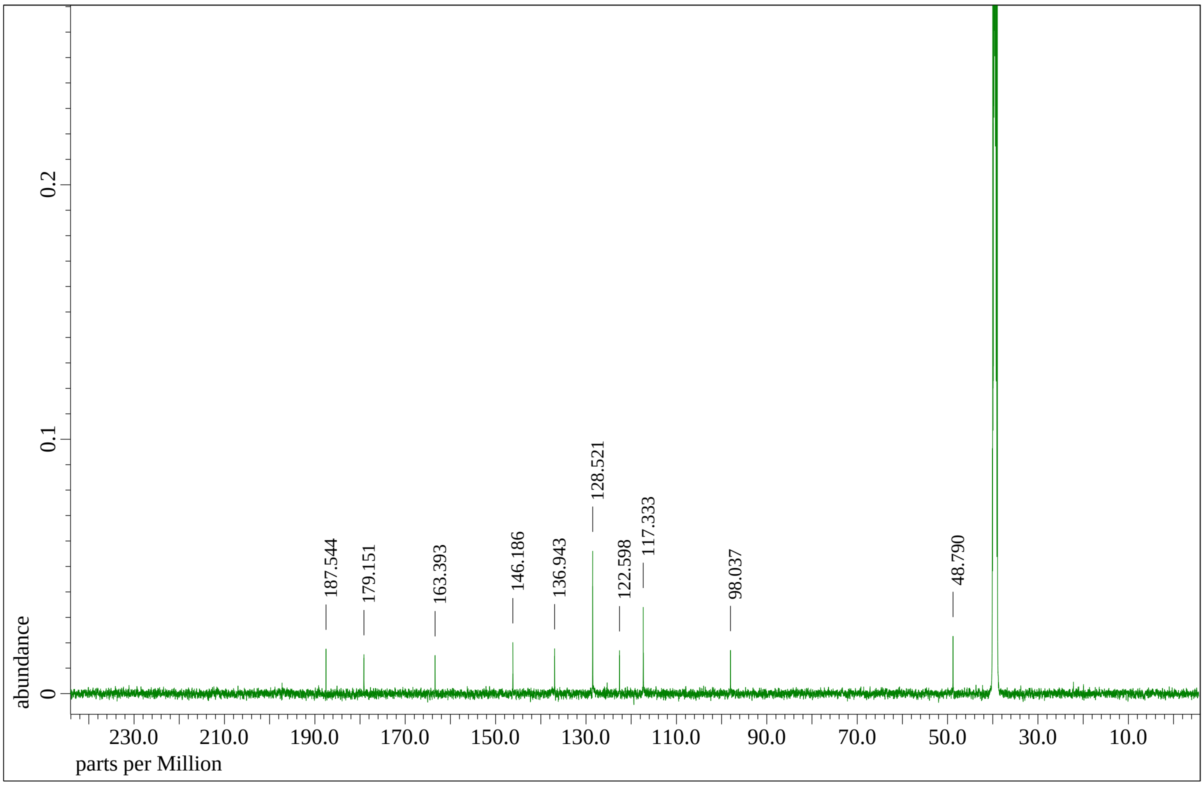
abundance
0
0.1
0.2
187.544
179.151
163.393
146.186
136.943
128.521
122.598
117.333
98.037
48.790
230.0
210.0
190.0
170.0
150.0
130.0
110.0
90.0
70.0
50.0
30.0
10.0
parts per Million

5-methylene-1-(2-oxo-2-(thiophen-3-yl)ethyl)-2-thioxoimidazolidin-4-one 6{*7,1*}

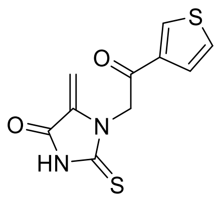

Creme amorphous solid, 7.9 mg (8%, 0.032 mmol). Cleaved from 749.1 mg of resin **2**{*7,1*} (0.503 mmol/g, 0.377 mmol of substrate). HPLC purity 97%. ^{1}H NMR (500 MHz, DMSO-d_6): δ = 12.58 (br. s, 1H), 8.74 (dd, J = 2.8, 1.3 Hz, 1H), 7.71 (dd, J = 5.1, 2.8 Hz, 1H), 7.58 (dd, J = 5.1, 1.3 Hz, 1H), 5.48 (s, 2H), 5.34 (d, J = 2.8 Hz, 1H), 5.29 (d, J = 2.8 Hz, 1H). ^{13}C NMR (126 MHz, DMSO-d_6): δ = 185.87, 179.13, 163.37, 138.84, 136.93, 134.71, 127.85, 126.36, 98.12, 49.63. HRMS (ESI-TOF, neg.): *m/z* calcd for $C_{10}H_7N_2O_2S_2$ $[M-H]^-$ 250.9943, found 250.9951.

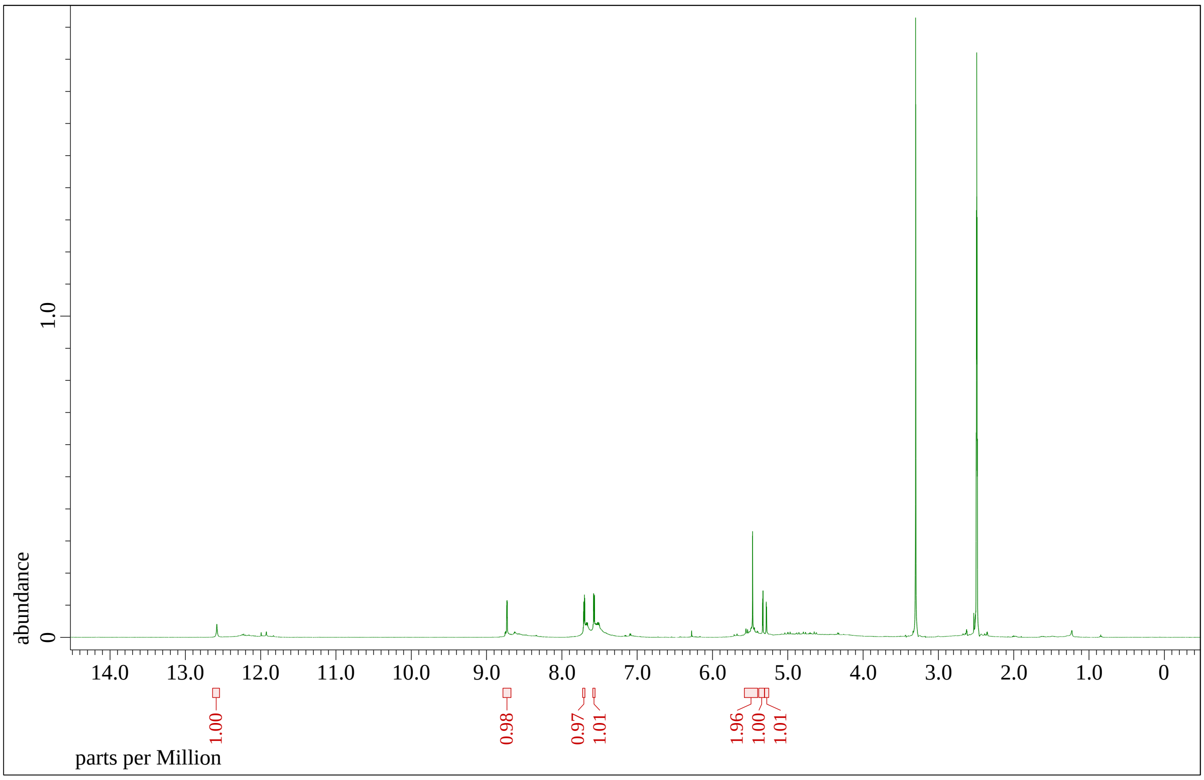

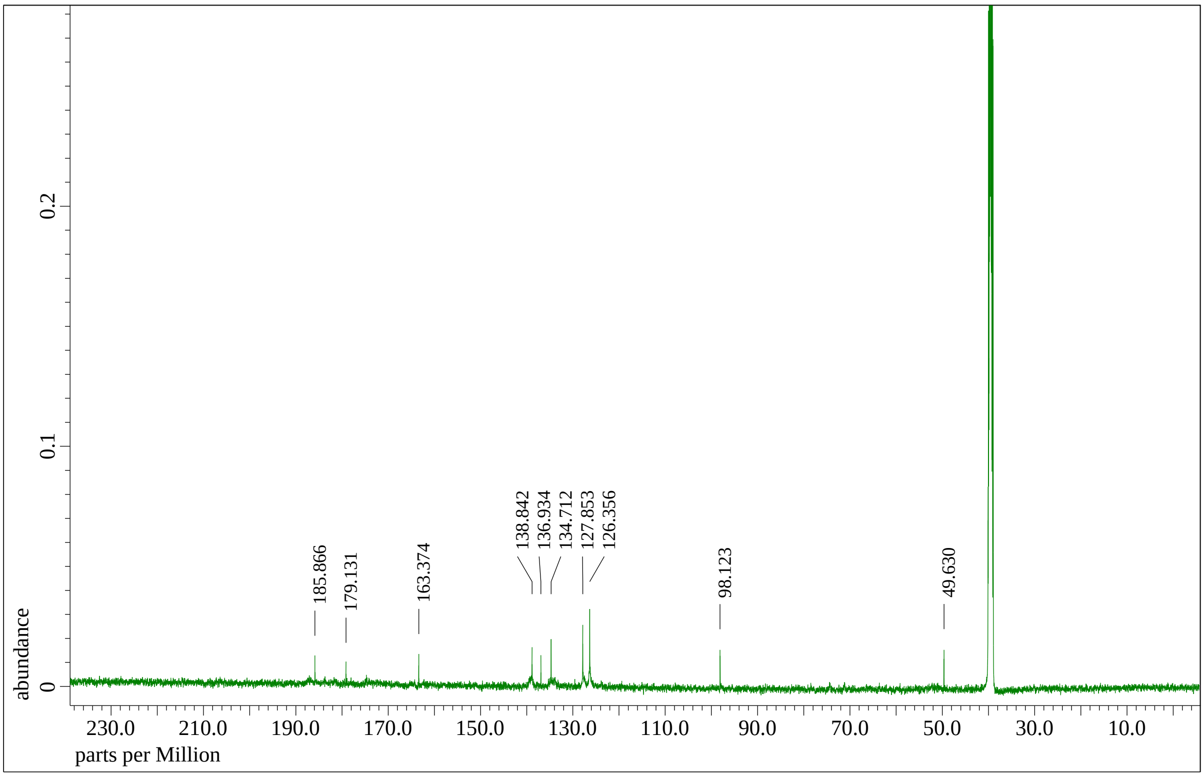

(5*S*)-5-methyl-1-(2-oxo-2-phenylethyl)-2-thioxoimidazolidin-4-one 7{*1,1*}

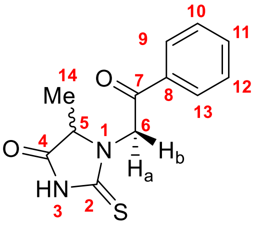

White amorphous solid, 23.0 mg (19%, 0.093 mmol). Cleaved from 1 029.0 mg of resin **2**{*1,1*} (0.475 mmol/g, 0.489 mmol of substrate). HPLC purity 99%. ^{1}H NMR (500 MHz, DMSO-d_6): δ = 12.06 (br. s, 1H, HN^3), 8.02-8.05 (m, 2H, $HC^{9,13}$), 7.69-7.73 (m, 1H, HC^{11}), 7.56-7.60 (m, 2H, $HC^{10,12}$), 5.42 (d, J = 18.2 Hz, 1H, H_aC^6), 5.14 (d, J = 18.2 Hz, 1H, H_bC^6), 4.31 (q, J = 7.1 Hz, 1H, HC^5), 1.35 (d, J = 7.1 Hz, 3H, HC^{14}). ^{13}C NMR (126 MHz, DMSO-d_6): δ = 192.90 (C7), 182.43 (C2), 175.66 (C4), 134.50 (C8), 134.02 (C11), 128.95 (C10,12), 128.02 (C9,13), 60.01 (C5), 50.36 (C6), 14.54 (C14). ^{15}N NMR (51 MHz, DMSO-d$_6$): δ = 167.0 (N3), 130.6 (N1). HRMS (ESI-TOF, pos.): *m/z* calcd for $C_{12}H_{13}N_2O_2S$ $[M+H]^+$ 249.0692, found 249.0692.

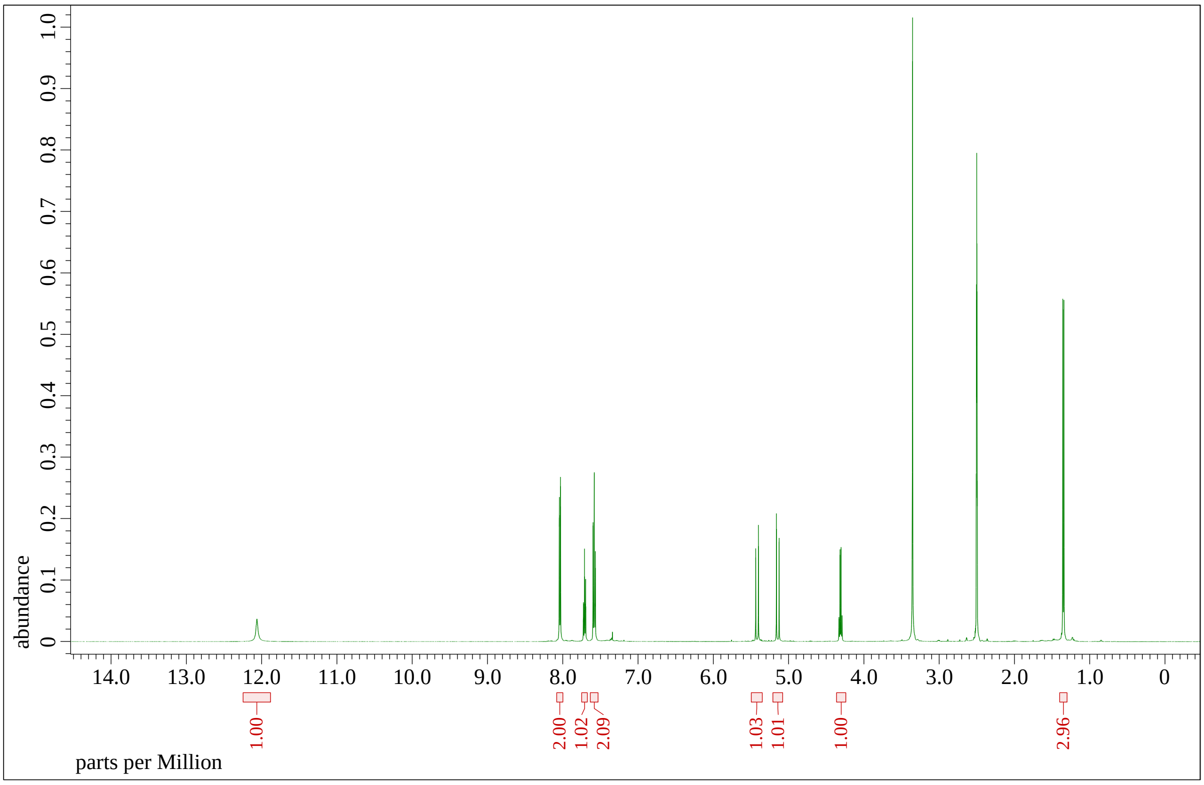

abundance
parts per Million
14.0
13.0
12.0
11.0
10.0
9.0
8.0
7.0
6.0
5.0
4.0
3.0
2.0
1.0
0
1.00
2.00
1.02
2.09
1.03
1.01
1.00
2.96

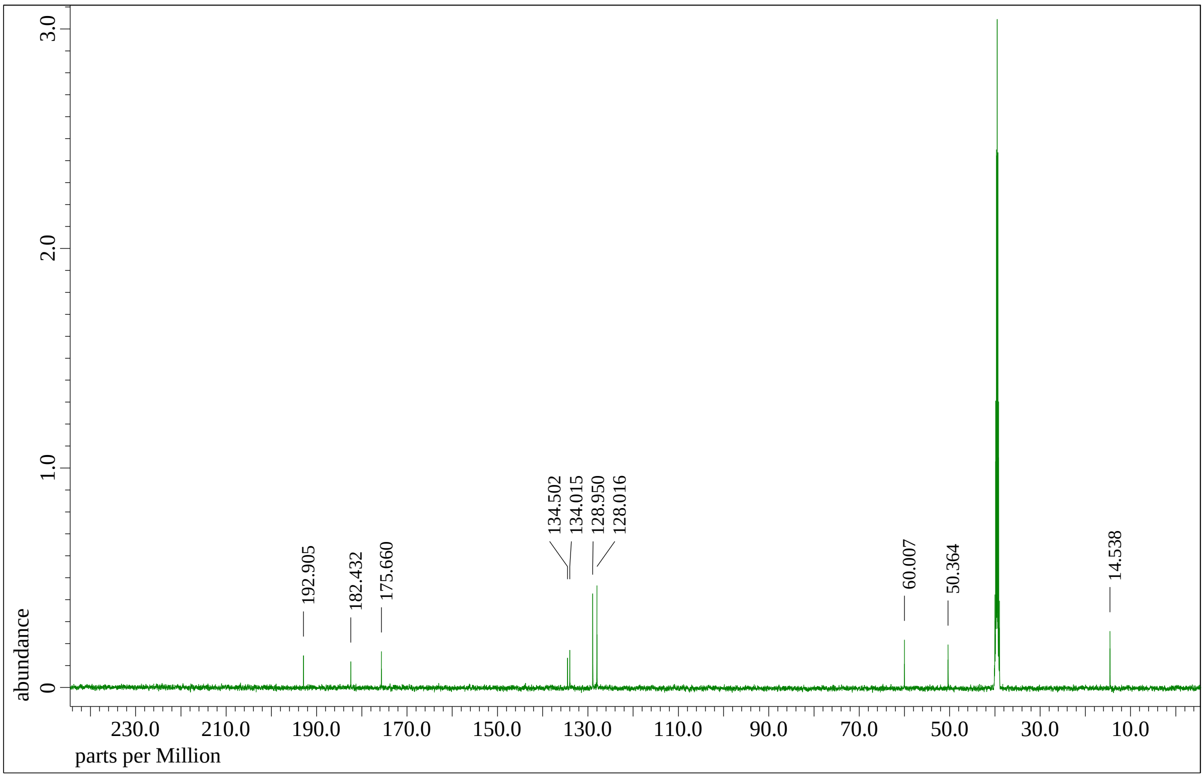

abundance
parts per Million
230.0
210.0
190.0
170.0
150.0
130.0
110.0
90.0
70.0
50.0
30.0
10.0
192.905
182.432
175.660
134.502
134.015
128.950
128.016
60.007
50.364
14.538

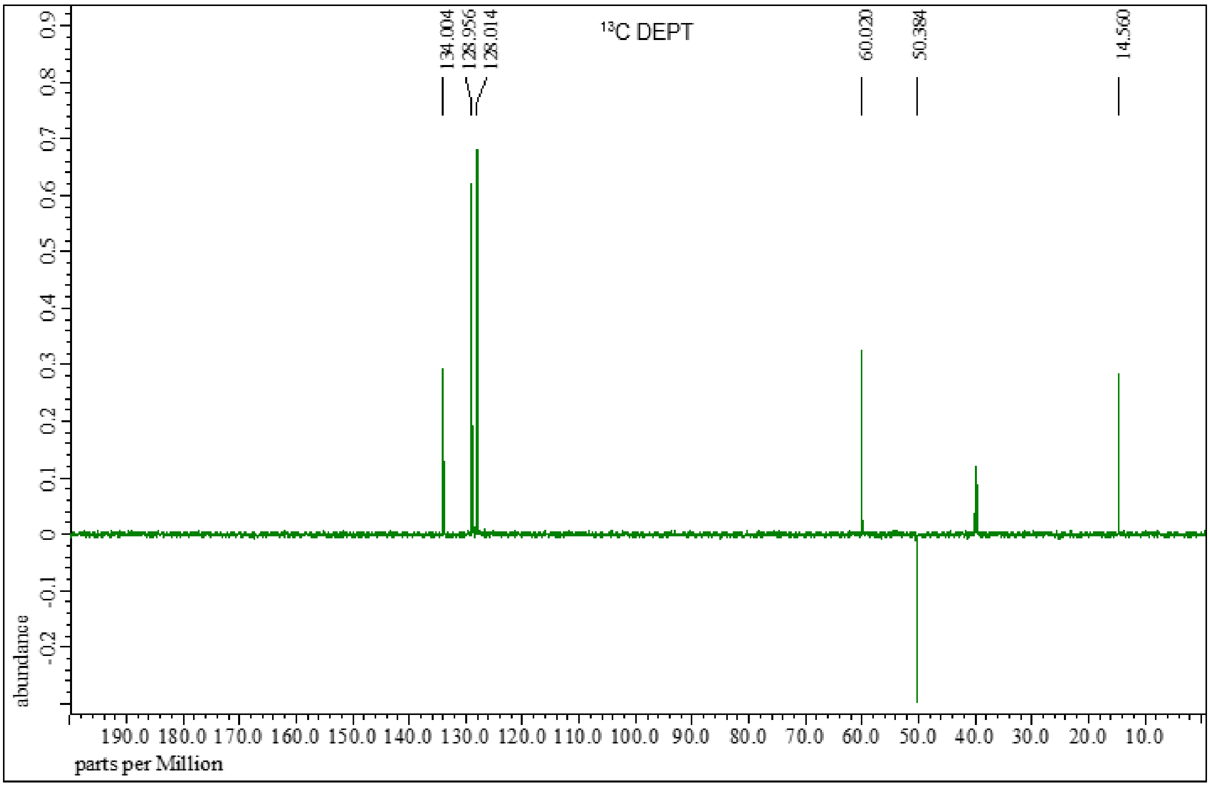

134.004
128.956
128.014
^{13}C DEPT
60.020
50.384
14.560
abundance
parts per Million

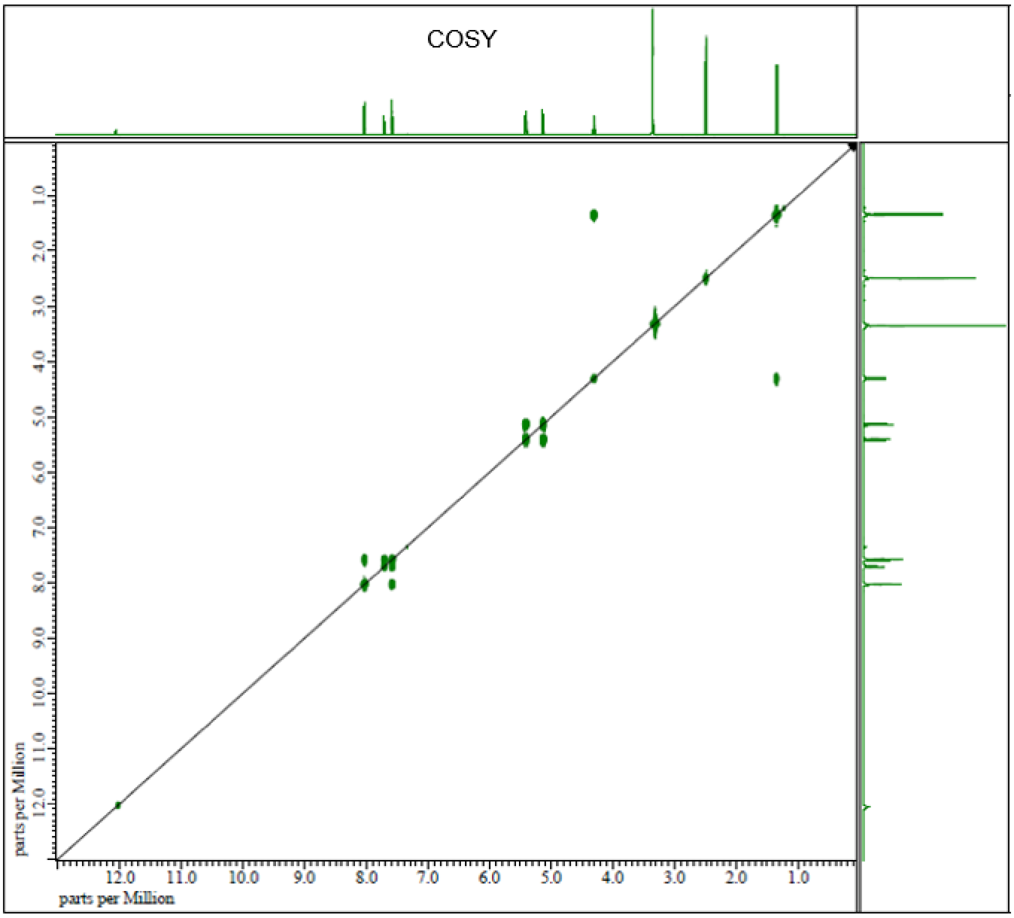

COSY
parts per Million
parts per Million

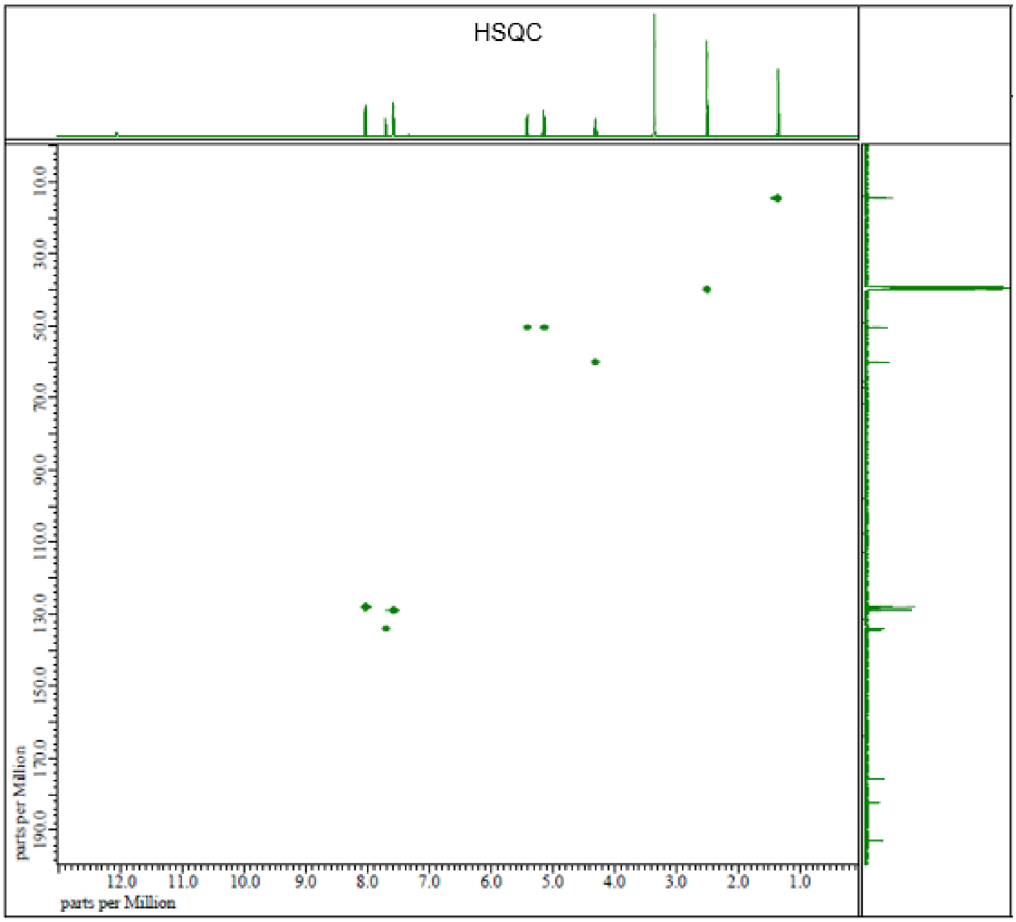
HSQC
parts per Million
parts per Million

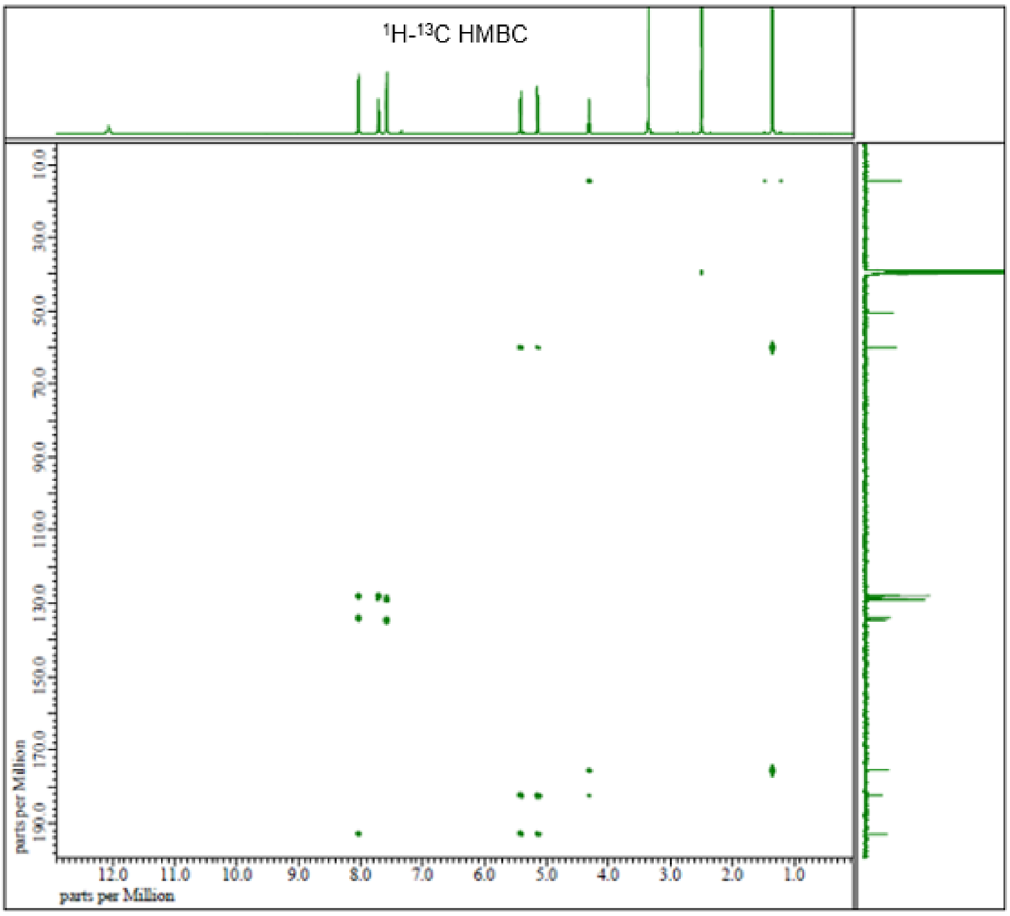
1H-13C HMBC
parts per Million
parts per Million

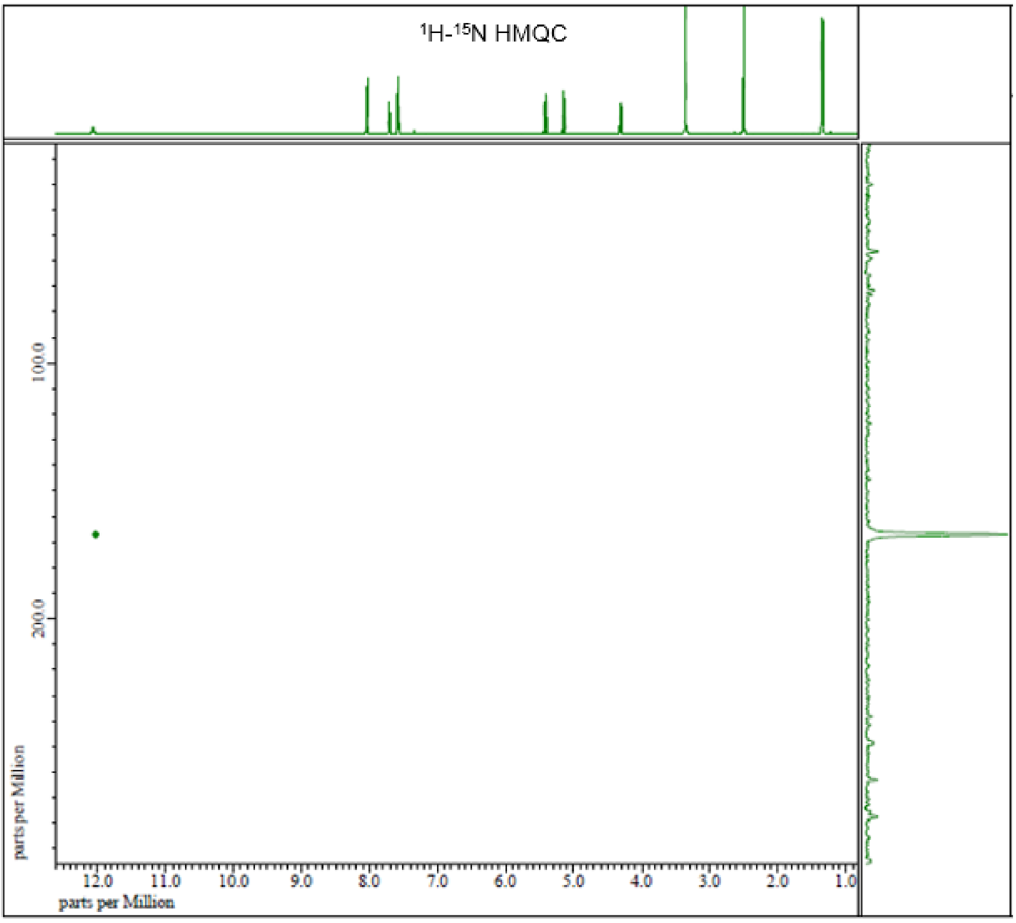
1H-15N HMQC
100.0
200.0
parts per Million
12.0 11.0 10.0 9.0 8.0 7.0 6.0 5.0 4.0 3.0 2.0 1.0
parts per Million

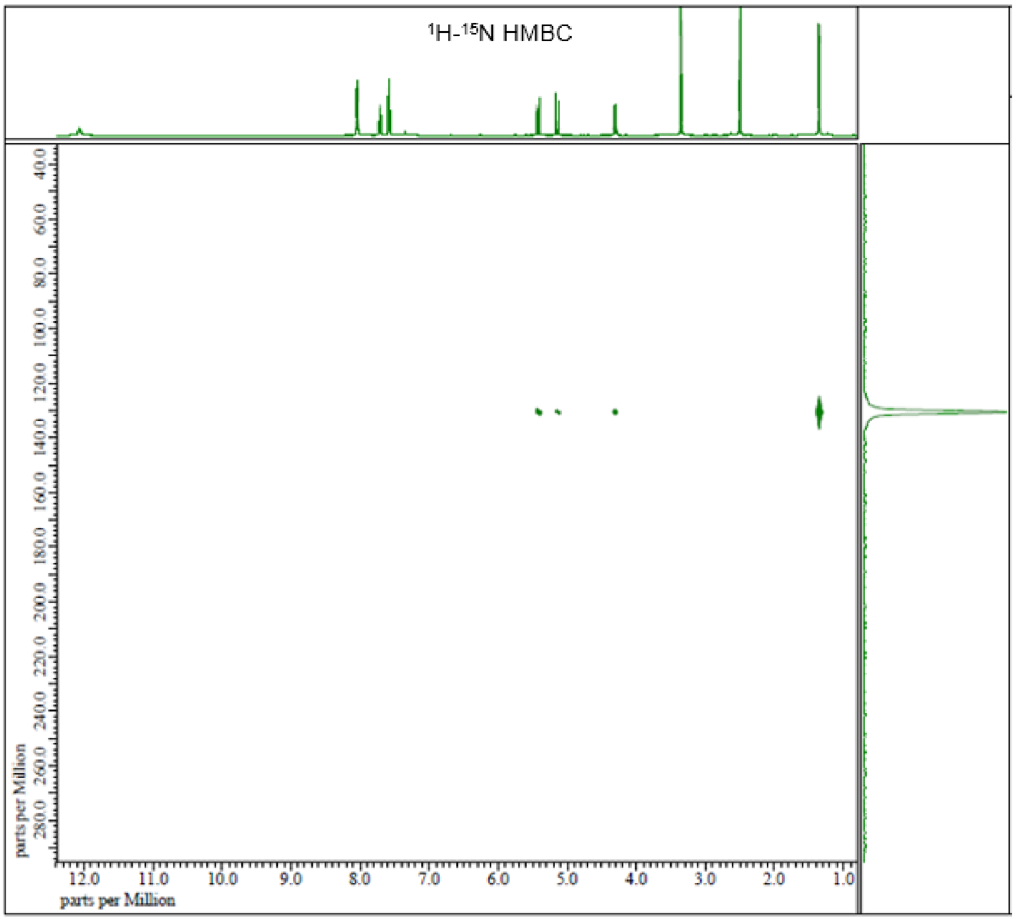
1H-15N HMBC
40.0 60.0 80.0 100.0 120.0 140.0 160.0 180.0 200.0 220.0 240.0 260.0 280.0
parts per Million
12.0 11.0 10.0 9.0 8.0 7.0 6.0 5.0 4.0 3.0 2.0 1.0
parts per Million

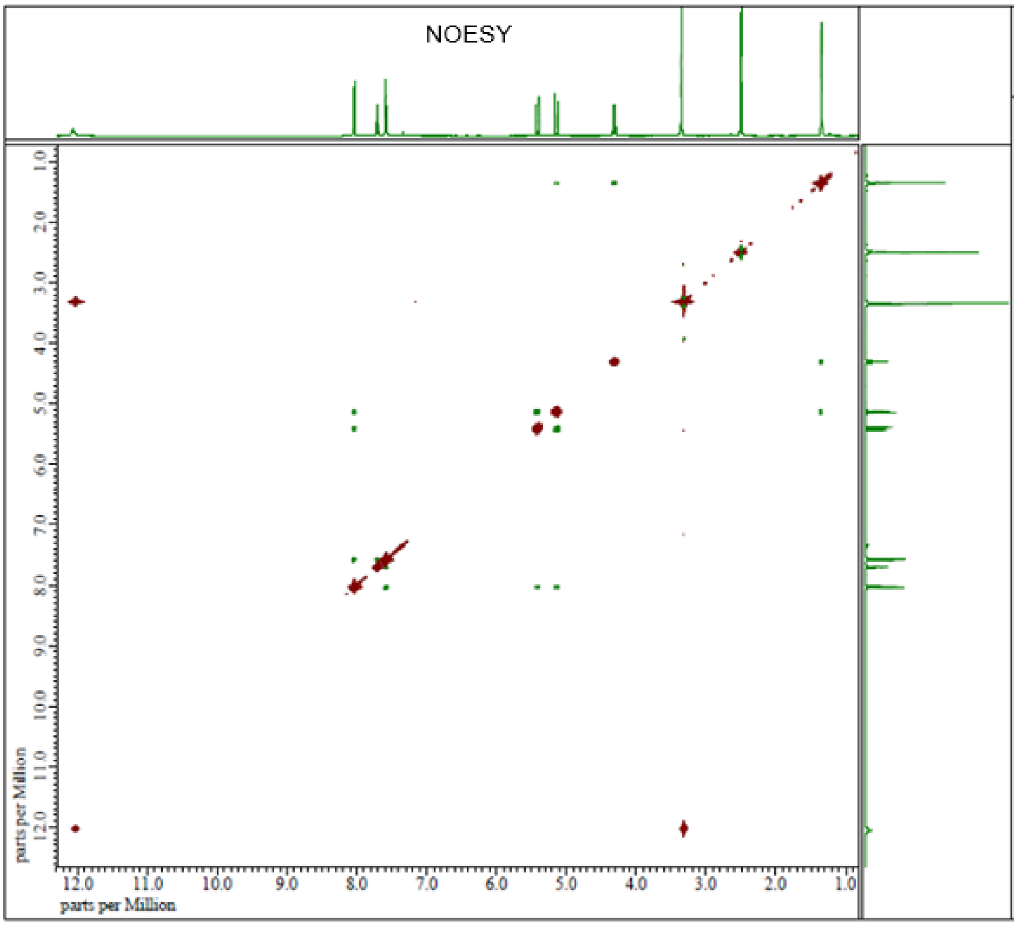

(5*S*)-1-(2-(4-bromophenyl)-2-oxoethyl)-5-methyl-2-thioxoimidazolidin-4-one 7{*5,1*}

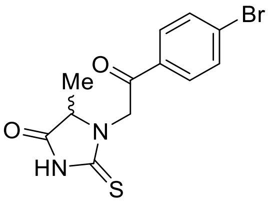

Brown amorphous solid, 12.2 mg (16%, 0.038 mmol). Cleaved from 517.5 mg of resin **2**{*5,1*} (0.460 mmol/g, 0.238 mmol of substrate). Final purity 98%. ^{1}H NMR (500 MHz, DMSO-d_6): δ = 12.07 (br. s, 1H), 7.97 (br. d, J = 8.7 Hz, 2H), 7.80 (br. d, J = 8.7 Hz, 2H), 5.38 (d, J = 18.2 Hz, 1H), 5.13 (d, J = 18.2 Hz, 1H), 4.30 (q, J = 7.2 Hz, 1H), 1.34 (d, J = 7.2 Hz, 3H). ^{13}C NMR (126 MHz, DMSO-d_6): δ = 192.26, 182.39, 175.52, 133.51, 131.95, 129.94, 128.03, 59.92, 50.28, 14.47. HRMS (ESI-TOF, pos.): *m/z* calcd for $C_{12}H_{12}BrN_2O_2S$ $[M+H]^+$ 326.9620, found 326.9628.

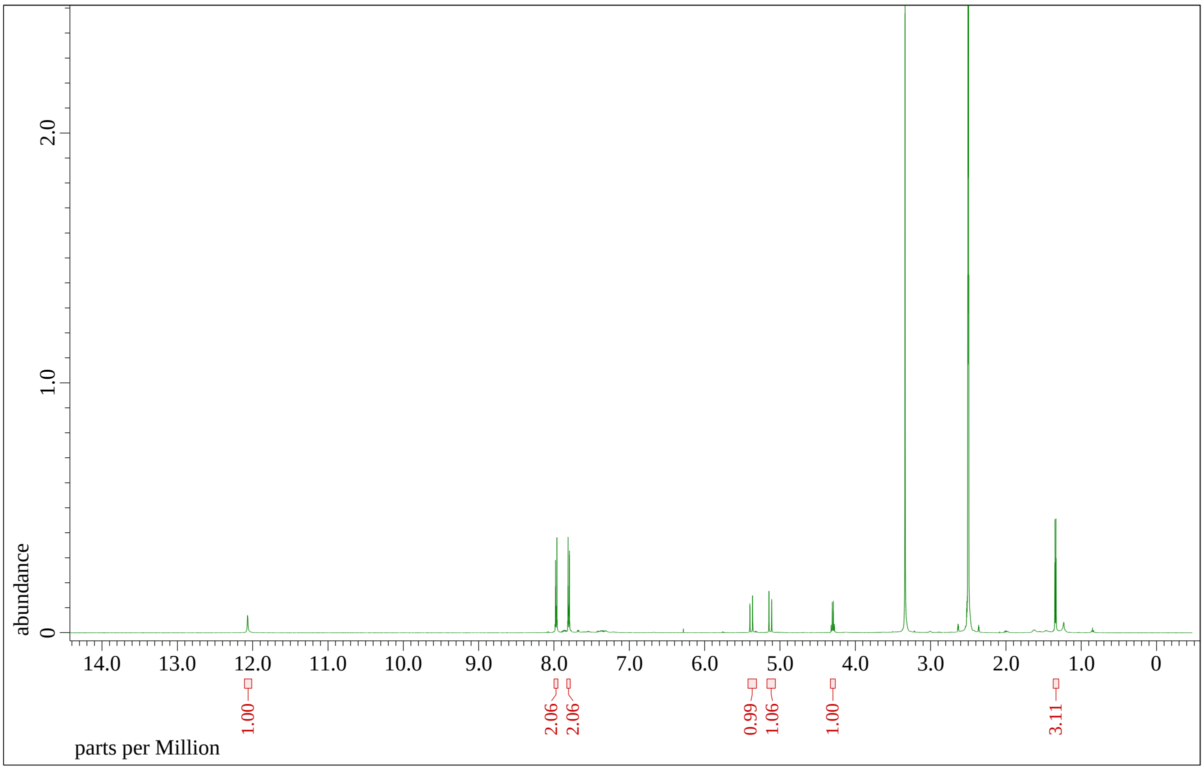
abundance
2.0
1.0
0
14.0 13.0 12.0 11.0 10.0 9.0 8.0 7.0 6.0 5.0 4.0 3.0 2.0 1.0 0
1.00
2.06
2.06
0.99
1.06
1.00
3.11
parts per Million

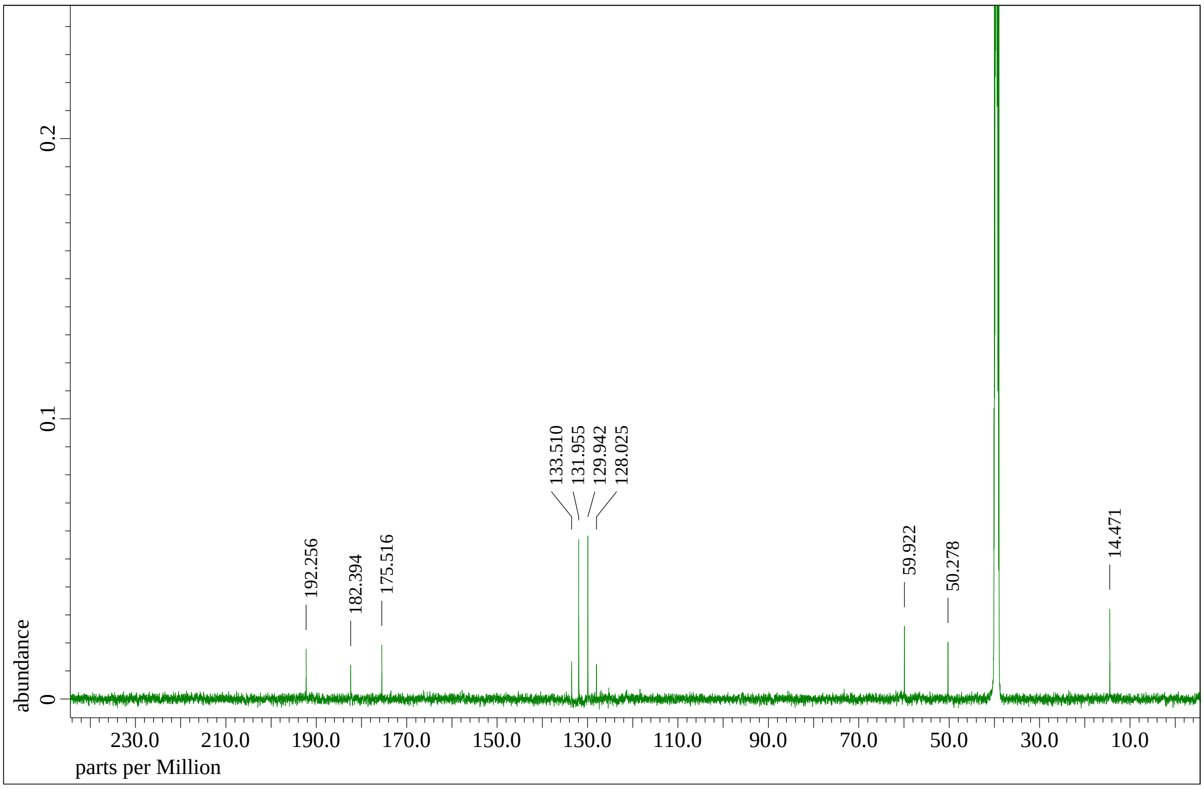
abundance
0.2
0.1
0
192.256
182.394
175.516
133.510
131.955
129.942
128.025
59.922
50.278
14.471
230.0 210.0 190.0 170.0 150.0 130.0 110.0 90.0 70.0 50.0 30.0 10.0
parts per Million

(5*S*)-5-methyl-1-(2-oxo-2-(thiophen-3-yl)ethyl)-2-thioxoimidazolidin-4-one 7{*7,1*}

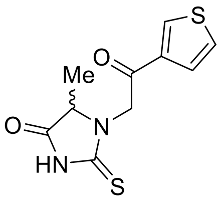

Creme amorphous solid, 14.6 mg (15%, 0.057 mmol). Cleaved from 637.0 mg of resin **2**{*7,1*} (0.460 mmol/g, 0.293 mmol of substrate). HPLC purity 99%. ^{1}H NMR (500 MHz, DMSO-d_6): δ = 12.11 (br. s, 1H), 8.68 (dd, *J* = 2.8, 1.2 Hz, 1H), 7.70 (dd, *J* = 5.0, 2.8 Hz, 1H), 7.56 (dd, *J* = 5.0, 1.2 Hz, 1H), 5.30 (d, *J* = 18.2 Hz, 1H), 5.02 (d, *J* = 18.2 Hz, 1H), 4.30 (q, *J* = 7.1 Hz, 1H), 1.34 (d, *J* = 7.1 Hz, 3H). ^{13}C NMR (126 MHz, DMSO-d_6): δ = 187.34, 182.35, 175.51, 139.06, 134.31, 127.78, 126.31, 59.94, 50.62, 14.47. HRMS (ESI-TOF, neg.): *m/z* calcd for $C_{10}H_9N_2O_2S_2$ $[M-H]^-$ 253.0100, found 253.0107.

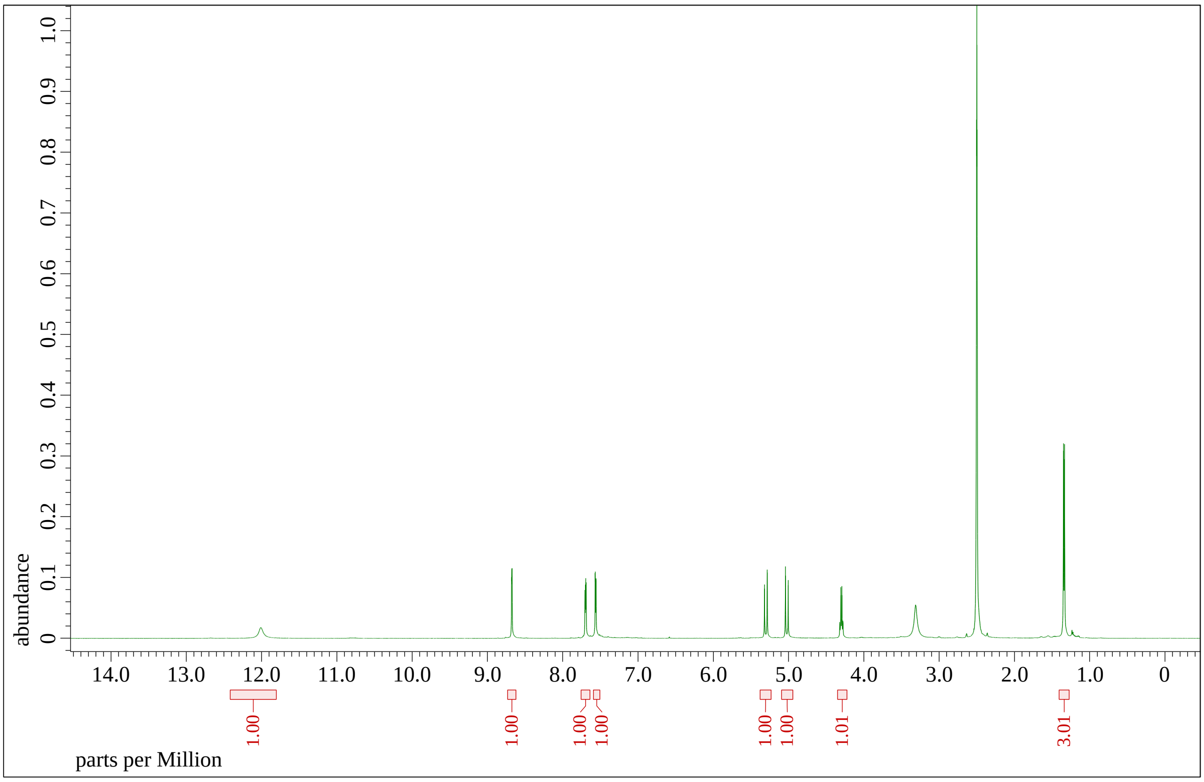

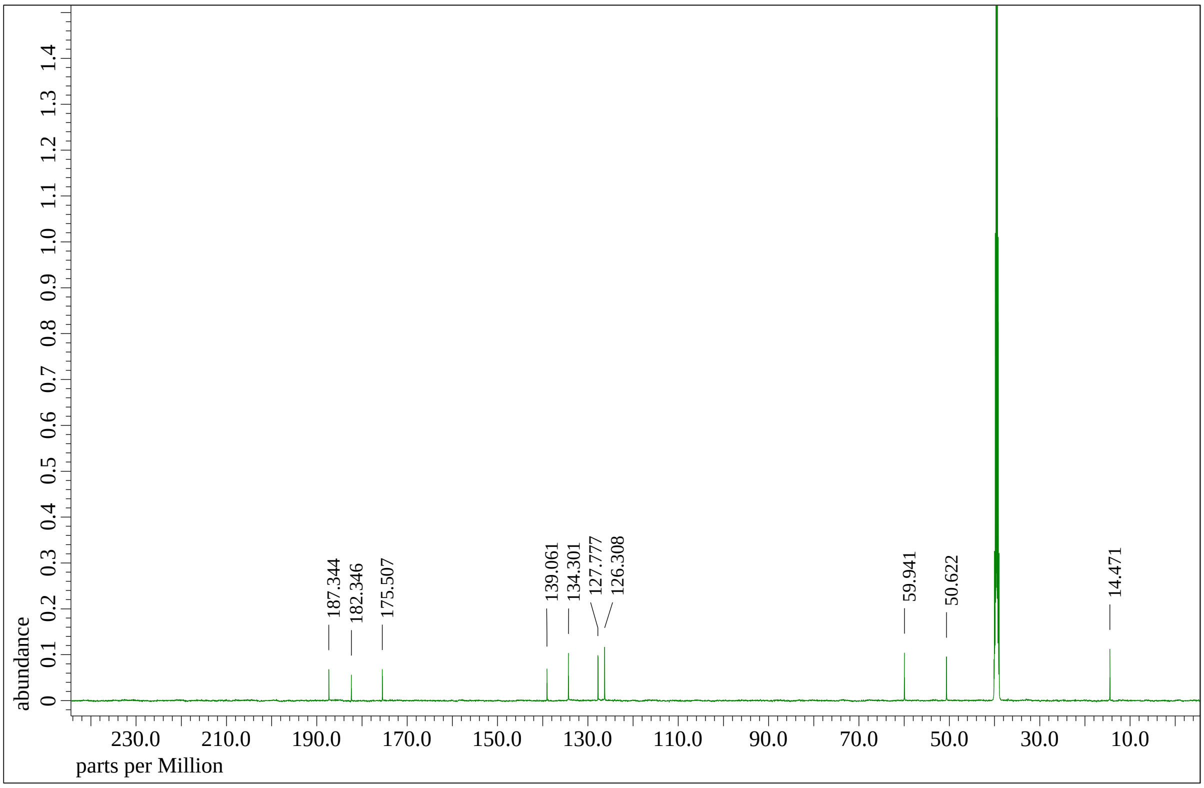

(3*R*)-7-benzyl-6-phenyl-2,3-dihydroimidazo[2,1-*b*]thiazol-7-ium-3-carboxylate 8{*1,2*}

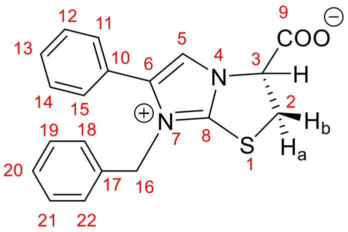

NMR: Mixture with 7% of **9**{*1,2*}. Creme amorphous solid, 19.0 mg (53%, 0.057 mmol). Cleaved from 212.8 mg of resin **2**{*1,2*} (0.503 mmol/g, 0.107 mmol of substrate). HPLC purity 98%. ^{1}H NMR (500 MHz, DMSO-d_6): δ = 7.93 (s, 1H, HC^{5}), 7.47-7.52 (m, 5H, HC$^{11-15}$), 7.34-7.40 (m, 3H, HC$^{19-21}$), 7.00-7.12 (m, 2H, HC^{18,22}), 5.28 (s, 2H, HC^{16}), 4.96 (dd, *J* = 8.6, 6.2 Hz, 1H, HC^{3}), 4.29 (dd, *J* = 11.1, 8.6 Hz, 1H, H$_b$C^{2}), 4.23 (dd, *J* = 11.1, 6.2 Hz, 1H, H$_a$C^{2}). ^{13}C NMR (126 MHz, DMSO-d_6): δ = 165.49 (C9), 150.04 (C8), 136.50 (C6), 133.19 (C17), 129.86 (C13), 129.11 (C12,14), 129.09 (C11,15), 128.86 (C19,21), 128.59 (C20), 127.67 (C18,22), 126.20 (C10), 119.30 (C5), 63.99 (C3), 50.13 (C16), 41.59 (C2). ^{15}N NMR (51 MHz, DMSO-d_6): δ = 189.1 (N4); 162.6 (N7). HRMS (ESI-TOF, neg.): *m/z* calcd for $C_{19}H_{15}N_2O_2S$ [M-H]$^-$ 335.0849, found 335.0846.

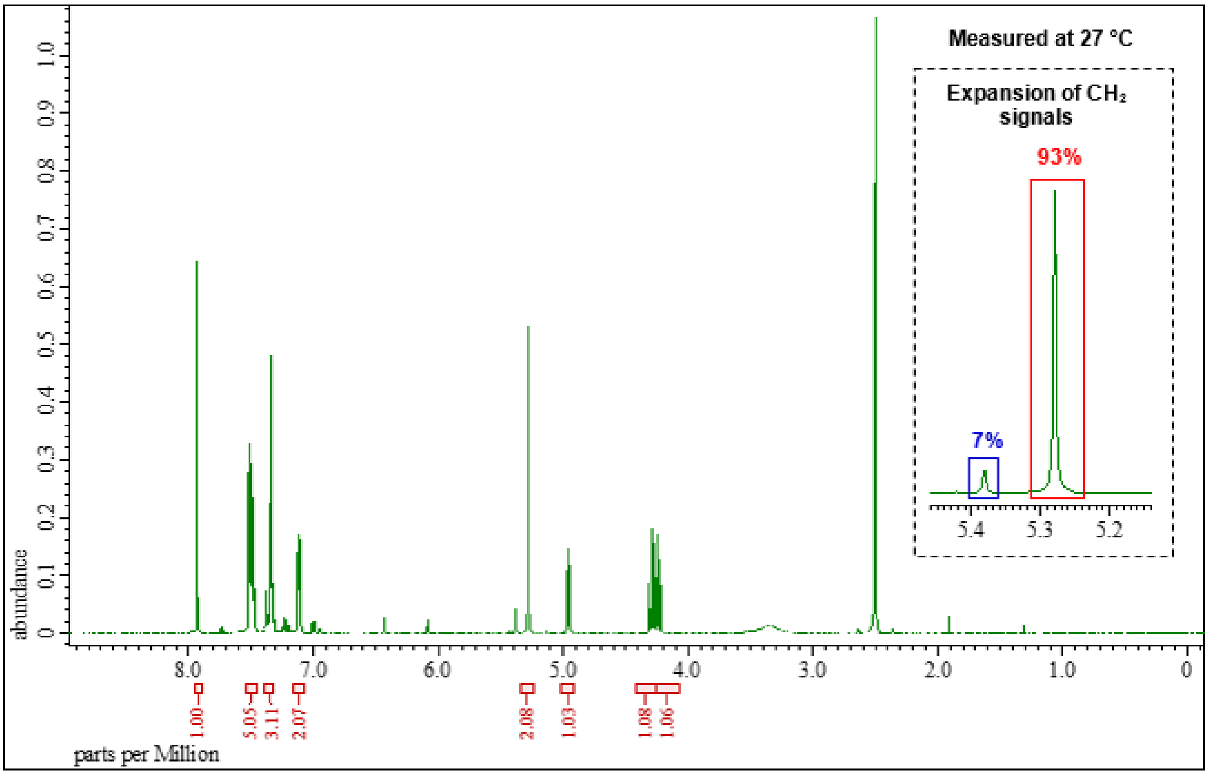
Measured at 27 °C
Expansion of CH₂ signals
93%
7%
5.4
5.3
5.2
abundance
0
0.1
0.2
0.3
0.4
0.5
0.6
0.7
0.8
0.9
1.0
8.0
7.0
6.0
5.0
4.0
3.0
2.0
1.0
0
1.00
5.05
3.11
2.07
2.08
1.03
1.08
1.06
parts per Million

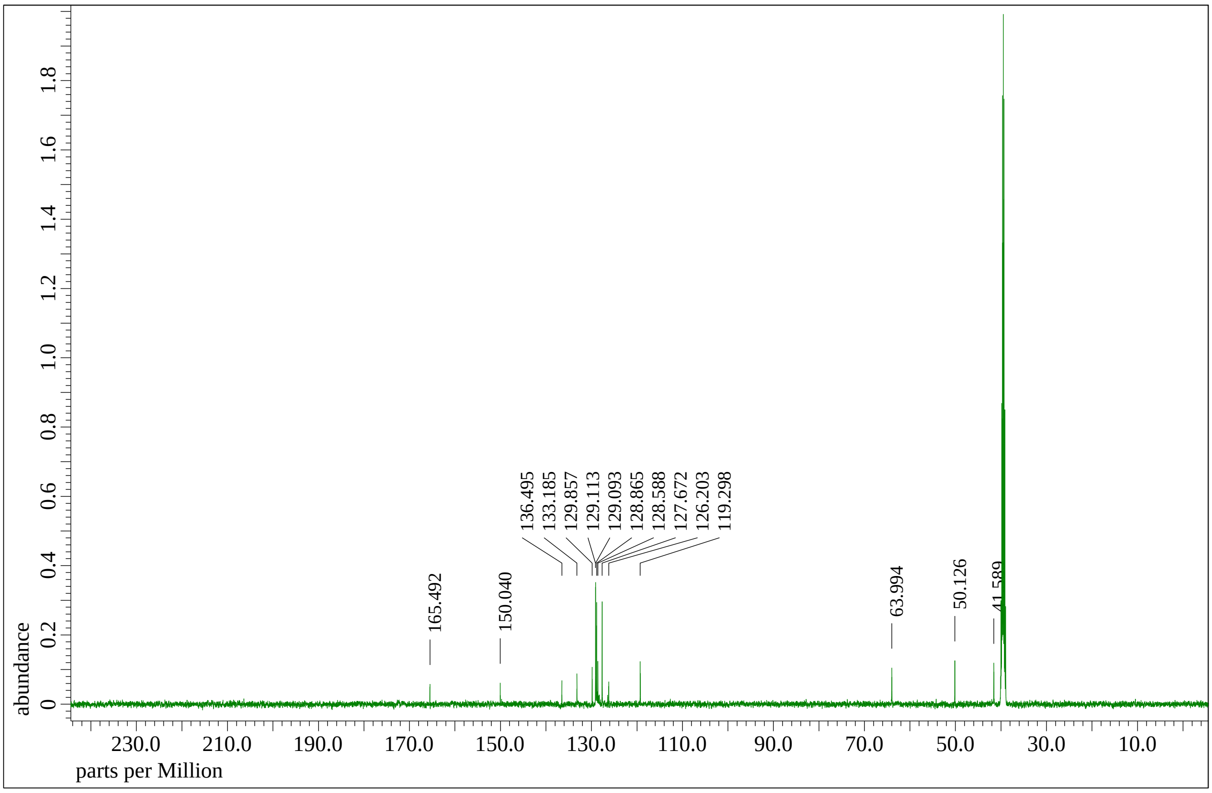
abundance
0
0.2
0.4
0.6
0.8
1.0
1.2
1.4
1.6
1.8
165.492
150.040
136.495
133.185
129.857
129.113
129.093
128.865
128.588
127.672
126.203
119.298
63.994
50.126
41.589
230.0
210.0
190.0
170.0
150.0
130.0
110.0
90.0
70.0
50.0
30.0
10.0
parts per Million

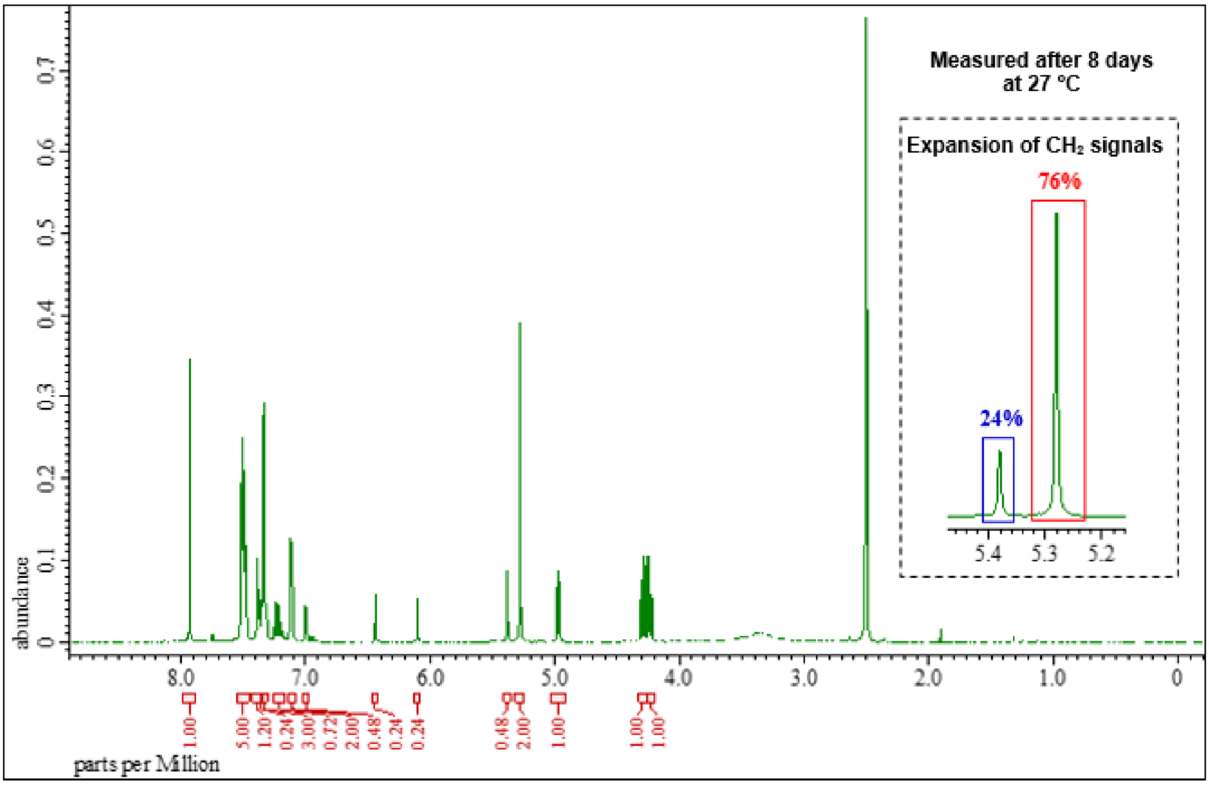
Measured after 8 days
at 27 °C
Expansion of CH₂ signals
76%
24%
5.4
5.3
5.2
abundance
0
0.1
0.2
0.3
0.4
0.5
0.6
0.7
8.0
7.0
6.0
5.0
4.0
3.0
2.0
1.0
0
1.00
5.00
1.20
0.24
3.00
0.72
2.00
0.48
0.24
0.24
0.48
2.00
1.00
1.00
1.00
parts per Million

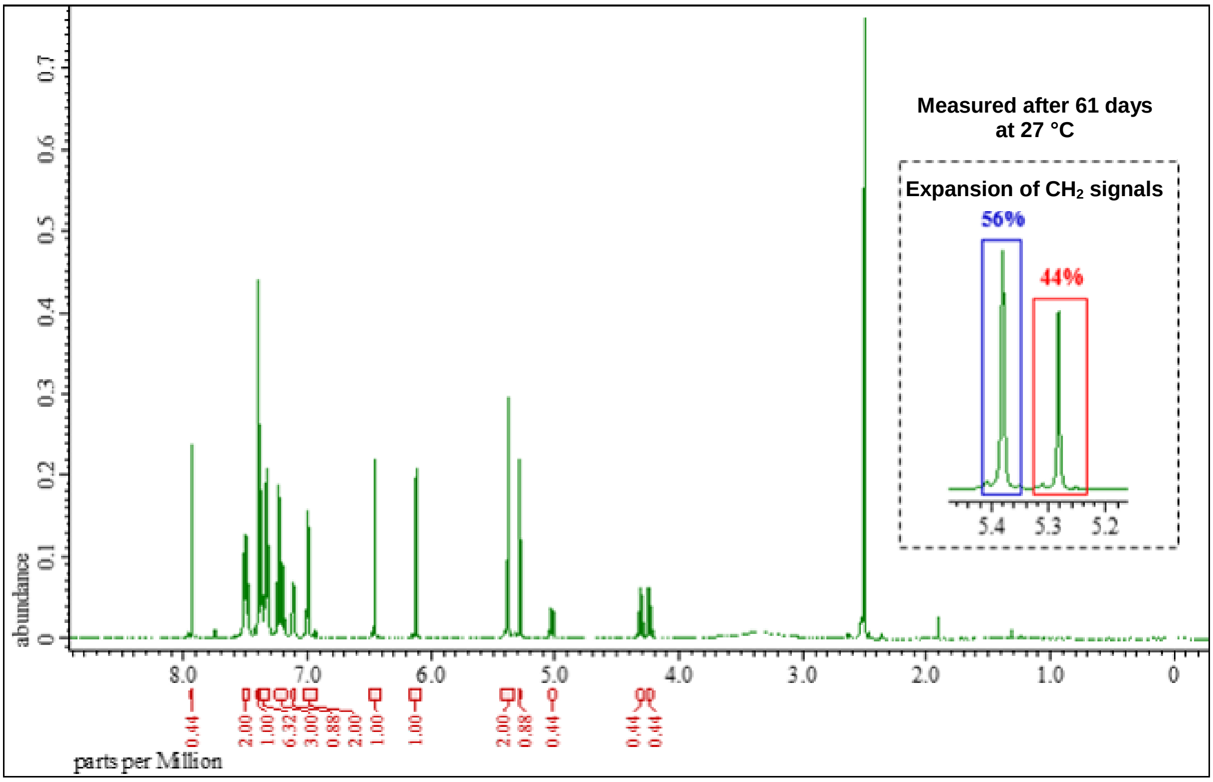
Measured after 61 days
at 27 °C
Expansion of CH₂ signals
56%
44%
5.4
5.3
5.2
abundance
0
0.1
0.2
0.3
0.4
0.5
0.6
0.7
8.0
7.0
6.0
5.0
4.0
3.0
2.0
1.0
0
0.44
2.00
1.00
6.32
3.00
0.88
2.00
1.00
1.00
2.00
0.88
0.44
0.44
0.44
parts per Million

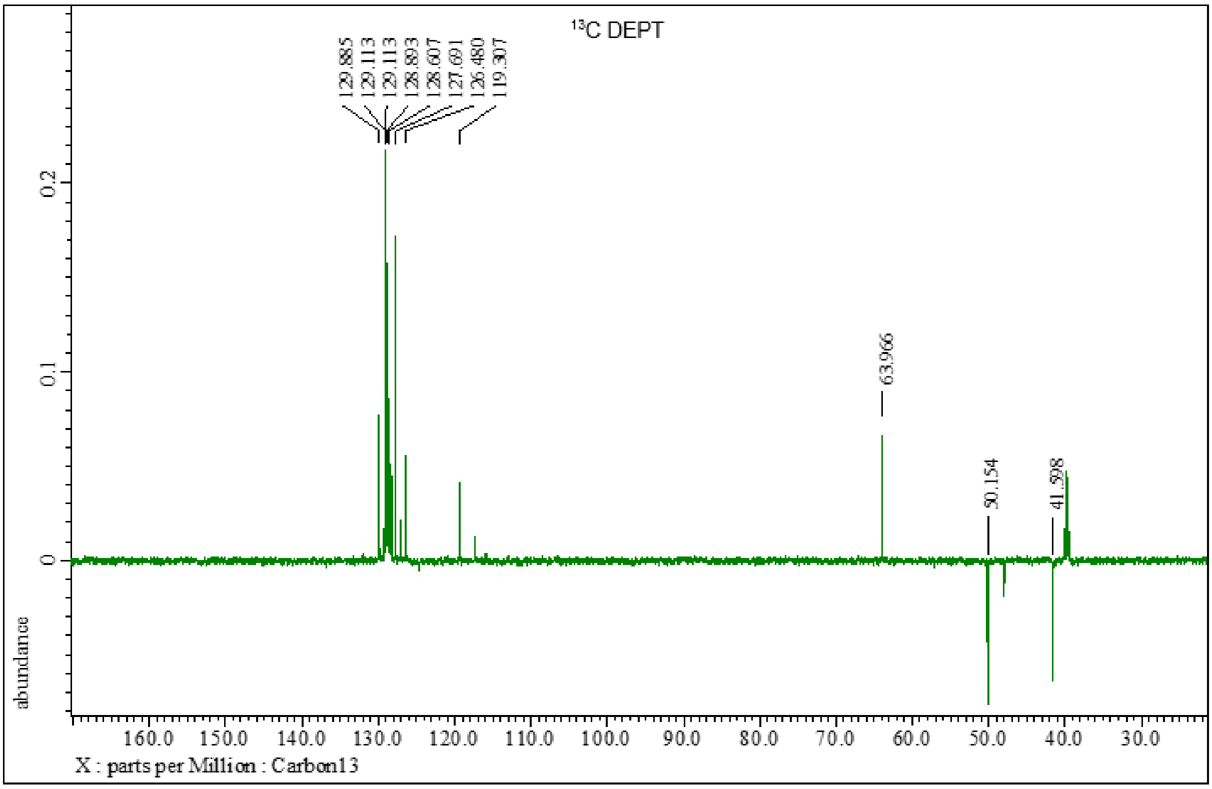

^{13}C DEPT
129.885
129.113
129.113
128.893
128.607
127.691
126.480
119.307
63.966
50.154
41.598
0.2
0.1
0
abundance
160.0 150.0 140.0 130.0 120.0 110.0 100.0 90.0 80.0 70.0 60.0 50.0 40.0 30.0
X : parts per Million : Carbon13

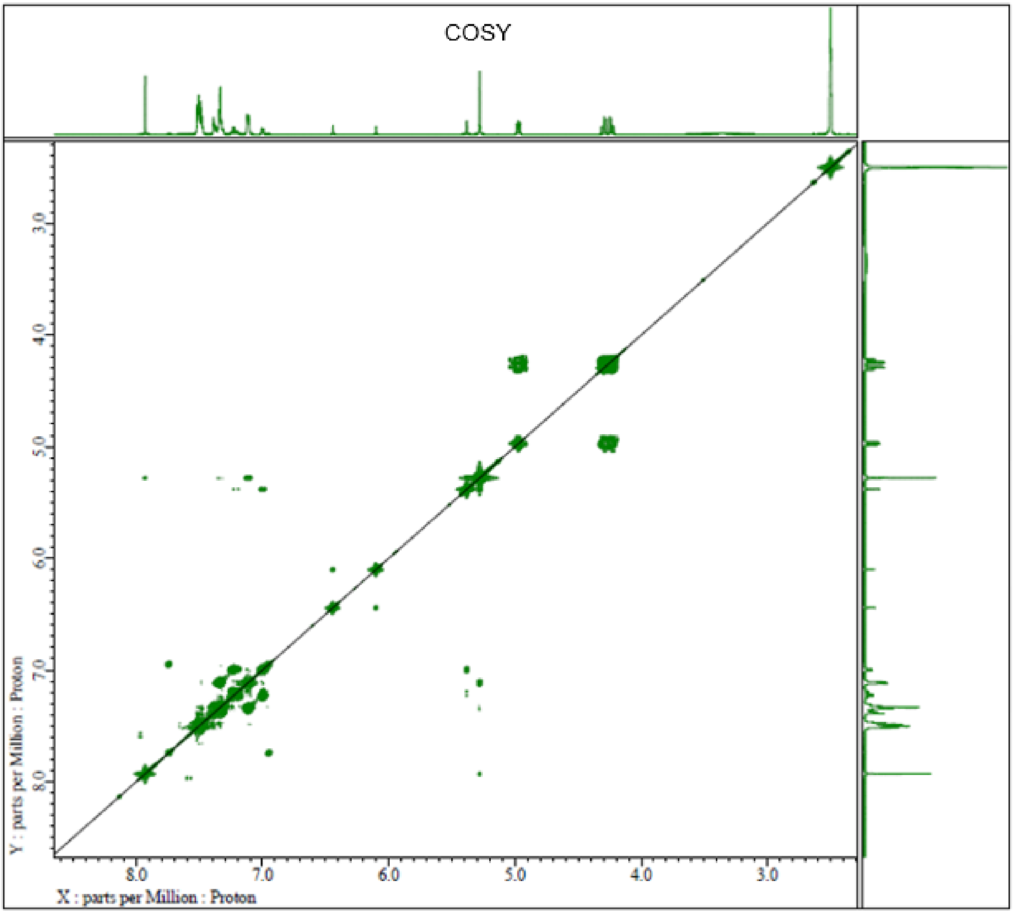

COSY
3.0
4.0
5.0
6.0
7.0
8.0
Y : parts per Million : Proton
8.0 7.0 6.0 5.0 4.0 3.0
X : parts per Million : Proton

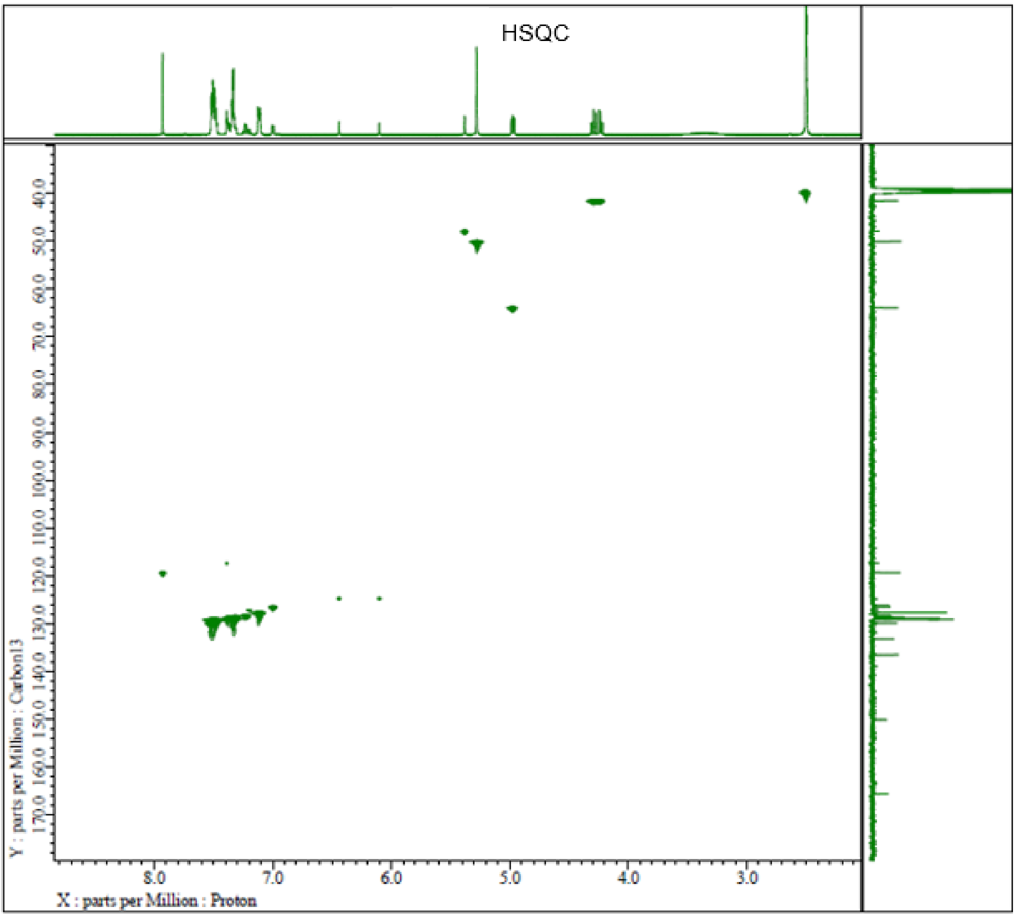
HSQC
Y : parts per Million : Carbon13
40.0
50.0
60.0
70.0
80.0
90.0
100.0
110.0
120.0
130.0
140.0
150.0
160.0
170.0
8.0
7.0
6.0
5.0
4.0
3.0
X : parts per Million : Proton

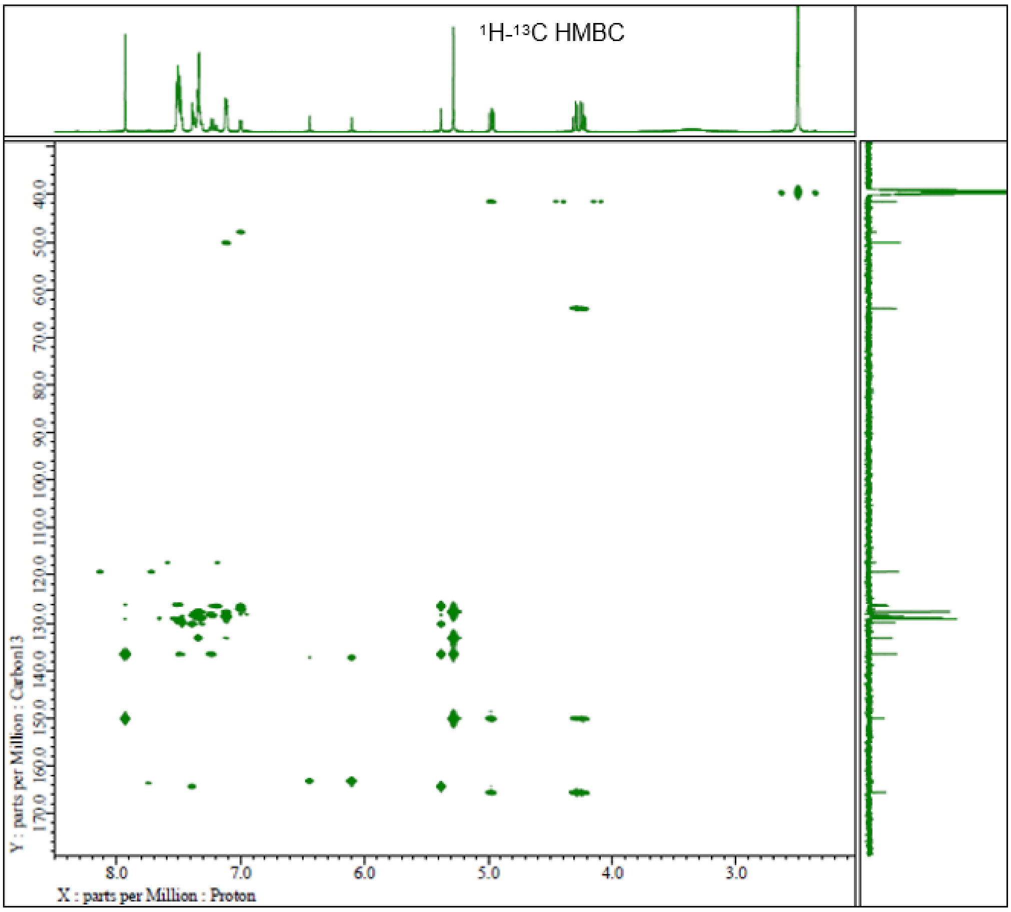
1H-13C HMBC
Y : parts per Million : Carbon13
40.0
50.0
60.0
70.0
80.0
90.0
100.0
110.0
120.0
130.0
140.0
150.0
160.0
170.0
8.0
7.0
6.0
5.0
4.0
3.0
X : parts per Million : Proton

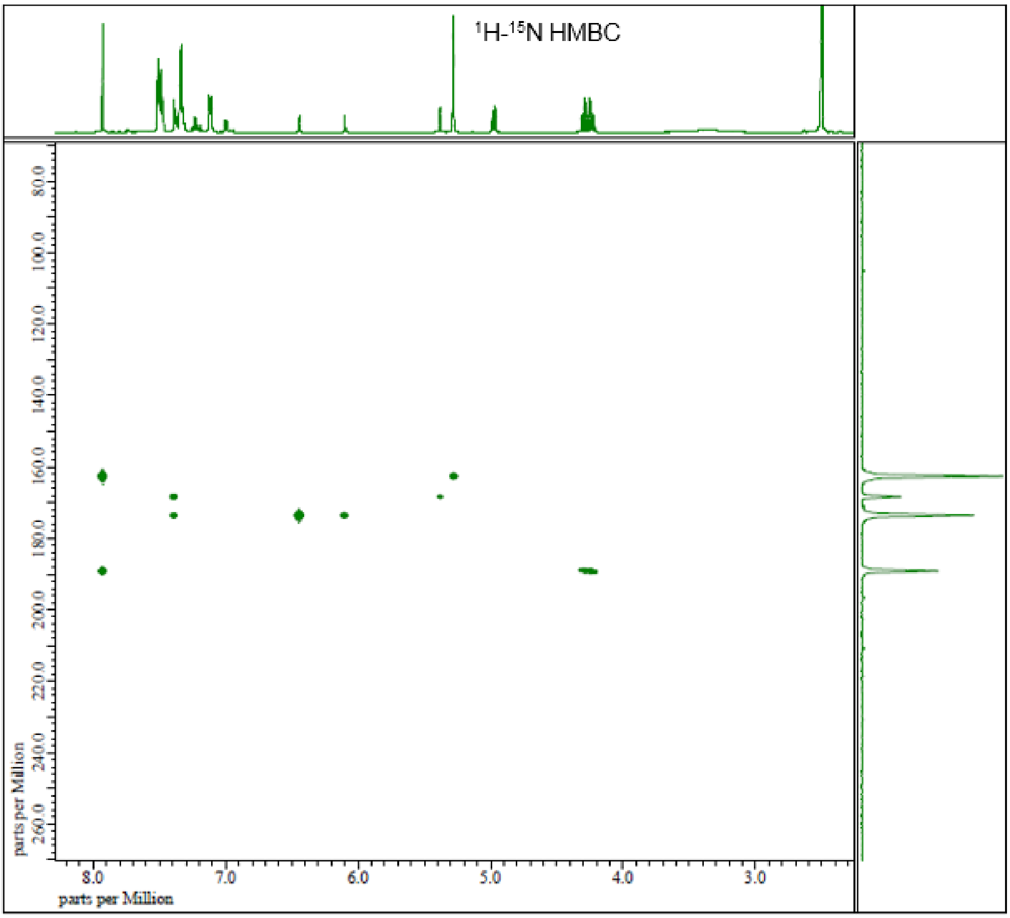

^{1}H-^{15}N HMBC
parts per Million
parts per Million

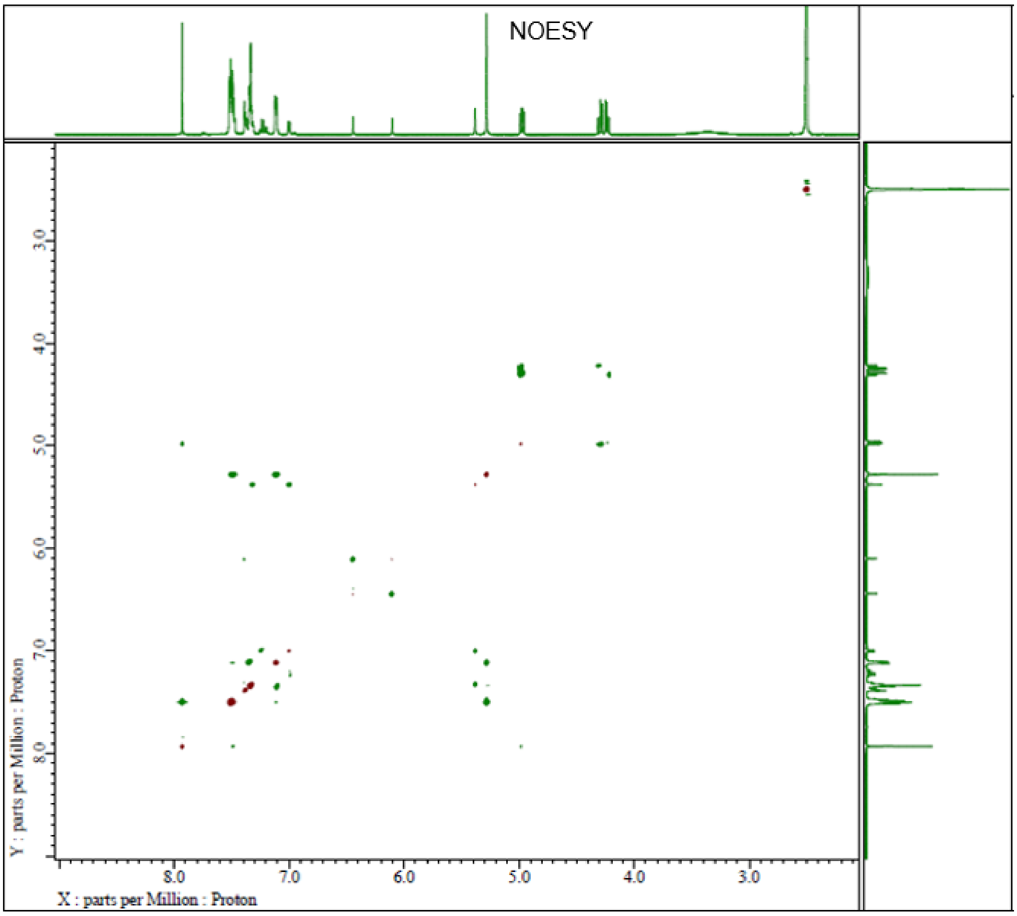

NOESY
Y : parts per Million : Proton
X : parts per Million : Proton

2-(3-benzyl-4-phenyl-2-thioxo-2,3-dihydro-1*H*-imidazol-1-yl)acrylic acid 9{*1,2*}

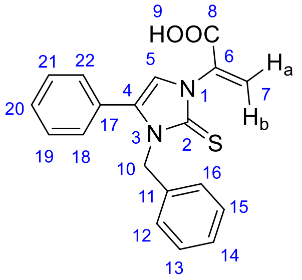

NMR purity 99%, yield quantitative (calculated from **8**{*1,2*}). ^{1}H NMR (500 MHz, DMSO-d_6): δ = 13.28 (br. s, 1H, HO^9), 7.41 (s, 1H, HC^5), 7.37-7.39 (m, 3H, HC^{19-21}), 7.31-7.33 (m, 2H, $HC^{18,22}$), 7.18-7.25 (m, 3H, HC^{13-15}), 6.98-7.01 (m, 2H, $HC^{12,16}$), 6.47 (d, J = 0.7 Hz, 1H, H_aC^7), 6.14 (d, J = 0.7 Hz, 1H, H_bC^7), 5.38 (s, 2H, HC^{10}). ^{13}C NMR (126 MHz, DMSO-d_6): δ = 164.39 (C2), 163.08 (C8), 136.65 (C6), 136.48 (C11), 130.37 (C4), 128.88 (C20), 128.72 (C19,21), 128.45 (C18,22), 128.30 (C13,15), 127.92 (C17), 127.12 (C14), 126.47 (C12,16), 125.28 (C7), 117.20 (C5), 47.95 (C10). ^{15}N NMR (51 MHz, DMSO-d_6): δ = 173.5 (N1); 168.3 (N3). The 2D assignments are made from the previous sample **8**{*1,2*} presented as the mixture with 24% of **9**{*1,2*}. HRMS (ESI-TOF, neg.): *m/z* calcd for $C_{19}H_{15}N_2O_2S$ $[M-H]^-$ 335.0849, found 335.0846.

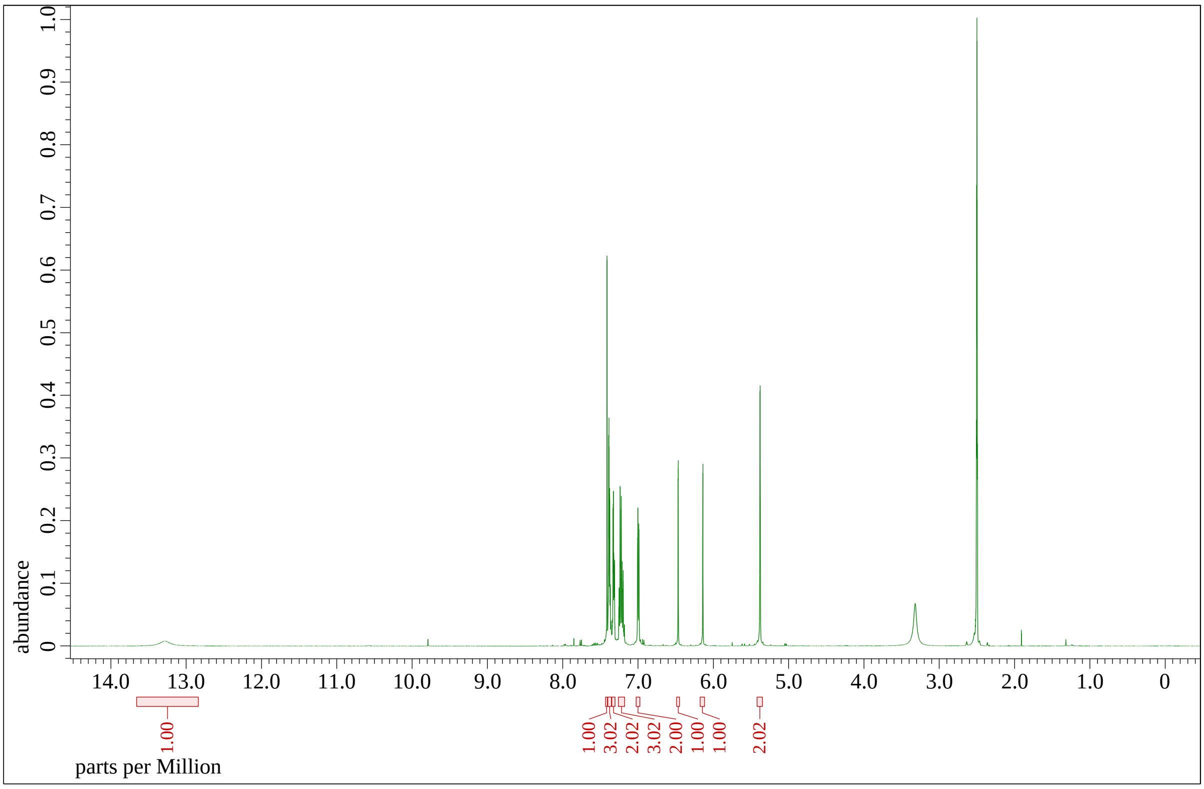

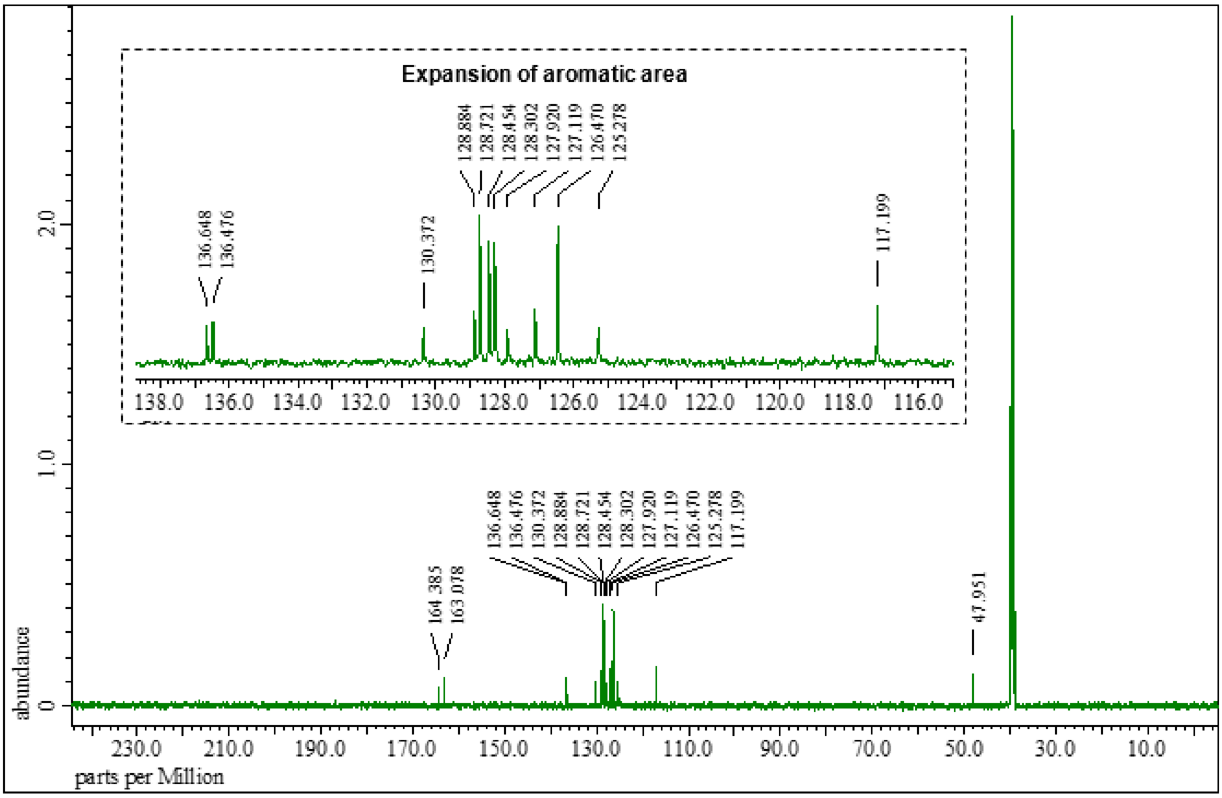

(3*R*)-7-allyl-6-phenyl-2,3-dihyhydroimidazo[2,1-*b*]thiazol-7-ium-3-carboxylate 8{*1,3*}

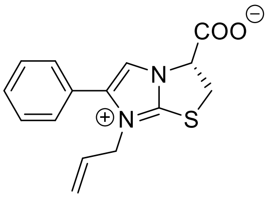

Creme amorphous solid, 17.7 mg (34%, 0.062 mmol). Cleaved from 316.9 mg of resin **2**{*1,3*} (0.575 mmol/g, 0.182 mmol of substrate). HPLC purity 98%. ^{1}H NMR (500 MHz, DMSO-*d*$_6$): δ = 7.91 (s, 1H), 7.50-7.55 (m, 5H), 5.88-5.94 (m, 1H), 5.33 (br. d, *J* = 10.4 Hz, 1H), 5.14 (br. d, *J* = 17.1 Hz, 1H), 4.96 (dd, *J* = 8.4, 6.0 Hz, 1H), 4.64 (ddd, *J* = 4.7, 1.4, 1.4 Hz, 2H), 4.34 (dd, *J* = 11.0, 8.4 Hz, 1H), 4.28 (dd, *J* = 11.0, 6.0 Hz, 1H). ^{13}C NMR (126 MHz, DMSO-*d*$_6$): δ = 165.55, 149.92, 136.48, 130.23, 129.84, 129.16, 128.86, 126.17, 119.56, 118.98, 64.07, 49.00, 41.68. HRMS (ESI-TOF, pos.): *m/z* calcd for $C_{15}H_{15}N_2O_2S$ [M+H]$^+$ 287.0849, found 287.0847.

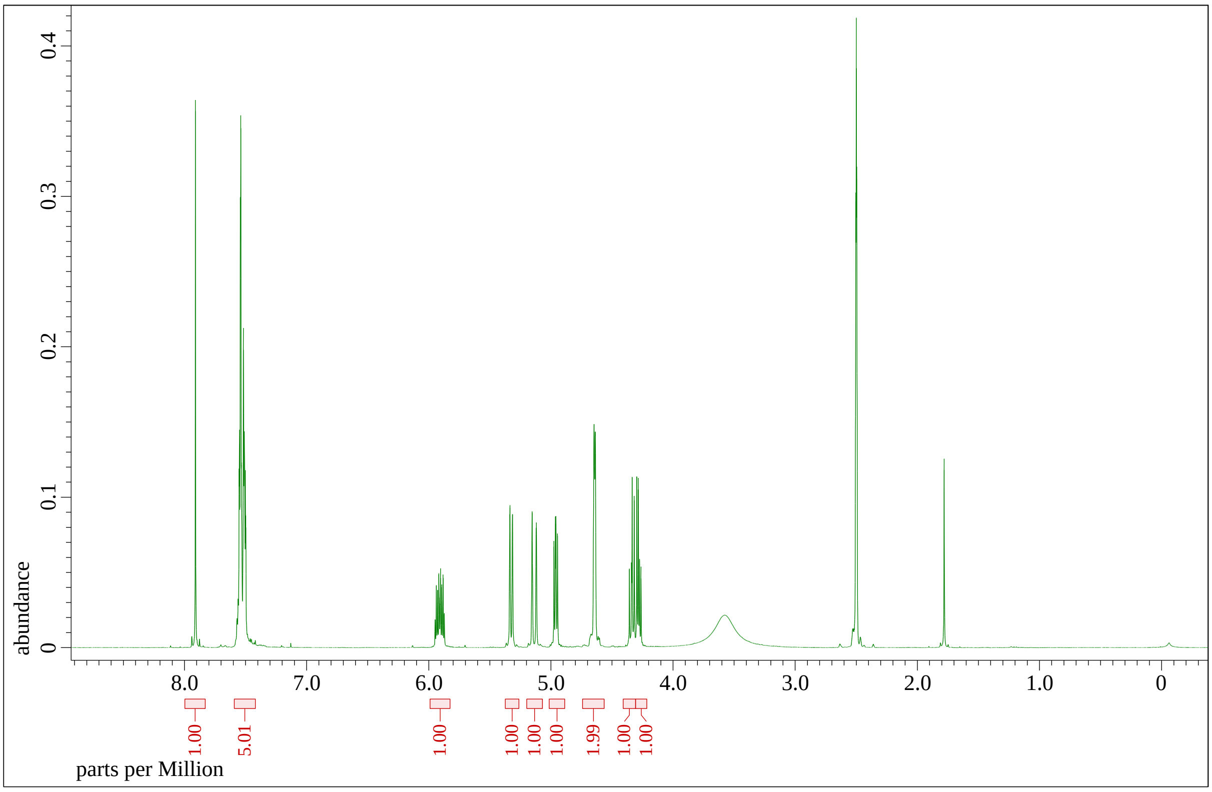
abundance
0
0.1
0.2
0.3
0.4
8.0
7.0
6.0
5.0
4.0
3.0
2.0
1.0
0
1.00
5.01
1.00
1.00
1.00
1.00
1.99
1.00
1.00
parts per Million

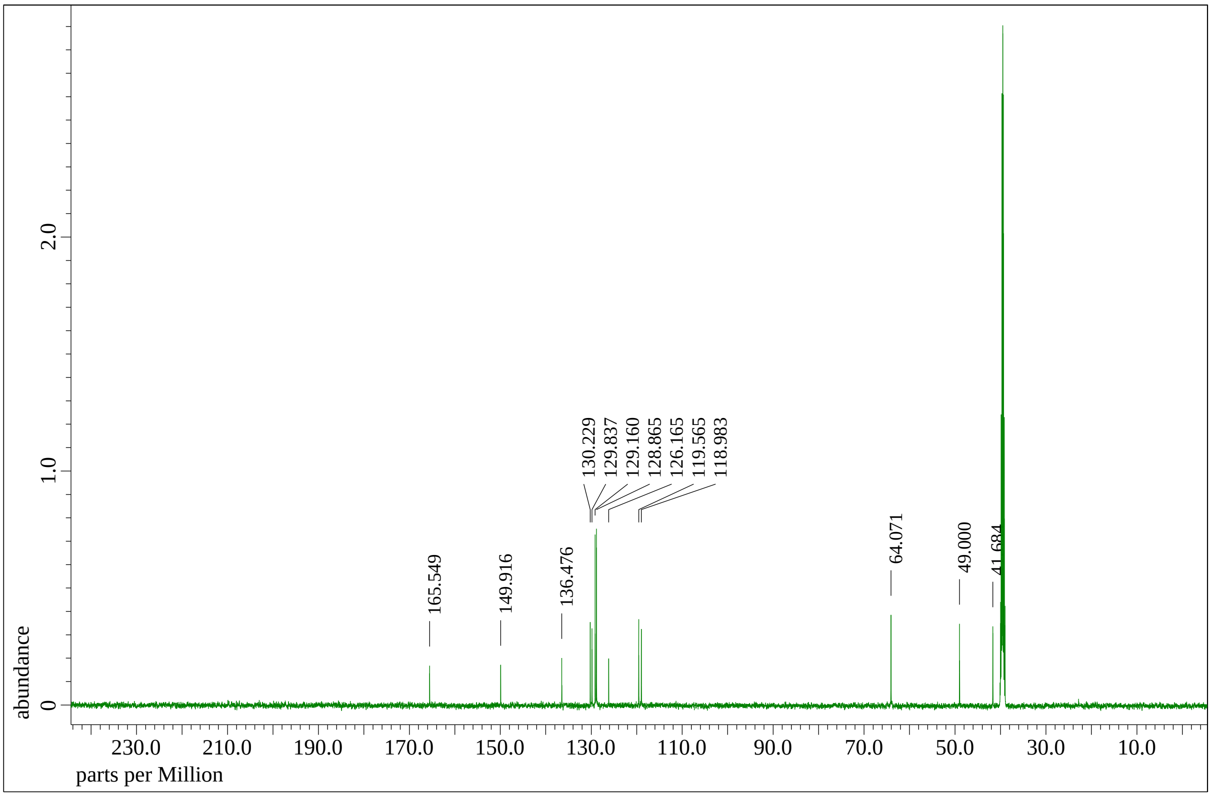
abundance
0
1.0
2.0
165.549
149.916
136.476
130.229
129.837
129.160
128.865
126.165
119.565
118.983
64.071
49.000
41.684
230.0
210.0
190.0
170.0
150.0
130.0
110.0
90.0
70.0
50.0
30.0
10.0
parts per Million

2-(3-allyl-4-phenyl-2-thioxo-2,3-dihydro-1*H*-imidazol-1-yl)acrylic acid 9{*1,3*}

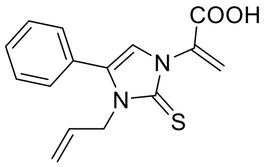

NMR: Mixture with 8% of **8**{*1,3*}. ^{1}H NMR (500 MHz, DMSO-d_6): δ = 7.44-7.50 (m, 5H), 7.35 (s, 1H), 6.42 (d, J = 0.7 Hz, 1H), 6.05 (d, J = 0.7 Hz, 1H), 5.78-5.84 (m, 1H), 5.10 (br. d, J = 10.4 Hz, 1H), 4.84 (br. d, J = 17.2 Hz, 1H), 4.71 (ddd, J = 4.8, 1.8, 1.8 Hz, 2H). ^{13}C NMR (126 MHz, DMSO-d_6): δ = 163.68, 163.20, 137.13, 132.34, 130.18, 128.86, 128.82, 128.44, 128.05, 124.60, 116.98, 116.89, 47.10. HRMS (ESI-TOF, pos.): *m/z* calcd for $C_{15}H_{15}N_2O_2S$ [M+H]$^+$ 287.0849, found 287.0847.

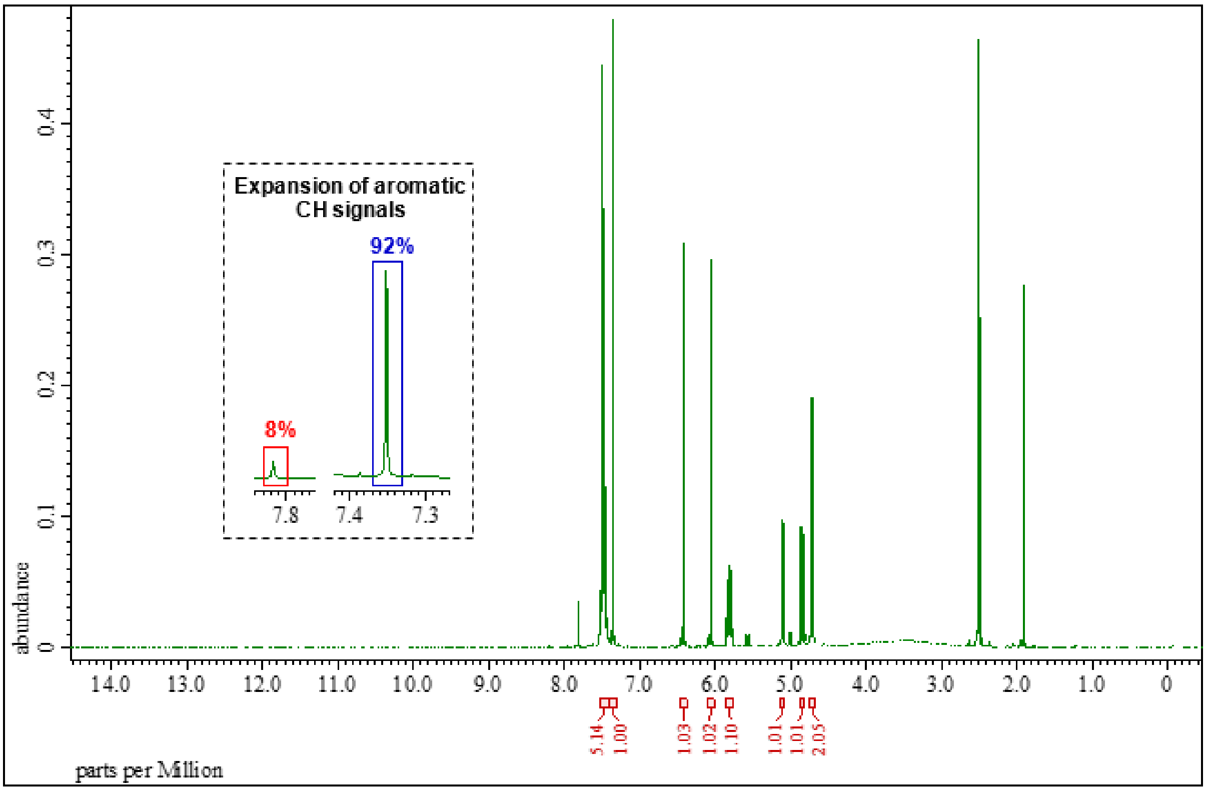

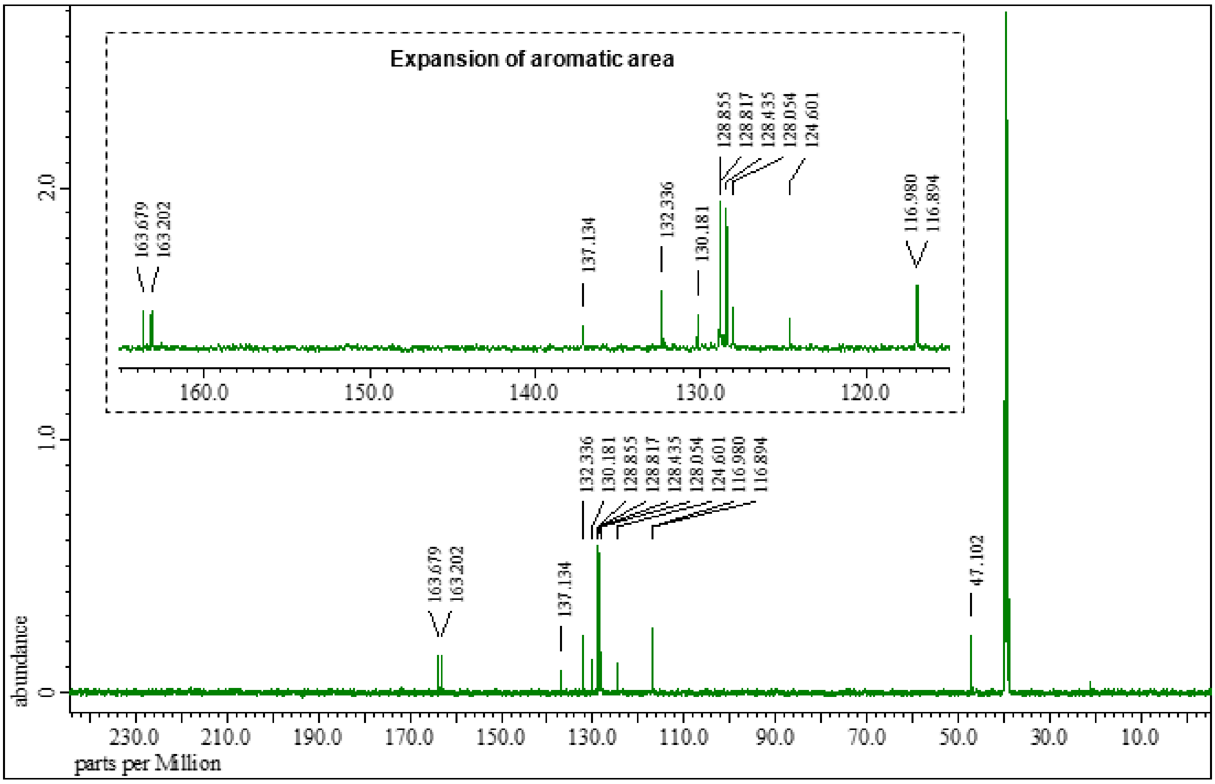

(3*R*)-7-ethyl-6-phenyl-2,3-dihyhydroimidazo[2,1-*b*]thiazol-7-ium-3-carboxylate 8{*1,4*}

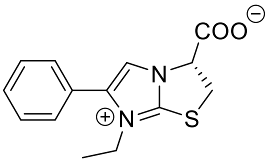

NMR: Mixture with 55% of **9**{*1,4*}. Creme amorphous solid, 11.4 mg (23%, 0.042 mmol) of which **8**{*1,4*} 4.3 mg (9%, 0.016 mmol) and **9**{*1,4*} 7.1 mg (14%, 0.026 mmol). Cleaved from 312.8 mg of resin **2**{*1,4*} (0.575 mmol/g, 0.180 mmol of substrate). HPLC purity 99%. ^{1}H NMR (500 MHz, DMSO-d_6): δ = 7.88 (s, 1H), 7.47-7.51 (m, 5H), 5.09 (m, 1H), 4.40 (dd, *J* = 11.1, 8.4 Hz, 1H), 4.32 (dd, *J* = 11.1, 6.0 Hz, 1H), 4.05 (q, *J* = 7.3 Hz, 2H), 1.22 (t, *J* = 7.3 Hz, 3H). HRMS (ESI-TOF, pos.): *m/z* calcd for $C_{14}H_{15}N_2O_2S$ [M+H]$^+$ 275.0849, found 275.0848.

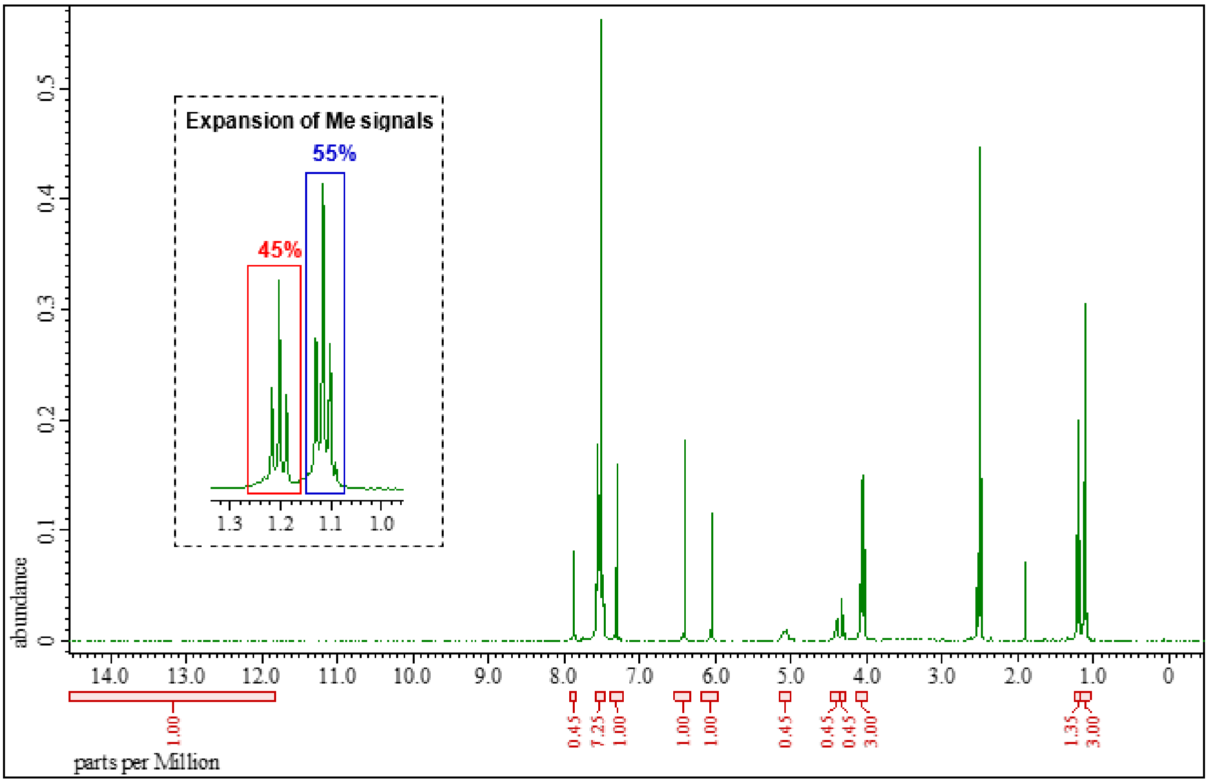

2-(3-ethyl-4-phenyl-2-thioxo-2,3-dihydro-1*H*-imidazol-1-yl)acrylic acid 9{*1,4*}

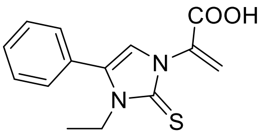

NMR: Mixture with 11% of **8**{*1,4*}. ^{1}H NMR (500 MHz, DMSO-d_6): δ = 13.24 (br. s, 1H), 7.48-7.53 (m, 5H), 7.32 (s, 1H), 6.41 (d, J = 0.7 Hz, 1H), 6.06 (d, J = 0.7 Hz, 1H), 4.06 (q, J = 7.1 Hz, 2H), 1.12 (t, J = 7.1 Hz, 3H). ^{13}C NMR (126 MHz, DMSO-d_6): δ = 163.13, 162.78, 136.51, 129.97, 128.97, 128.92, 128.68, 128.08, 125.00, 116.85, 40.03, 13.39. HRMS (ESI-TOF, pos.): *m/z* calcd for $C_{14}H_{15}N_2O_2S$ $[M+H]^+$ 275.0849, found 275.0848.

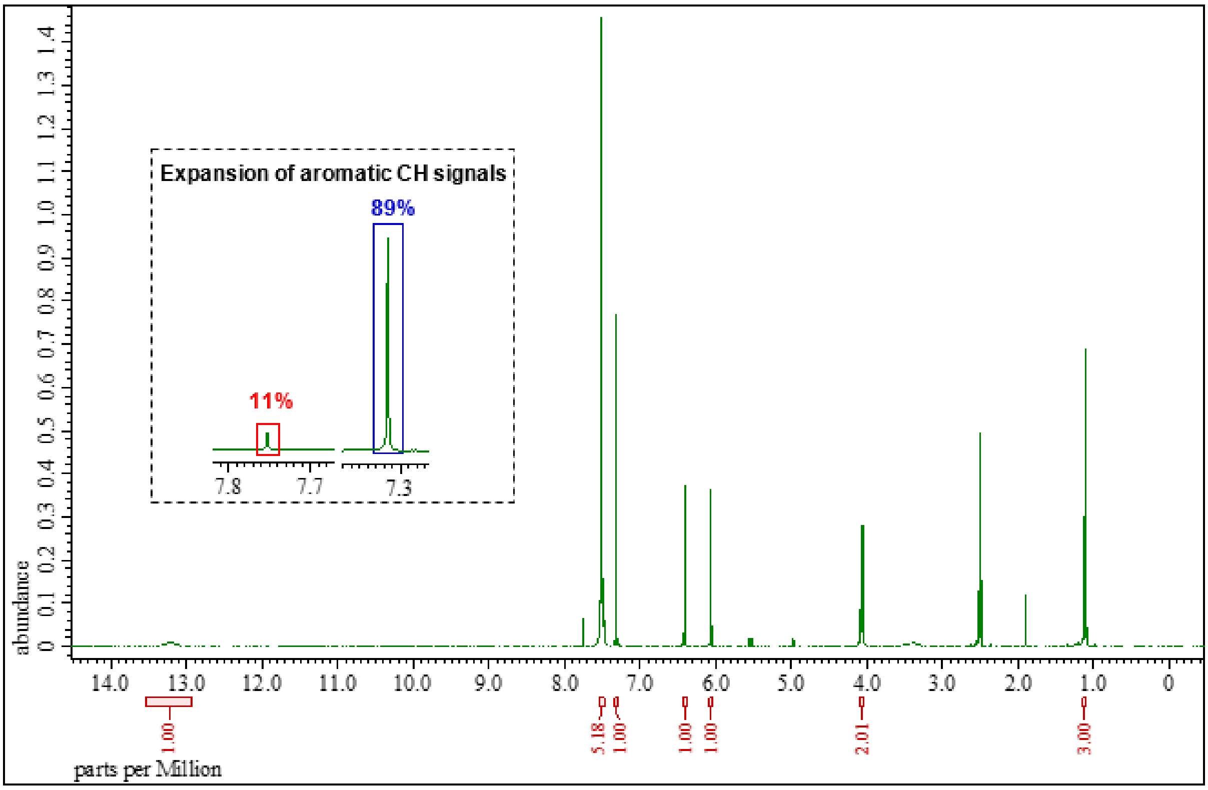
Expansion of aromatic CH signals
89%
11%
abundance
parts per Million
5.18
1.00
1.00
1.00
2.01
3.00
1.00

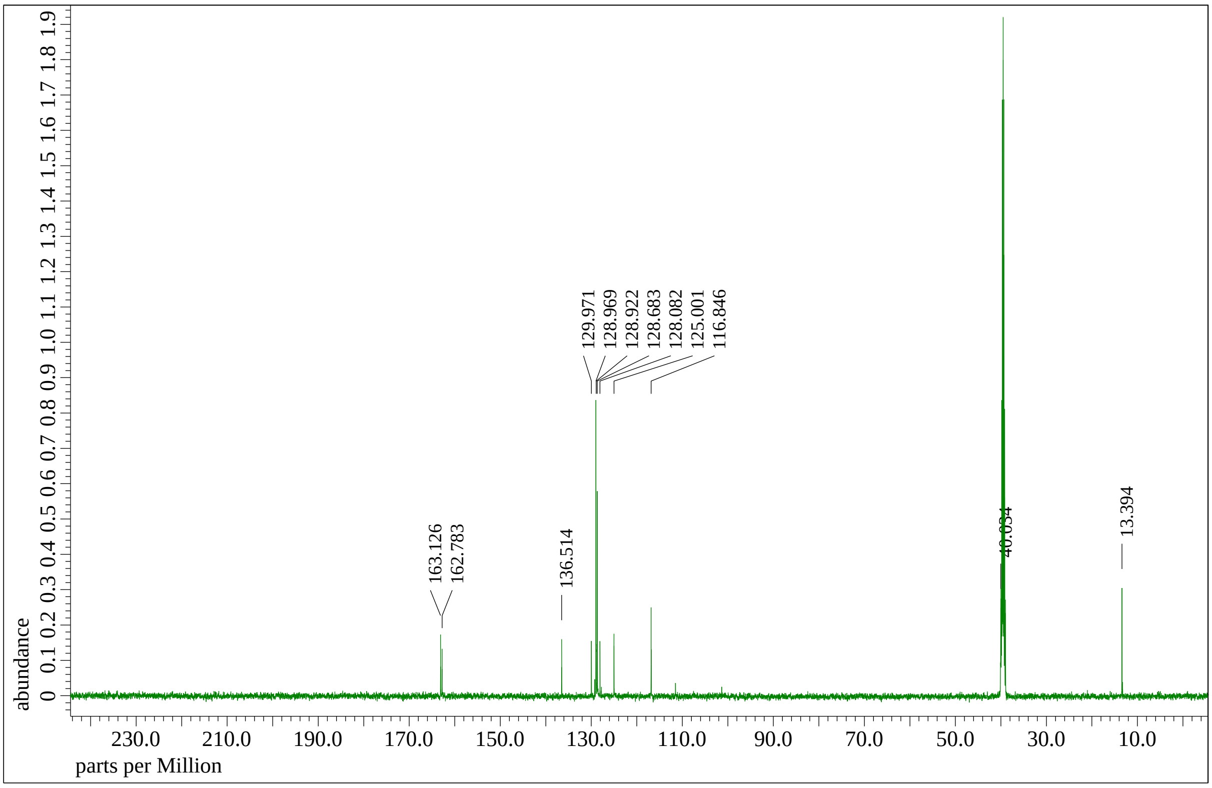
129.971
128.969
128.922
128.683
128.082
125.001
116.846
163.126
162.783
136.514
13.394
abundance
parts per Million

(3*R*)-7-benzyl-6-(*p*-tolyl)-2,3-dihyhydroimidazo[2,1-*b*]thiazol-7-ium-3-carboxylate 8{*2,2*}

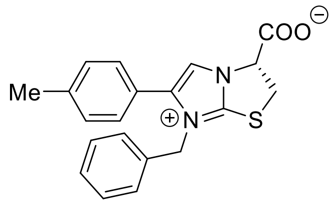

NMR: Mixture with 3% of **9**{*2,2*}. Creme amorphous solid, 23.3 mg (38%, 0.067 mmol). Cleaved from 308.4 mg of resin **2**{*2,2*} (0.565 mmol/g, 0.174 mmol of substrate). HPLC purity 99%. ^{1}H NMR (500 MHz, DMSO-d_6): δ = 7.91 (s, 1H), 7.31-7.38 (m, 7H), 7.12-7.14 (m, 2H), 5.28 (s, 2H), 5.18 (dd, *J* = 8.7, 5.5 Hz, 1H), 4.36 (dd, *J* = 11.3, 8.7 Hz, 1H), 4.22 (dd, *J* = 11.3, 5.5 Hz, 1H), 2.35 (s, 3H). ^{13}C NMR (126 MHz, DMSO-d_6): δ = 166.70, 150.33, 139.80, 136.97, 133.11, 129.69, 129.10, 128.93, 128.66, 127.71, 123.16, 118.90, 63.30, 50.15, 41.35, 20.84. HRMS (ESI-TOF, pos.): *m/z* calcd for $C_{20}H_{19}N_2O_2S$ [M+H]$^+$ 351.1162, found 351.1165.

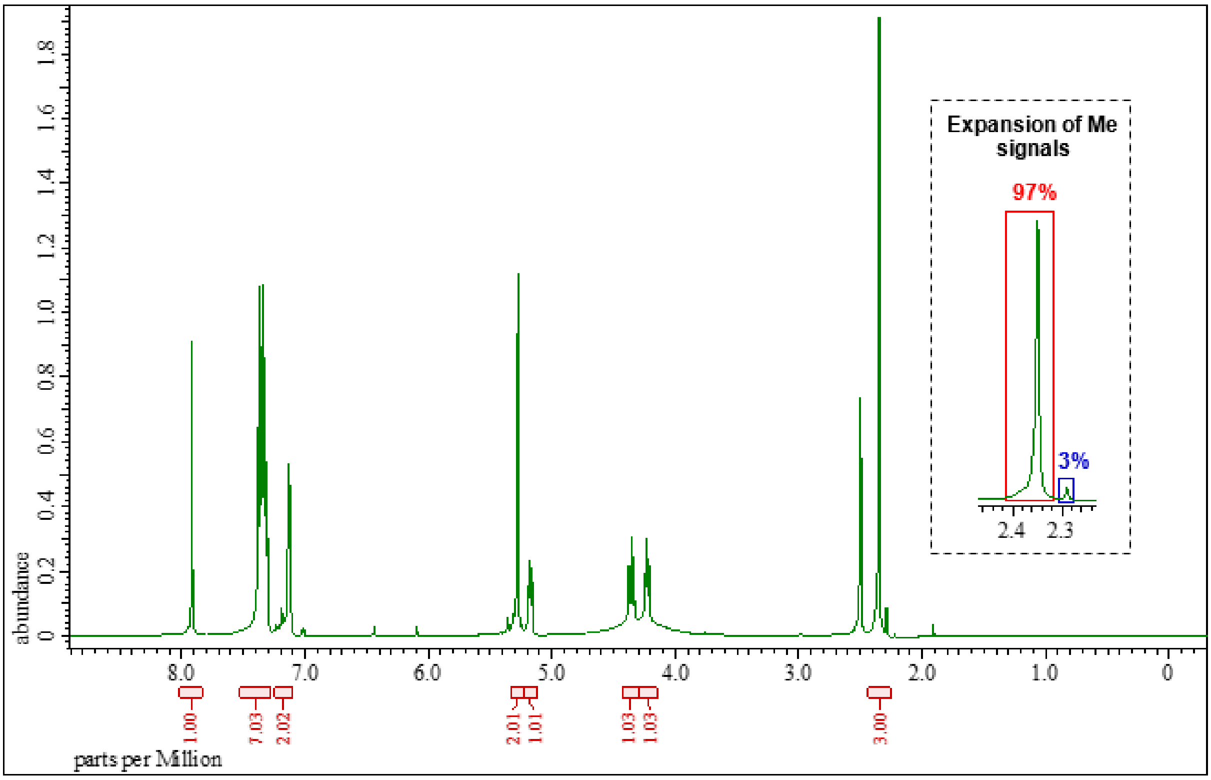

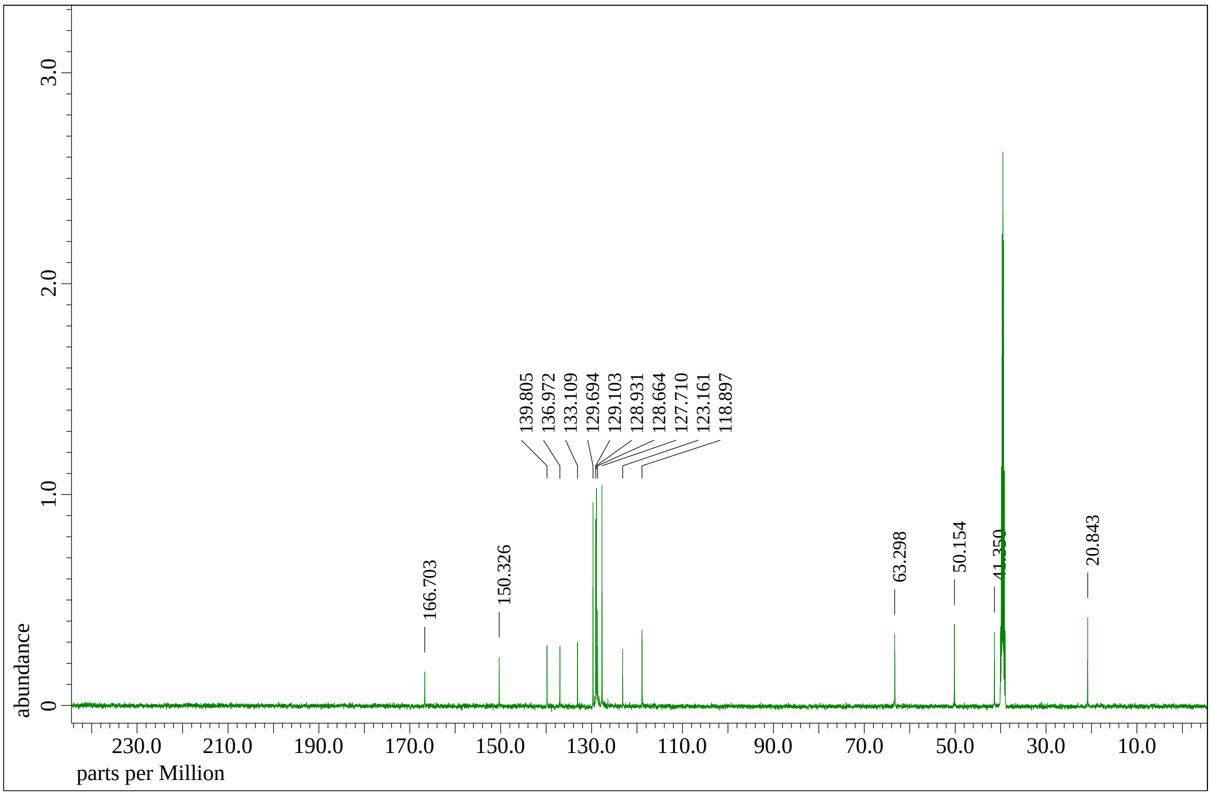

2-(3-benzyl-2-thioxo-6-(*p*-tolyl)-2,3-dihydro-1*H*-imidazol-1-yl)acrylic acid 9{*2,2*}

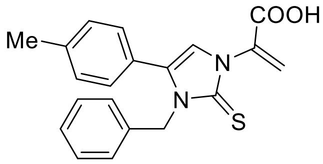

NMR: Mixture with 3% of **8**{*2,2*}. ^{1}H NMR (500 MHz, DMSO-d_6): δ = 7.35 (s, 1H), 7.15-7.26 (m, 7H), 7.01 (d, J = 7.2 Hz, 2H), 6.46 (d, J = 0.7 Hz, 1H), 6.12 (d, J = 0.7 Hz, 1H), 5.35 (s, 2H), 2.29 (s, 3H). ^{13}C NMR (126 MHz, DMSO-d_6): δ = 164.30, 163.15, 138.53, 136.71, 136.58, 130.51, 129.33, 128.42, 128.35, 127.14, 126.45, 125.24, 125.04, 116.90, 47.96, 20.76. HRMS (ESI-TOF, pos.): *m/z* calcd for $C_{20}H_{19}N_2O_2S$ $[M+H]^+$ 351.1162, found 351.1165.

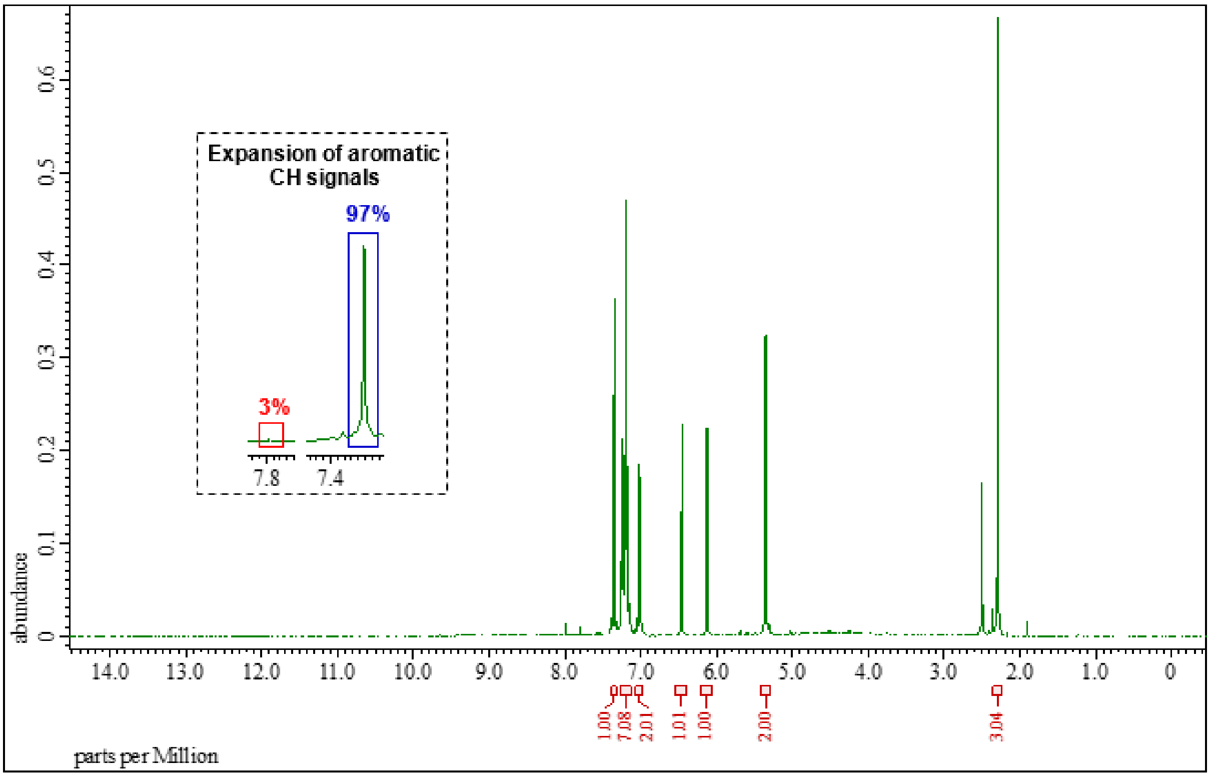
Expansion of aromatic CH signals
97%
3%
7.8
7.4
abundance
parts per Million
1.00
7.08
2.01
1.01
1.00
2.00
3.04

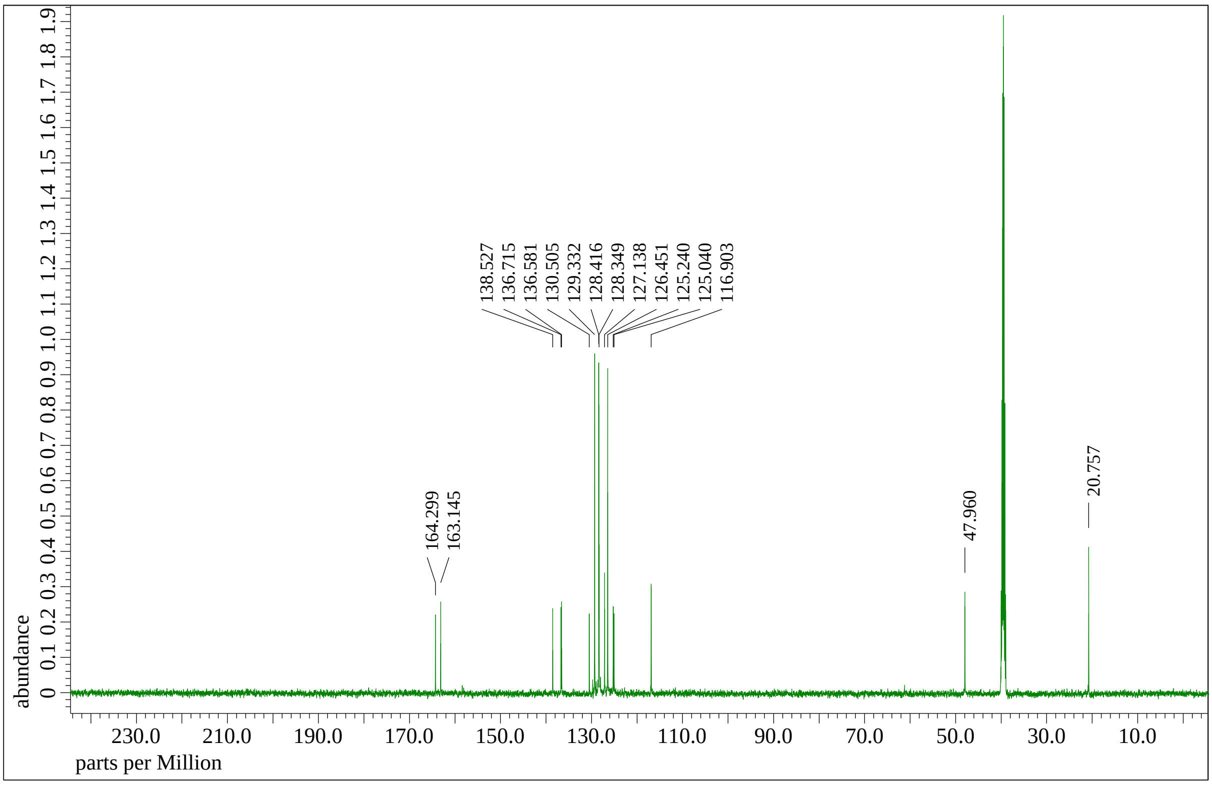
138.527
136.715
136.581
130.505
129.332
128.416
128.349
127.138
126.451
125.240
125.040
116.903
164.299
163.145
47.960
20.757
abundance
parts per Million

(3*R*)-7-(cyclohexylmethyl)-6-(*p*-tolyl)-2,3-dihyhydroimidazo[2,1-*b*]thiazol-7-ium-3-carboxylate 8{*2,5*}

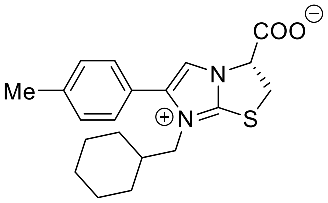

Creme amorphous solid, 13.3 mg (49%, 0.038 mmol). Cleaved from 135.7 mg of resin **2**{*2,5*} (0.565 mmol/g, 0.077 mmol of substrate). HPLC purity 99%. ^{1}H NMR (500 MHz, DMSO-d_6): δ = 7.71 (s, 1H), 7.41 (br. d, J = 8.0 Hz, 2H), 7.35 (br. d, J = 8.0 Hz, 2H), 5.00 (dd, J = 8.6, 5.4 Hz, 1H), 4.36 (dd, J = 11.1, 8.6 Hz, 1H), 4.25 (q, J = 11.1, 5.4 Hz, 1H), 3.85 (d, J = 7.2 Hz, 2H), 2.36 (s, 3H), 1.37-1.54 (m, 6H), 0.96-1.00 (m, 3H), 0.69-0.78 (m, 2H). ^{13}C NMR (126 MHz, DMSO-d_6): δ = 167.26, 150.22, 140.27, 137.29, 130.30, 129.60, 124.12, 119.33, 64.55, 53.29, 41.91, 37.37, 29.95, 25.87, 25.33, 21.40. HRMS (ESI-TOF, pos.): *m/z* calcd for $C_{20}H_{25}N_2O_2S$ [M+H]$^+$ 357.1631, found 357.1630.

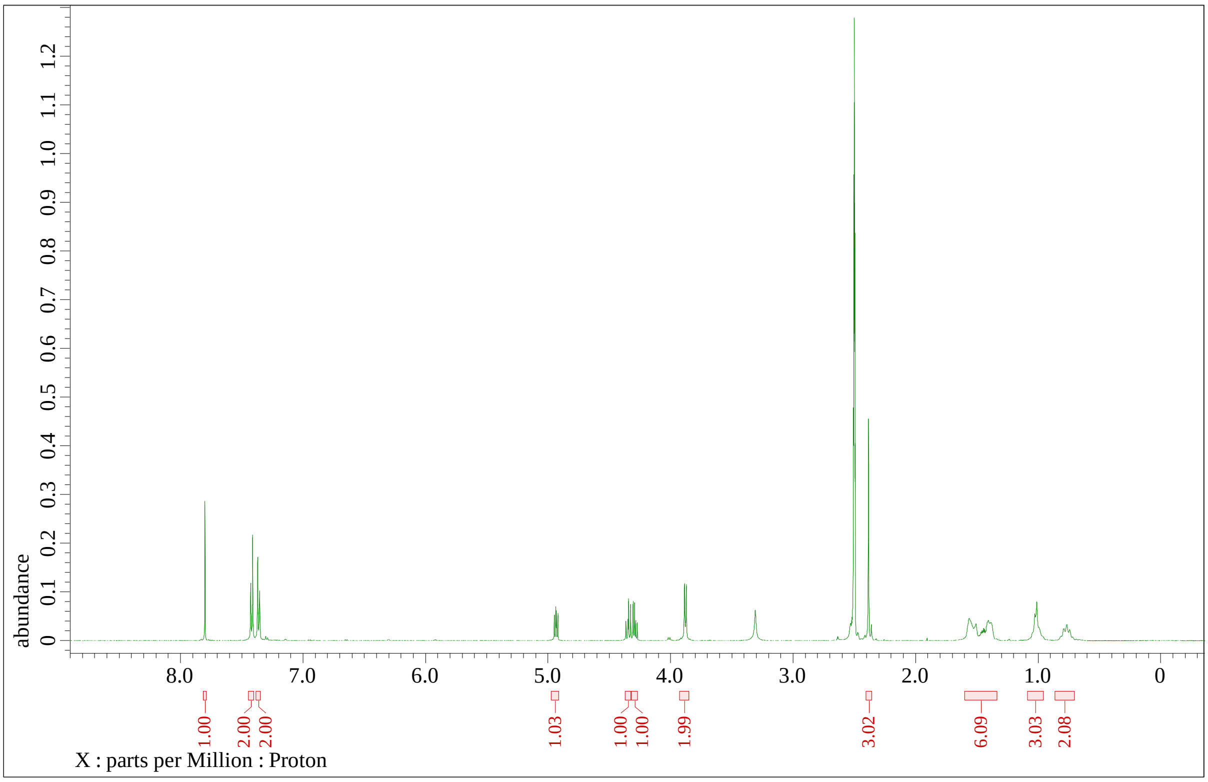

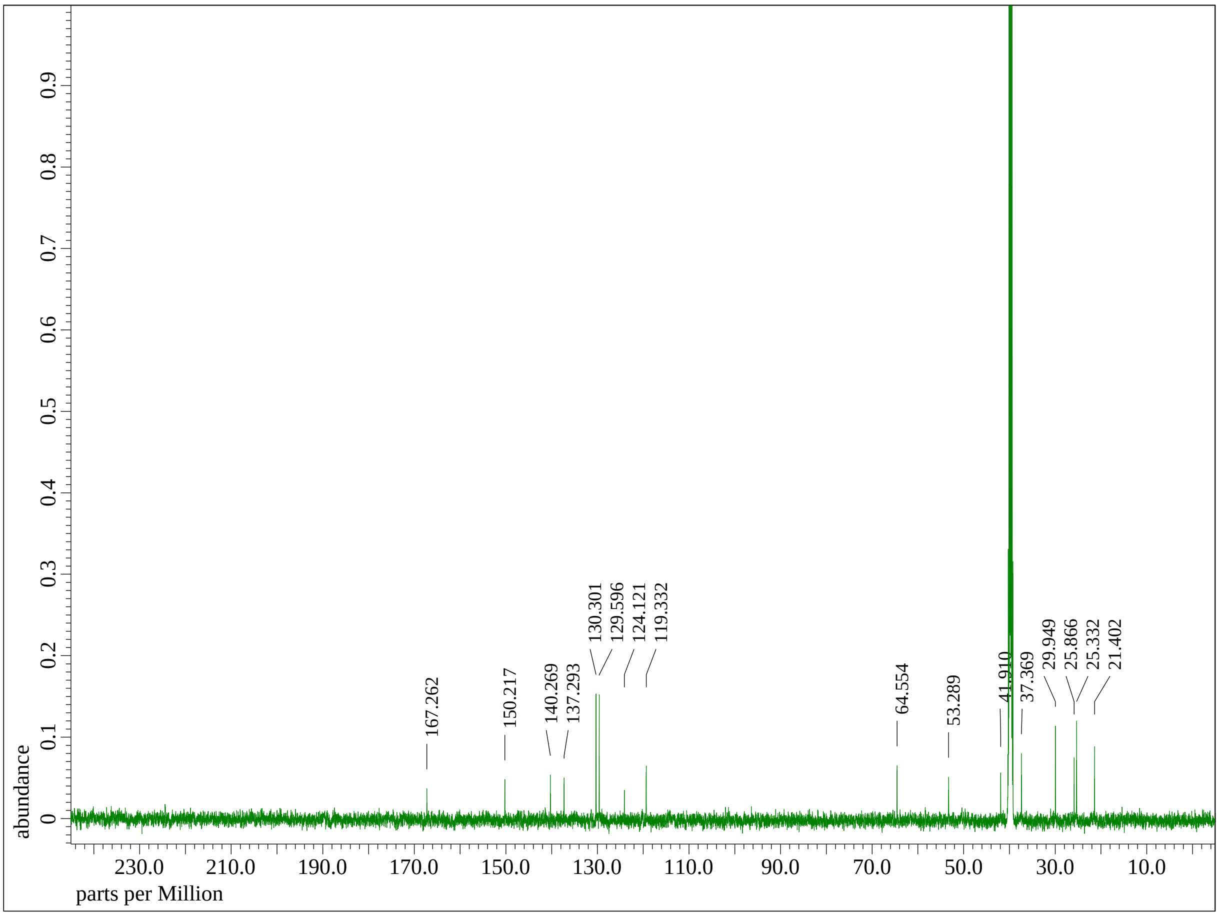

(3*R*)-7-allyl-6-(4-methoxyphenyl)-2,3-dihyhydroimidazo[2,1-*b*]thiazol-7-ium-3-carboxylate 8{*3,3*}

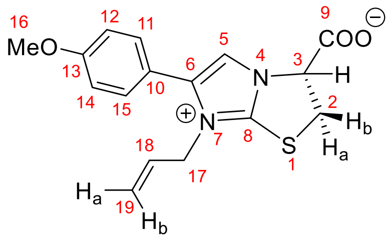

NMR: Mixture with 16% of **9**{*3,3*}. Creme amorphous solid, yield 25.3 mg (32%, 0.080 mmol) of which **8** 22.3 mg (28%, 0.071 mmol) and **9** 3.0 mg (4%, 0.009 mmol). Cleaved from 443.8 mg of resin **2**{*3,3*} (0.575 mmol/g, 0.255 mmol of substrate). HPLC purity 99%. ^{1}H NMR (500 MHz, DMSO-d_6): δ = 7.85 (s, 1H, HC^5), 7.43 (br. d, J = 8.8 Hz, 2H, $HC^{11,15}$), 7.09 (br. d, J = 8.8 Hz, 2H, $HC^{12,14}$), 5.86-5.94 (m, 1H, HC^{18}), 5.32 (br. d, J = 10.4 Hz, 1H, H_bC^{19}), 5.15 (dd, J = 8.7, 5.4 Hz, 1H, HC^3), 5.12 (br. d, J = 17.2 Hz, 1H, H_aC^{19}), 4.61 (ddd, J = 5.0, 1.8, 1.8 Hz, 2H, HC^{17}), 4.39 (dd, J = 11.2, 8.7 Hz, 1H, H_bC^2), 4.27 (dd, J = 11.2, 5.4 Hz, 1H, H_aC^2), 3.82 (s, 3H, HC^{16}). ^{13}C NMR (126 MHz, DMSO-d_6): δ = 167.19 (C9), 161.01 (C13), 150.37 (C8), 137.31 (C6), 131.19 (C11,15), 130.80 (C18), 120.10 (C5), 118.91 (C19), 118.65 (C10), 115.17 (C12,14), 64.03 (C3), 55.92 (C16), 49.45 (C17), 41.99 (C2). HRMS (ESI-TOF, pos.): *m/z* calcd for $C_{16}H_{17}N_2O_3S$ $[M+H]^+$ 317.0954, found 317.0957.

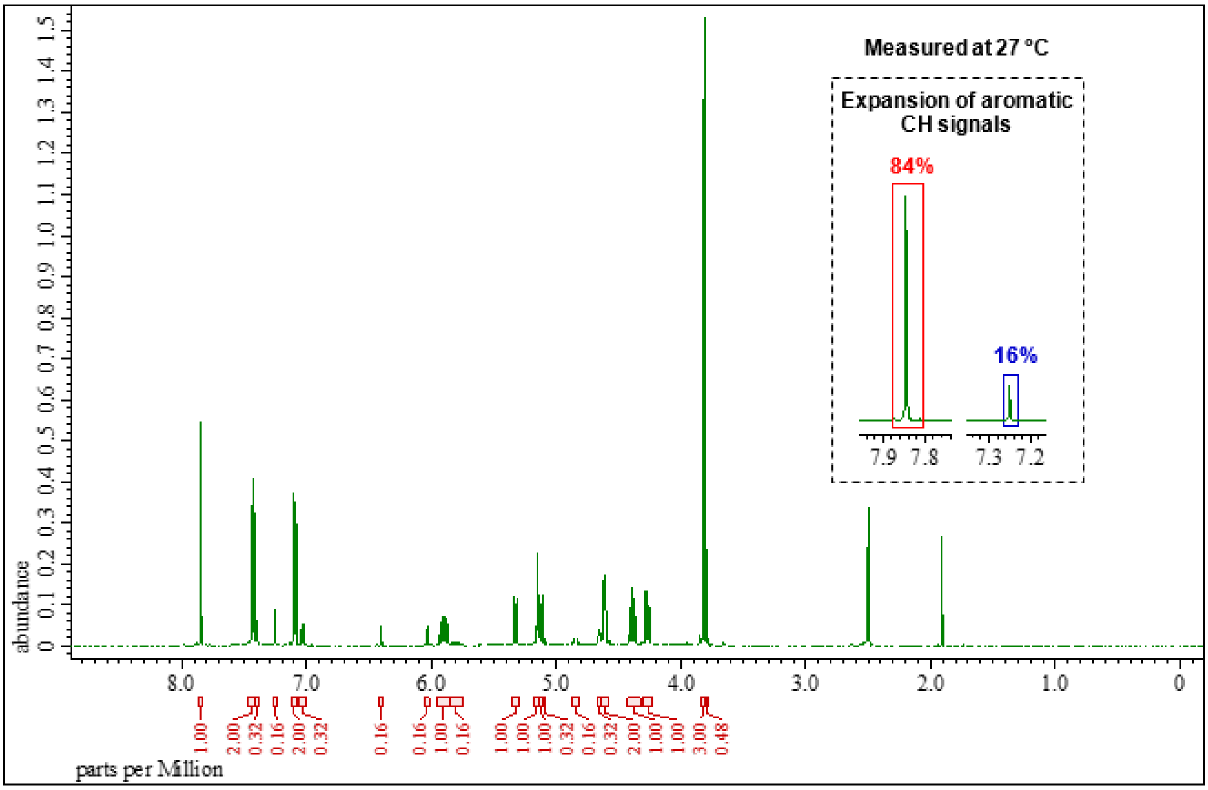

Measured at 27 °C
Expansion of aromatic CH signals
84%
16%
7.9 7.8
7.3 7.2
abundance
parts per Million
8.0 7.0 6.0 5.0 4.0 3.0 2.0 1.0 0
1.00 2.00 0.32 0.16 2.00 0.32 0.16 0.16 1.00 0.16 1.00 1.00 1.00 0.32 0.16 0.32 2.00 1.00 1.00 3.00 0.48

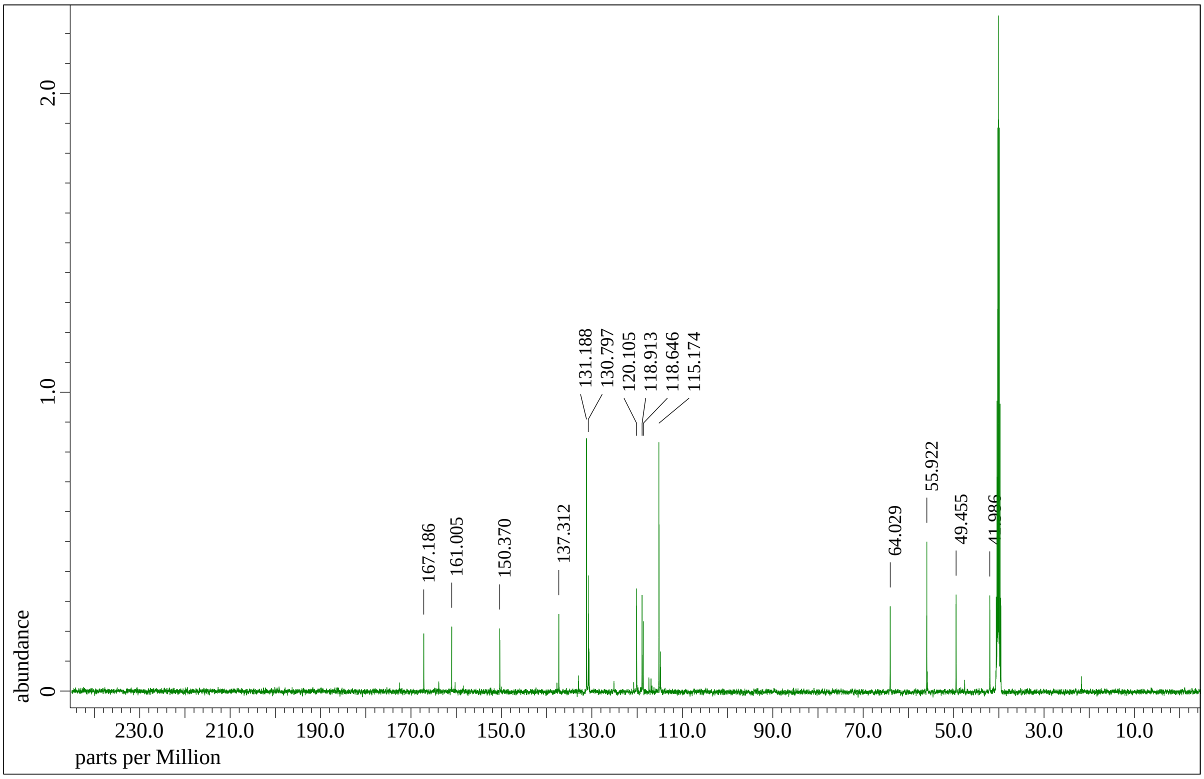

abundance
parts per Million
167.186
161.005
150.370
137.312
131.188
130.797
120.105
118.913
118.646
115.174
64.029
55.922
49.455
41.986
230.0 210.0 190.0 170.0 150.0 130.0 110.0 90.0 70.0 50.0 30.0 10.0

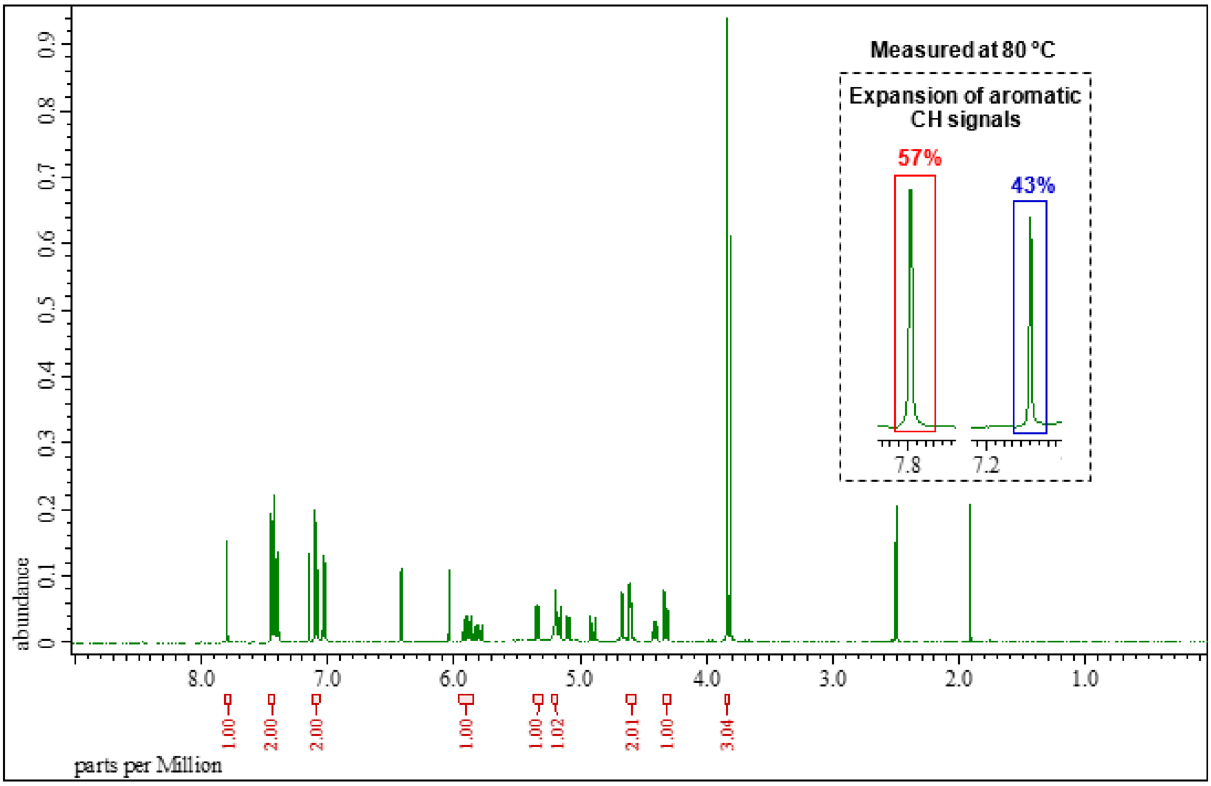
Measured at 80 °C
Expansion of aromatic CH signals
57%
43%
7.8
7.2
abundance
parts per Million
1.00
2.00
2.00
1.00
1.00
1.02
2.01
1.00
3.04

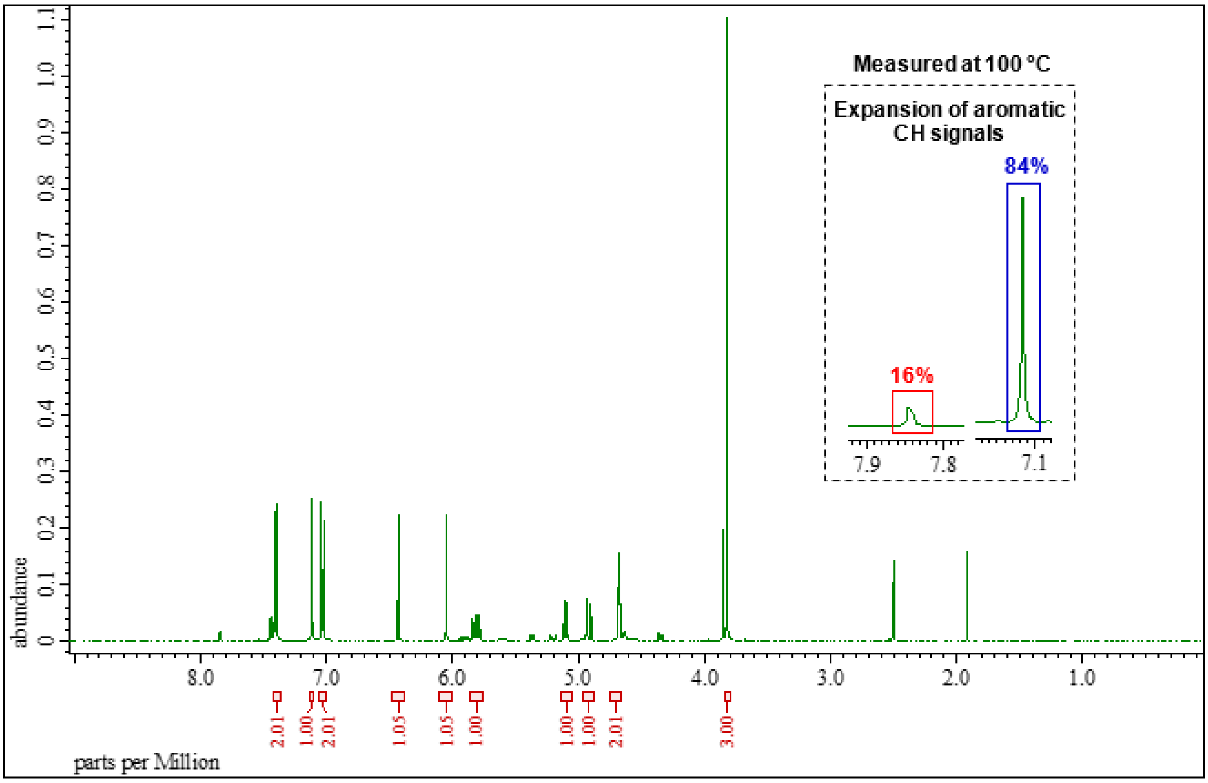
Measured at 100 °C
Expansion of aromatic CH signals
84%
16%
7.9
7.8
7.1
abundance
parts per Million
2.01
1.00
2.01
1.05
1.05
1.00
1.00
1.00
2.01
3.00

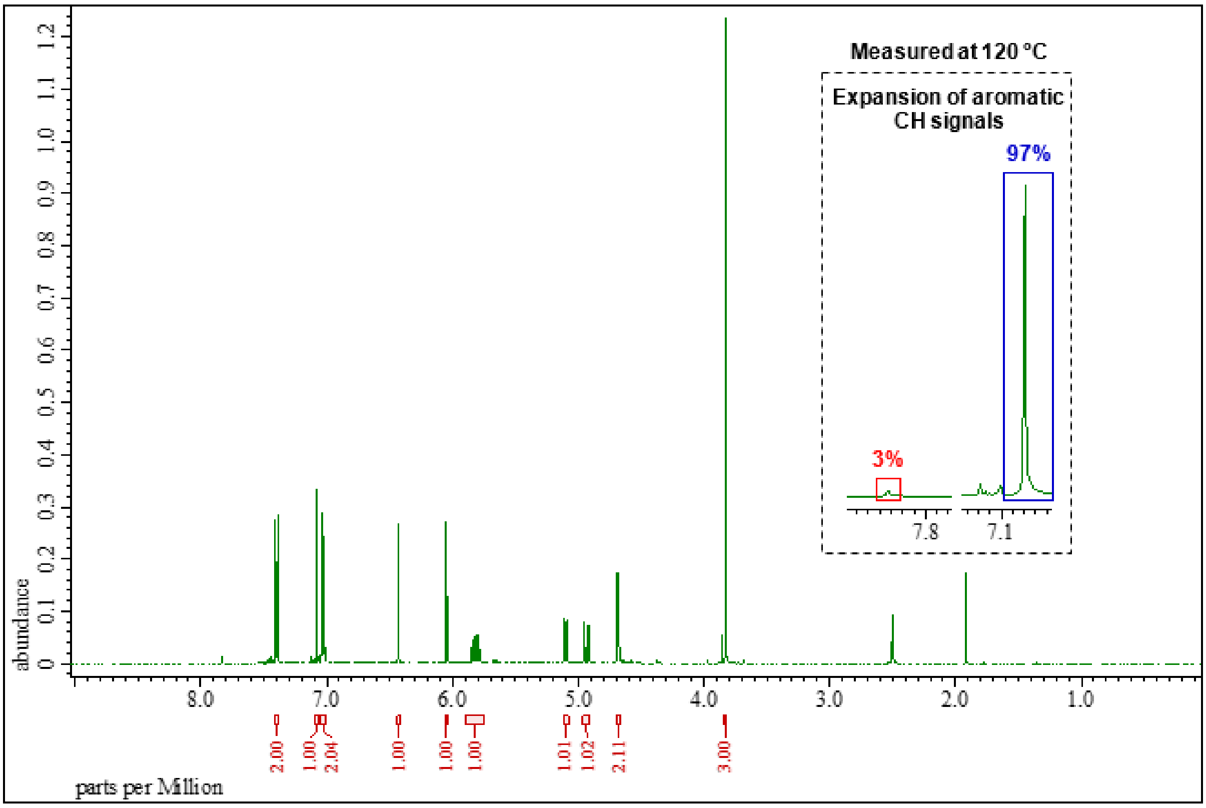
Measured at 120 °C
Expansion of aromatic CH signals
97%
3%
7.8
7.1
abundance
parts per Million
2.00
1.00
2.04
1.00
1.00
1.00
1.01
1.02
2.11
3.00

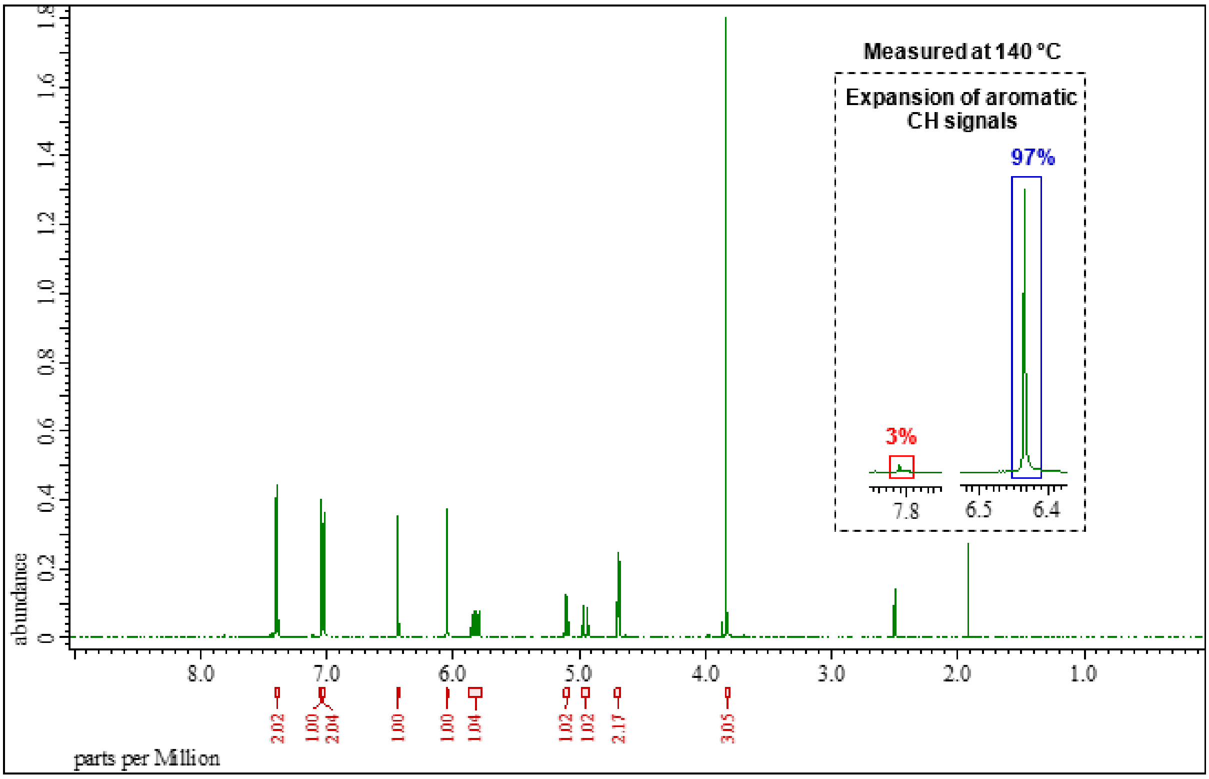
Measured at 140 °C
Expansion of aromatic CH signals
97%
3%
7.8
6.5
6.4
abundance
parts per Million
2.02
1.00
2.04
1.00
1.00
1.04
1.02
1.02
2.17
3.05

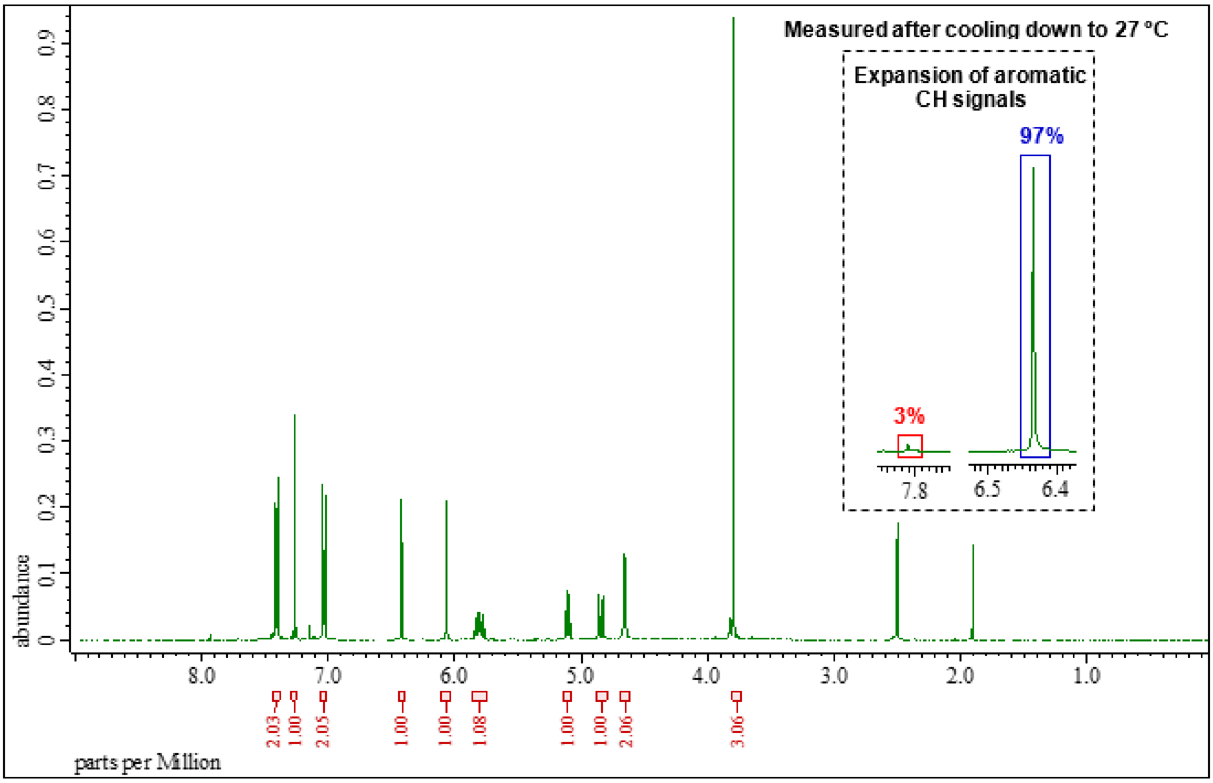
Measured after cooling down to 27 °C
Expansion of aromatic CH signals
97%
3%
7.8
6.5
6.4
abundance
0
0.1
0.2
0.3
0.4
0.5
0.6
0.7
0.8
0.9
8.0
7.0
6.0
5.0
4.0
3.0
2.0
1.0
2.03
1.00
2.05
1.00
1.00
1.08
1.00
1.00
2.06
3.06
parts per Million

2-(3-allyl-4-(4-methoxyphenyl)-2-thioxo-2,3-dihydro-1*H*-imidazol-1-yl)acrylic acid 9{*3,3*}

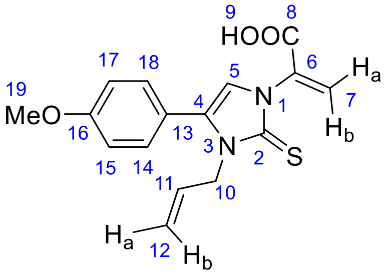

NMR: Mixture with 3% of **8**{*3,3*}. Creme amorphous solid, 25.3 mg (32%, 0.080 mmol) of which **8**{*3,3*} 0.8 mg (1%, 0.003 mmol) and **9**{*3,3*} 24.5 mg (31%, 0.078 mmol). ^{1}H NMR (500 MHz, DMSO-d_6): δ = 12.93 (br. s, 1H, HO^9), 7.40 (br. d, J = 8.8 Hz, 2H, HC^{14,18}), 7.26 (s, 1H, HC^5), 7.03 (br. d, J = 8.8 Hz, 2H, HC^{15,17}), 6.42 (d, J = 0.7 Hz, 1H, H$_a$C^7), 6.06 (d, J = 0.7 Hz, 1H, H$_b$C^7), 5.76-5.85 (m, 1H, HC^{11}), 5.10 (br. d, J = 10.4 Hz, 1H, H$_b$C^{12}), 4.84 (br. d, J = 17.2 Hz, 1H, H$_a$C^{12}), 4.65 (ddd, J = 4.8, 1.6, 1.6 Hz, 2H, HC^{10}), 3.79 (s, 3H, HC^{19}). ^{13}C NMR (126 MHz, DMSO-d_6): δ = 163.36 (C2), 163.15 (C8), 159.73 (C16), 136.67 (C6), 132.36 (C11), 130.25 (C4), 130.12 (C14,18), 125.04 (C7), 120.13 (C13), 116.86 (C12), 116.29 (C5), 114.26 (C15,17), 55.24 (C19), 47.00 (C10). ^{15}N NMR (51 MHz, DMSO-d_6): δ = 172.4 (N1), 166.6 (N3). HRMS (ESI-TOF, pos.): m/z calcd for $C_{16}H_{17}N_2O_3S$ [M+H]$^+$ 317.0954, found 317.0957.

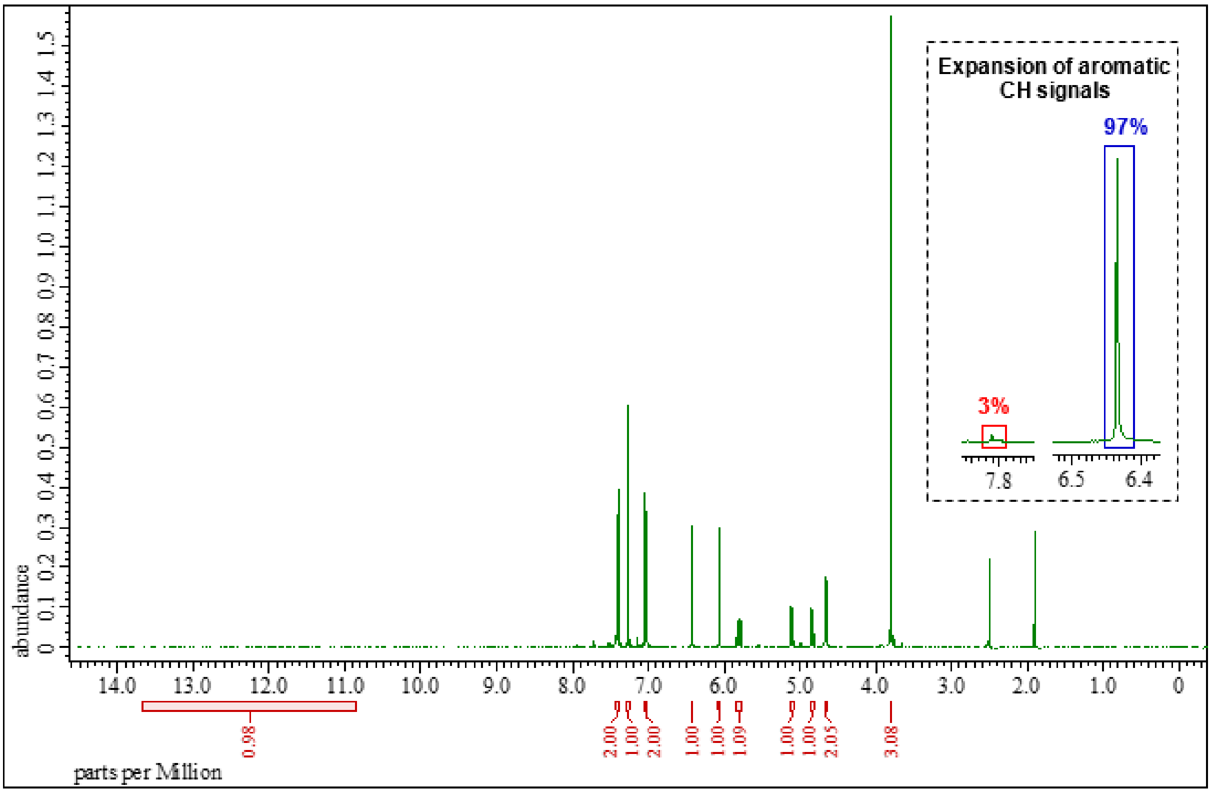

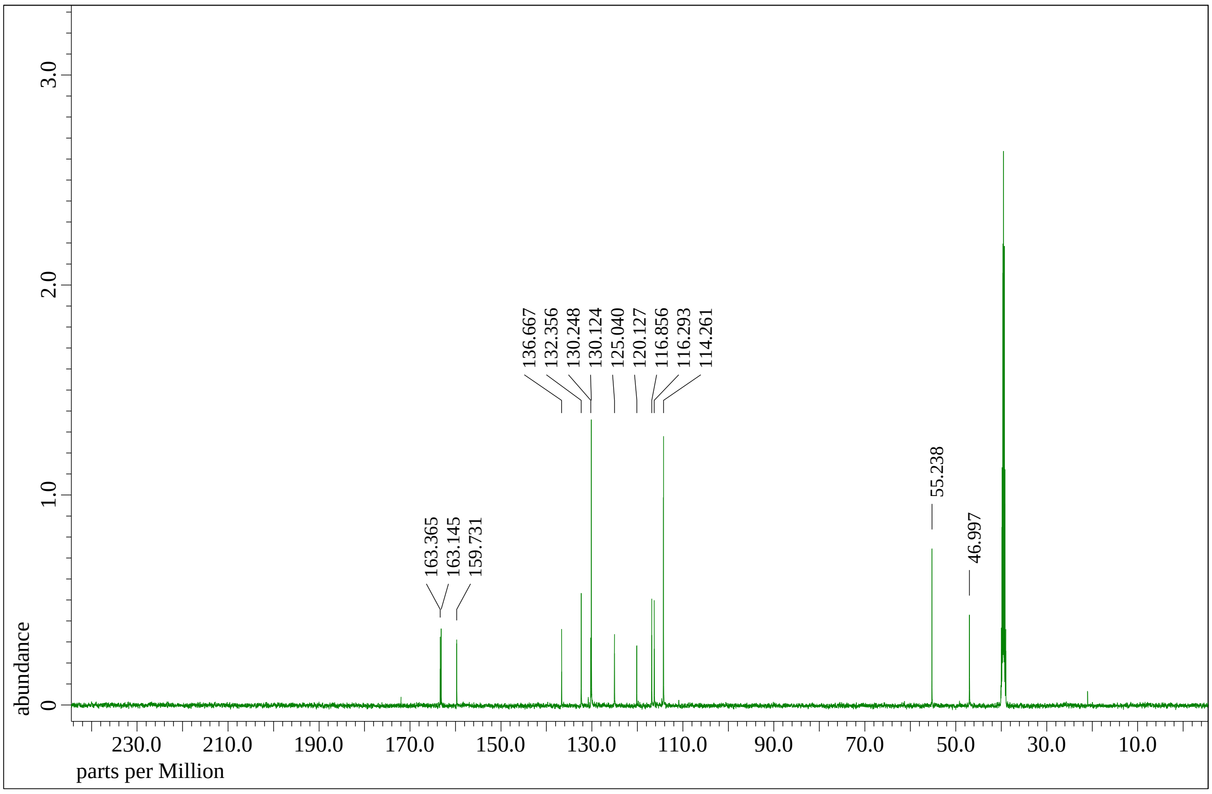

abundance
parts per Million
163.365
163.145
159.731
136.667
132.356
130.248
130.124
125.040
120.127
116.856
116.293
114.261
55.238
46.997

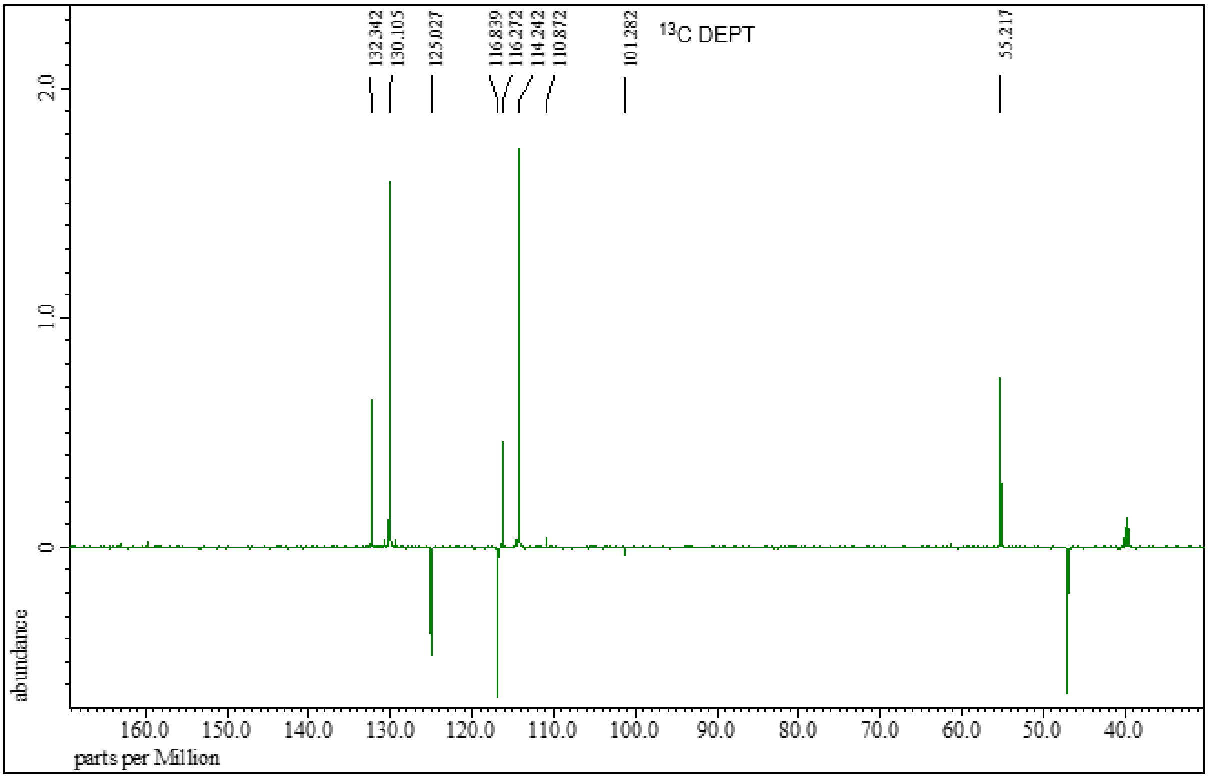

^{13}C DEPT
abundance
parts per Million
132.342
130.105
125.027
116.839
116.272
114.242
110.872
101.282
55.217

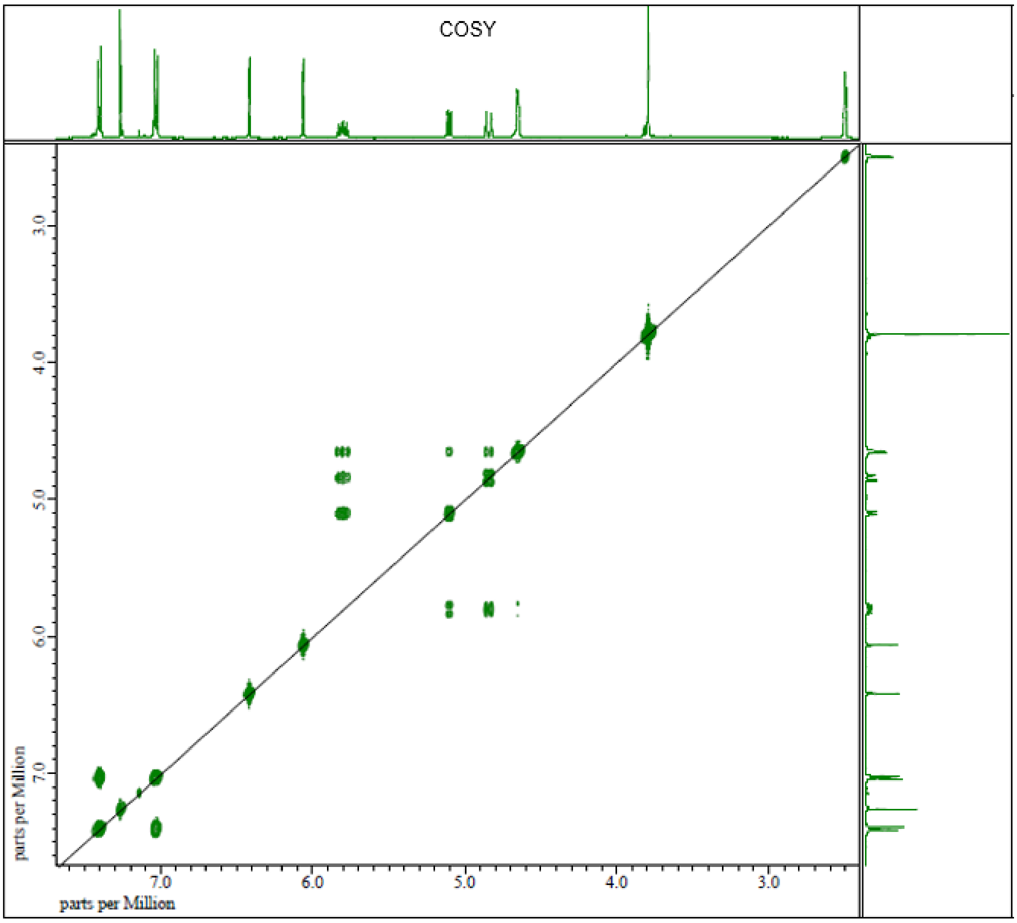
COSY
parts per Million
3.0
4.0
5.0
6.0
7.0
parts per Million
7.0
6.0
5.0
4.0
3.0

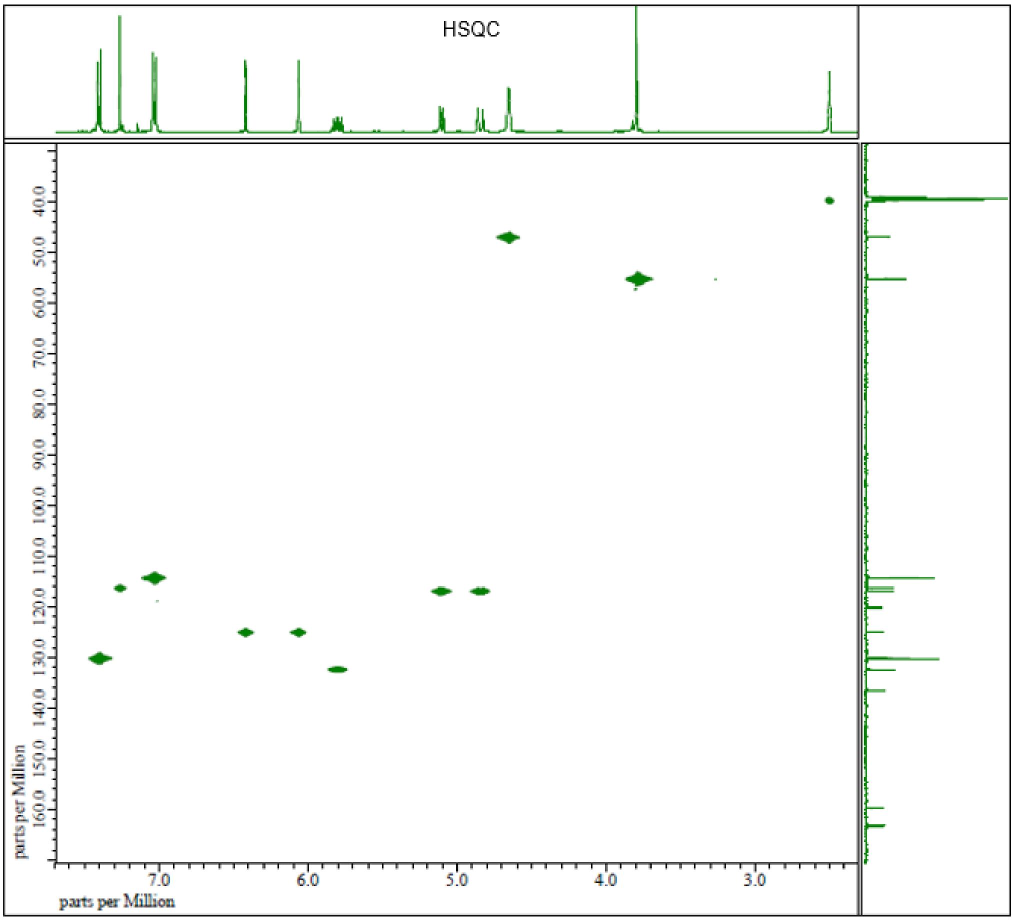
HSQC
parts per Million
40.0
50.0
60.0
70.0
80.0
90.0
100.0
110.0
120.0
130.0
140.0
150.0
160.0
parts per Million
7.0
6.0
5.0
4.0
3.0

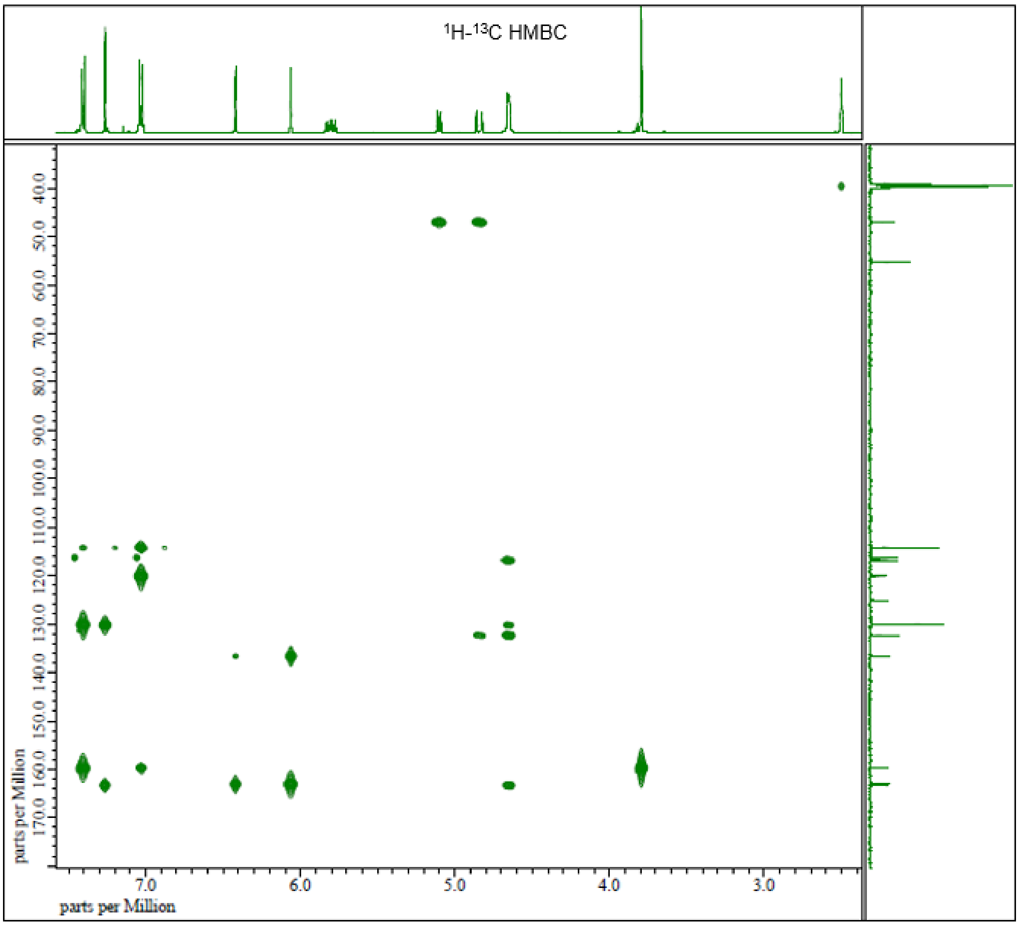

1H-13C HMBC
parts per Million
40.0
50.0
60.0
70.0
80.0
90.0
100.0
110.0
120.0
130.0
140.0
150.0
160.0
170.0
7.0
6.0
5.0
4.0
3.0
parts per Million

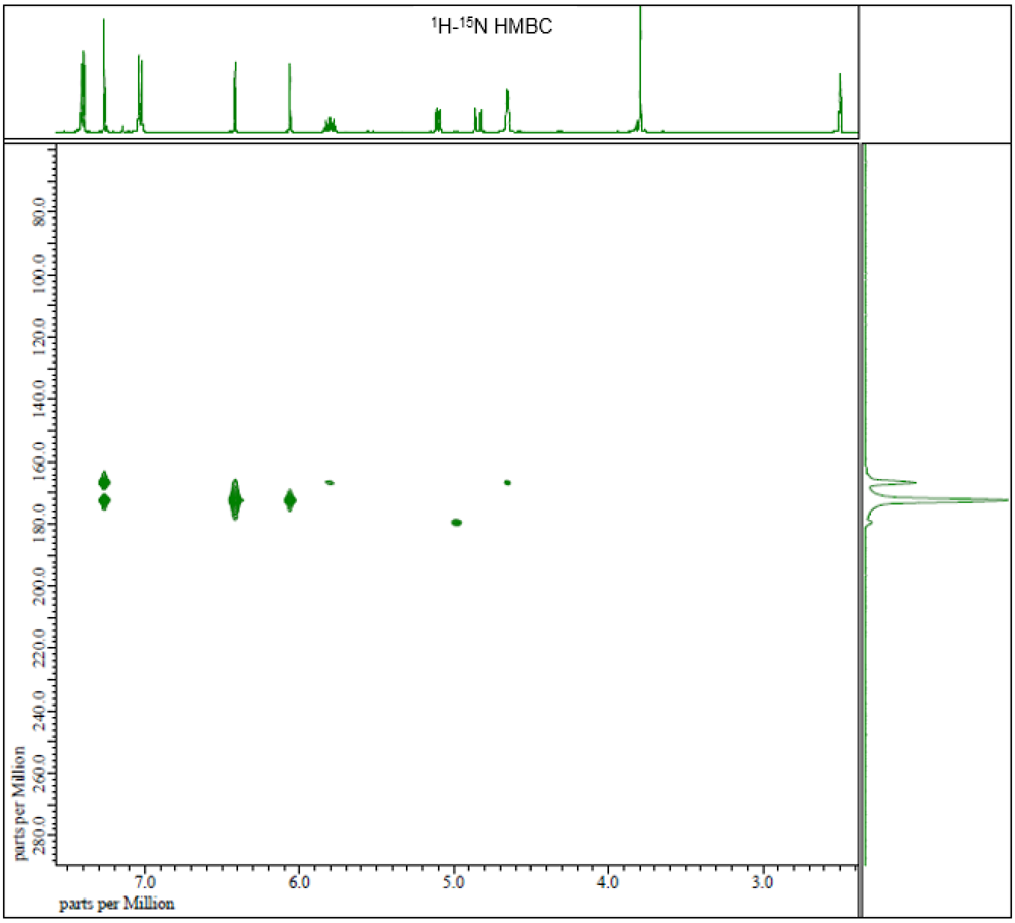

1H-15N HMBC
parts per Million
80.0
100.0
120.0
140.0
160.0
180.0
200.0
220.0
240.0
260.0
280.0
7.0
6.0
5.0
4.0
3.0
parts per Million

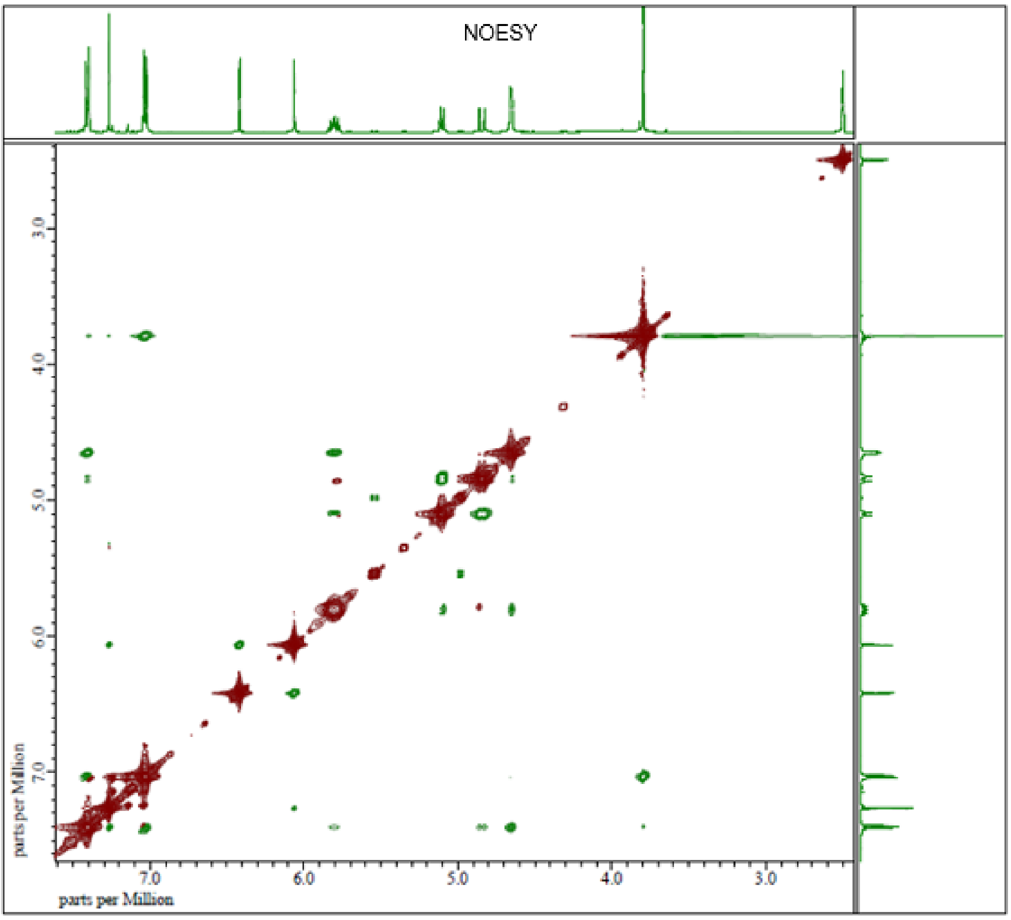

(3*R*)-7-ethyl-6-(4-methoxyphenyl)-2,3-dihyhydroimidazo[2,1-*b*]thiazol-7-ium-3-carboxylate 8{*3,4*}

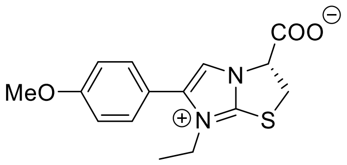

Creme amorphous solid, 19.3 mg (59%, 0.063 mmol). Cleaved from 186.1 mg of resin **2**{*3,4*} (0.575 mmol/g, 0.107 mmol of substrate). HPLC purity 99%. ^{1}H NMR (500 MHz, DMSO-*d*$_6$): δ = 7.76 (s, 1H), 7.46 (br. d, *J* = 8.8 Hz, 2H), 7.11 (br. d, *J* = 8.8 Hz, 2H), 4.93 (dd, *J* = 8.4, 6.4 Hz, 1H), 4.34 (dd, *J* = 11.1, 8.4 Hz, 1H), 4.30 (dd, *J* = 11.1, 6.4 Hz, 1H), 3.99 (q, *J* = 7.3 Hz, 2H), 3.82 (s, 3H), 1.19 (t, *J* = 7.3 Hz, 3H). ^{13}C NMR (126 MHz, DMSO-*d*$_6$): δ = 165.47, 160.33, 148.37, 136.04, 130.69, 118.59, 118.39, 114.60, 63.97, 55.32, 42.23, 41.48, 13.85. HRMS (ESI-TOF, pos.): *m/z* calcd for $C_{15}H_{17}N_2O_3S$ [M+H]$^+$ 305.0954, found 305.0953.

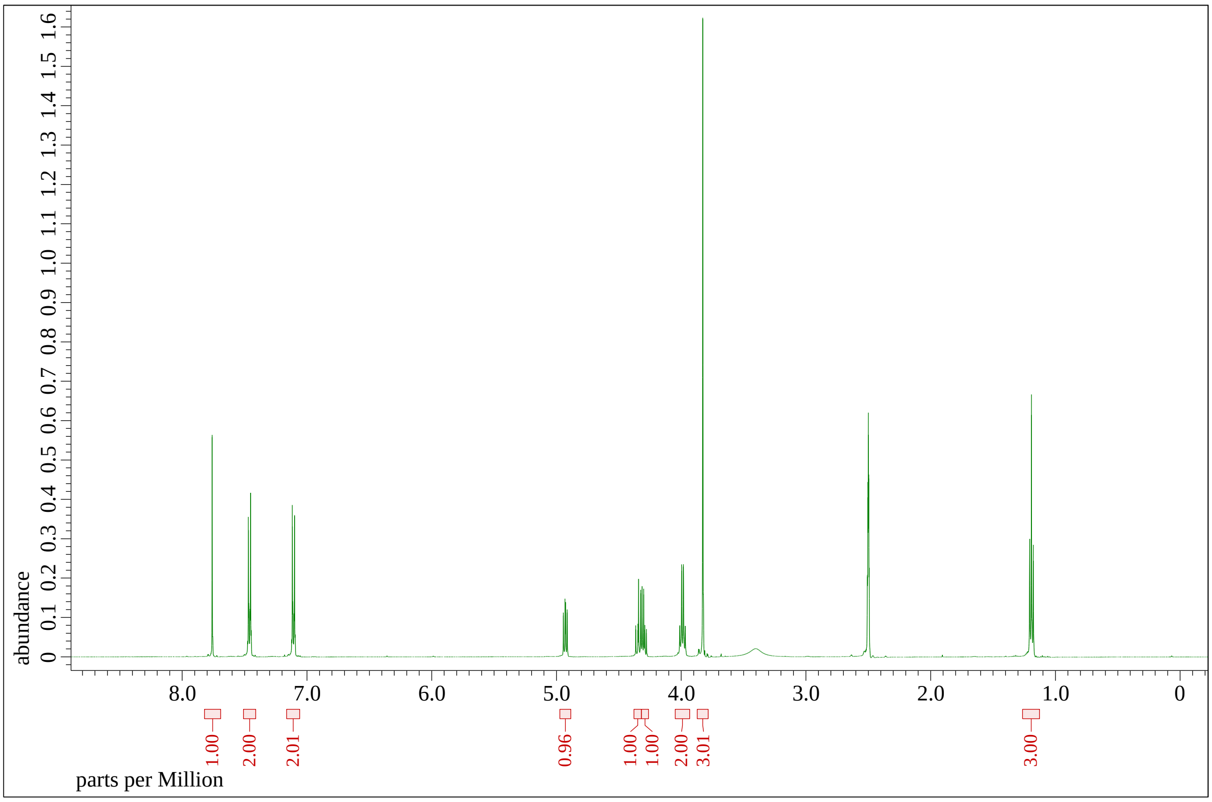

abundance
parts per Million
8.0
7.0
6.0
5.0
4.0
3.0
2.0
1.0
0
1.00
2.00
2.01
0.96
1.00
1.00
2.00
3.01
3.00

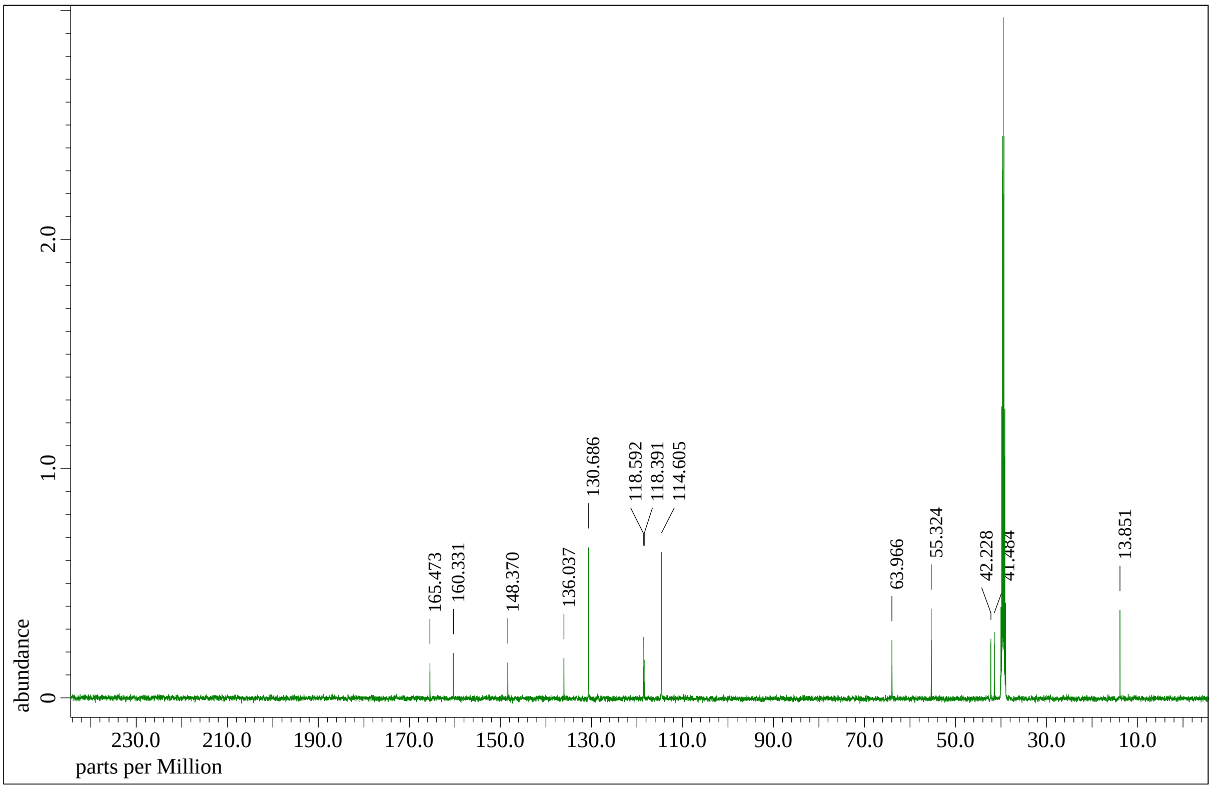

abundance
parts per Million
165.473
160.331
148.370
136.037
130.686
118.592
118.391
114.605
63.966
55.324
42.228
41.484
13.851
230.0
210.0
190.0
170.0
150.0
130.0
110.0
90.0
70.0
50.0
30.0
10.0

2-(3-allyl-4-(4-methoxyphenyl)-2-thioxo-2,3-dihydro-1*H*-imidazol-1-yl)acrylic acid 9{*3,4*}

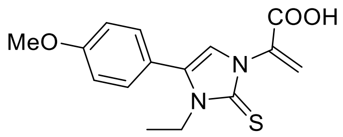

NMR: Mixture with 13% of **8**{*3,4*}. ^{1}H NMR (500 MHz, DMSO-d_6): δ = 13.24 (br. s, 1H), 7.43 (br. d, J = 8.8 Hz, 2H), 7.22 (s, 1H), 7.07 (br. d, J = 8.8 Hz, 2H), 6.40 (d, J = 0.7 Hz, 1H), 6.04 (d, J = 0.7 Hz, 1H), 4.02 (q, J = 7.1 Hz, 2H), 3.81 (s, 3H), 1.11 (t, J = 7.1 Hz, 3H). ^{13}C NMR (126 MHz, DMSO-d_6): δ = 163.16, 162.38, 159.75, 136.58, 130.51, 130.33, 129.81, 124.84, 120.17, 116.31, 114.38, 55.24, 13.40. HRMS (ESI-TOF, pos.): *m/z* calcd for $C_{15}H_{17}N_2O_3S$ $[M+H]^+$ 305.0954, found 305.0953.

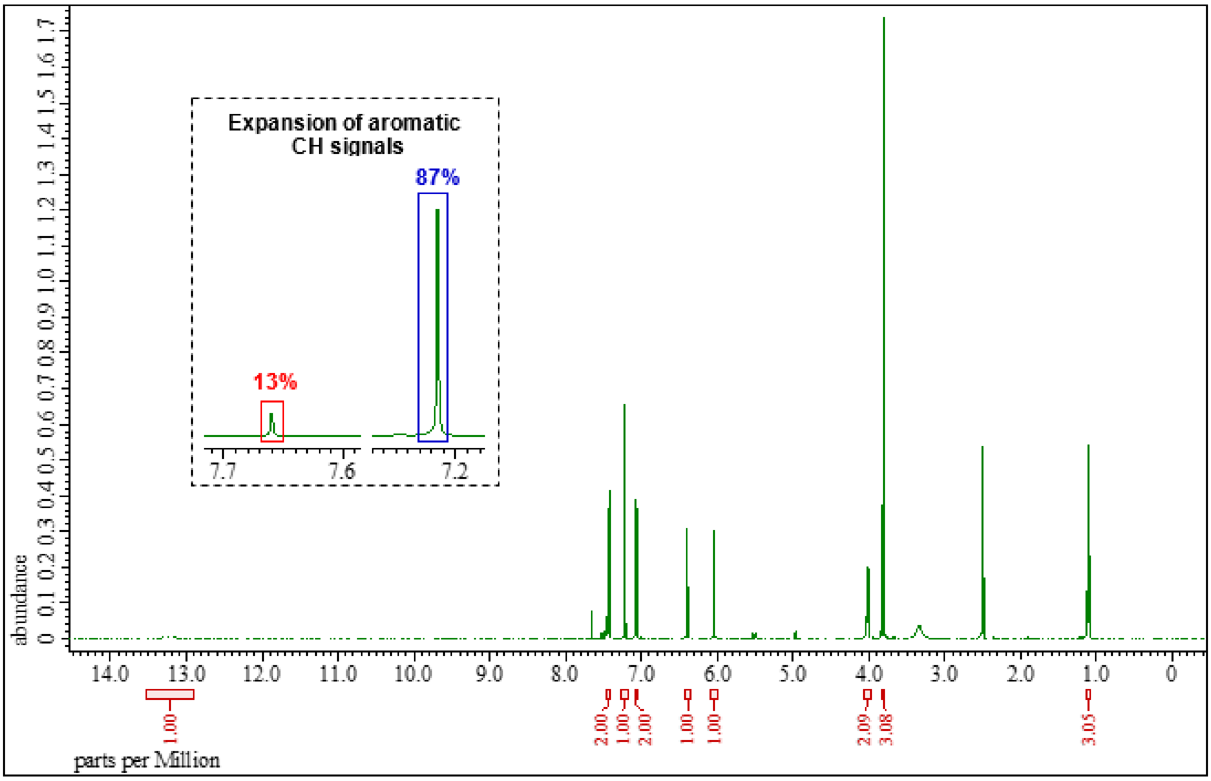

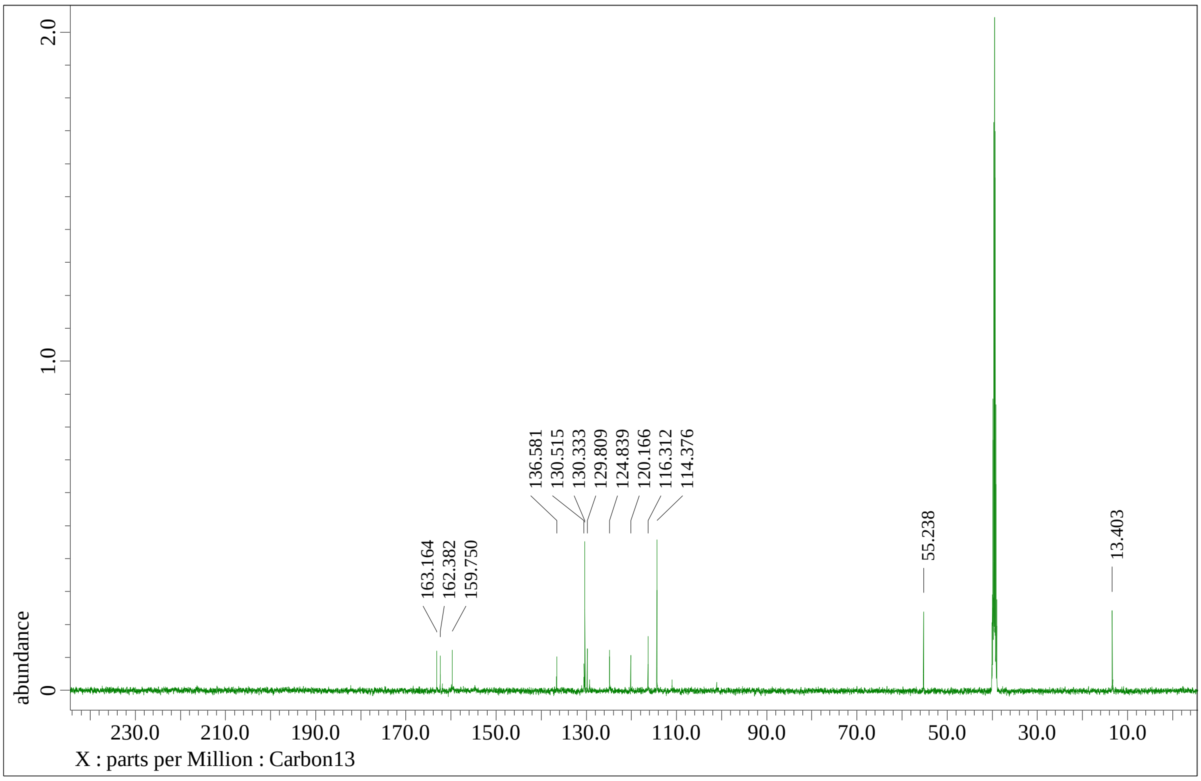

(3*R*)-6-(4-methoxyphenyl)-7-(2-morpholinoethyl)-2,3-dihyhydroimidazo[2,1-*b*]thiazol-7-ium-3-carboxylate 8{*3,6*}

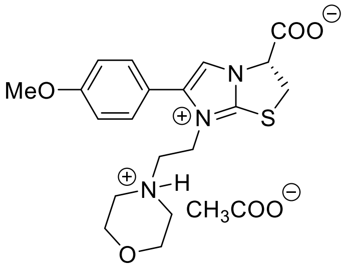

NMR: Mixture with 40% of **9**{*3,6*}. Creme amorphous solid, 18.6 mg (39%, 0.048 mmol) of which **8** 16.9 mg (36%, 0.043 mmol) and **9** 1.7 mg (3%, 0.004 mmol). Cleaved from 210.1 mg of resin **2**{*3,6*} (0.575 mmol/g, 0.121 mmol of substrate). HPLC purity 99%. ^{1}H NMR (500 MHz, DMSO-d_6): δ = 7.75 (s, 1H), 7.47 (br. d, J = 8.8 Hz, 2H), 7.10 (br. d, J = 8.8 Hz, 2H), 4.96 (dd, J = 8.4, 6.4 Hz, 1H), 4.30 (dd, J = 11.1, 8.4 Hz, 1H), 4.24 (dd, J = 11.1, 6.4 Hz, 1H), 4.04 (t, J = 5.5 Hz, 2H), 3.82 (s, 3H), 3.49 (t, J = 4.5 Hz, 4H), 2.43-2.46 (m, 2H), 2.21-2.29 (m, 4H). ^{13}C NMR (126 MHz, DMSO-d_6): δ = 165.88, 160.34, 149.71, 136.47, 130.91, 130.38, 118.44, 114.58, 65.89, 64.05, 56.09, 55.34, 53.15, 44.57, 41.29. HRMS (ESI-TOF, pos.): m/z calcd for $C_{19}H_{25}N_3O_4S$ $[M+H]^+$ 390.1482, found 390.1481.

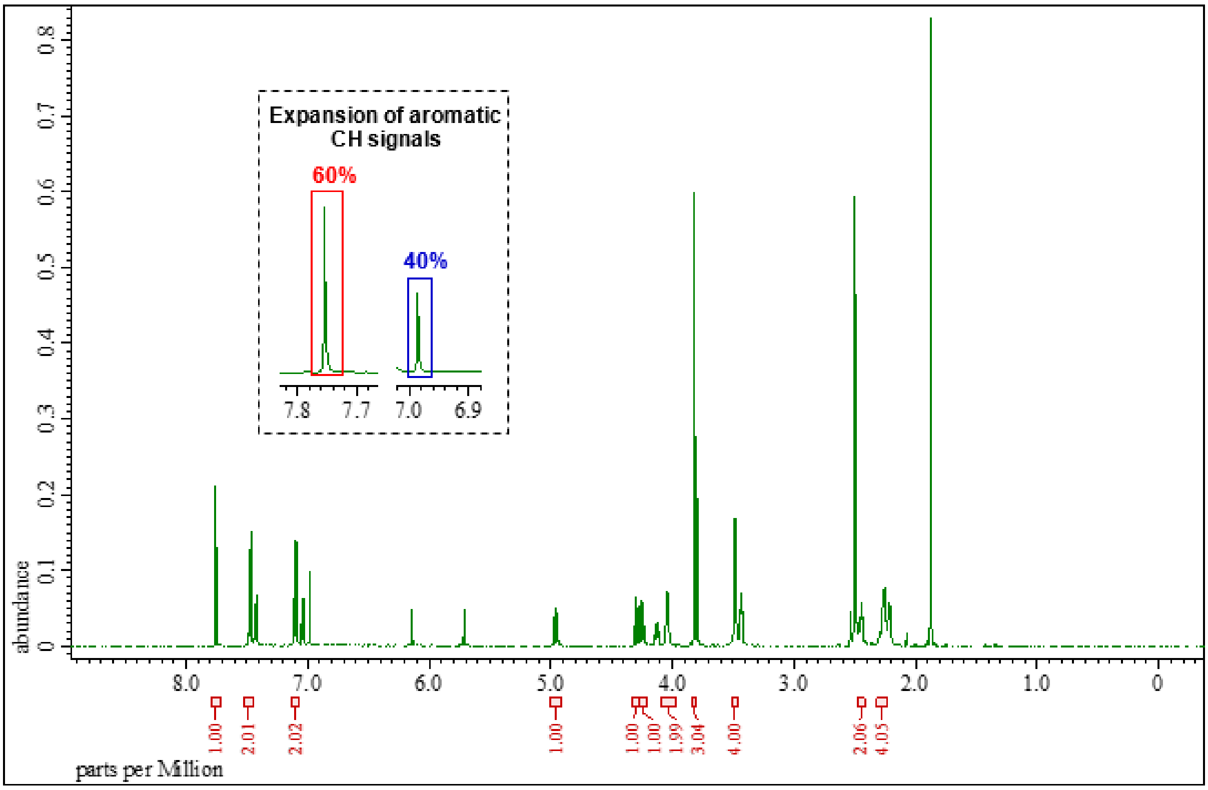
Expansion of aromatic CH signals
60%
40%
7.8
7.7
7.0
6.9
abundance
parts per Million
1.00
2.01
2.02
1.00
1.00
1.00
1.99
3.04
4.00
2.06
4.05

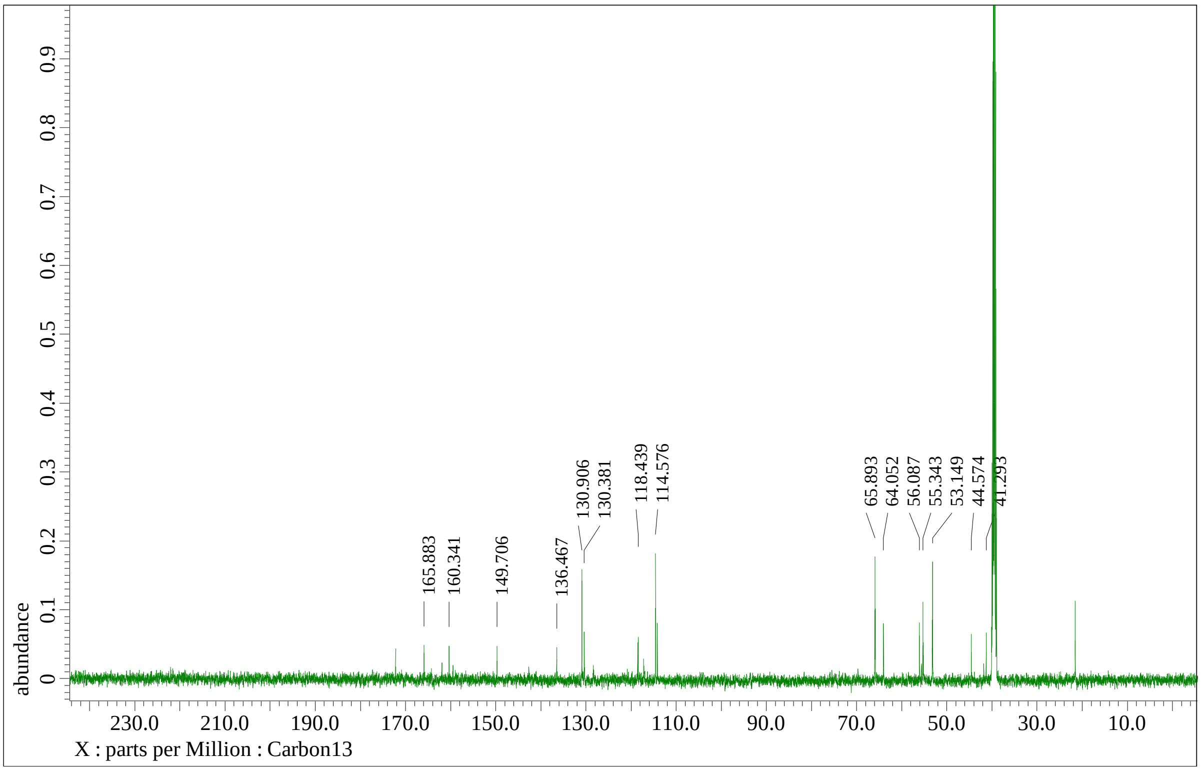
165.883
160.341
149.706
136.467
130.906
130.381
118.439
114.576
65.893
64.052
56.087
55.343
53.149
44.574
41.293
abundance
X : parts per Million : Carbon13

2-(4-(4-methoxyphenyl)-3-(2-morpholinoethyl)-2-thioxo-2,3-dihydro-1*H*-imidazol-1-yl)acrylic acid 9{*3,6*}

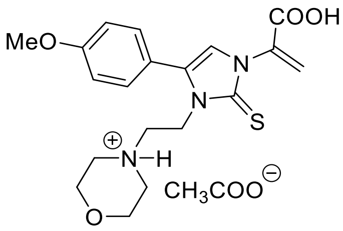

NMR purity 99%, yield quantitative (calculated from **8**{*3,6*}). ^{1}H NMR (500 MHz, DMSO-d_6): δ = 11.79 (br. s, 1H), 7.46 (br. d, J = 8.8 Hz, 2H), 7.18 (s, 1H), 7.05 (br. d, J = 8.8 Hz, 2H), 6.37 (d, J = 0.7 Hz, 1H), 5.99 (d, J = 0.7 Hz, 1H), 4.12 (t, J = 6.9 Hz, 2H), 3.81 (s, 3H), 3.80 (dd, J = 3.4, 1.3 Hz, 2H), 3.43 (t, J = 4.5 Hz, 4H), 2.47 (t, J = 6.9 Hz, 2H), 2.21 (t, J = 4.5 Hz, 2H). ^{13}C NMR (126 MHz, DMSO-d_6): δ = 163.31, 162.79, 159.70, 130.47, 130.08, 124.05, 120.36, 116.33, 114.24, 65.95, 55.35, 55.26, 55.22, 53.19, 41.97. HRMS (ESI-TOF, pos.): m/z calcd for $C_{19}H_{25}N_3O_4S$ $[M+H]^+$ 390.1482, found 390.1481.

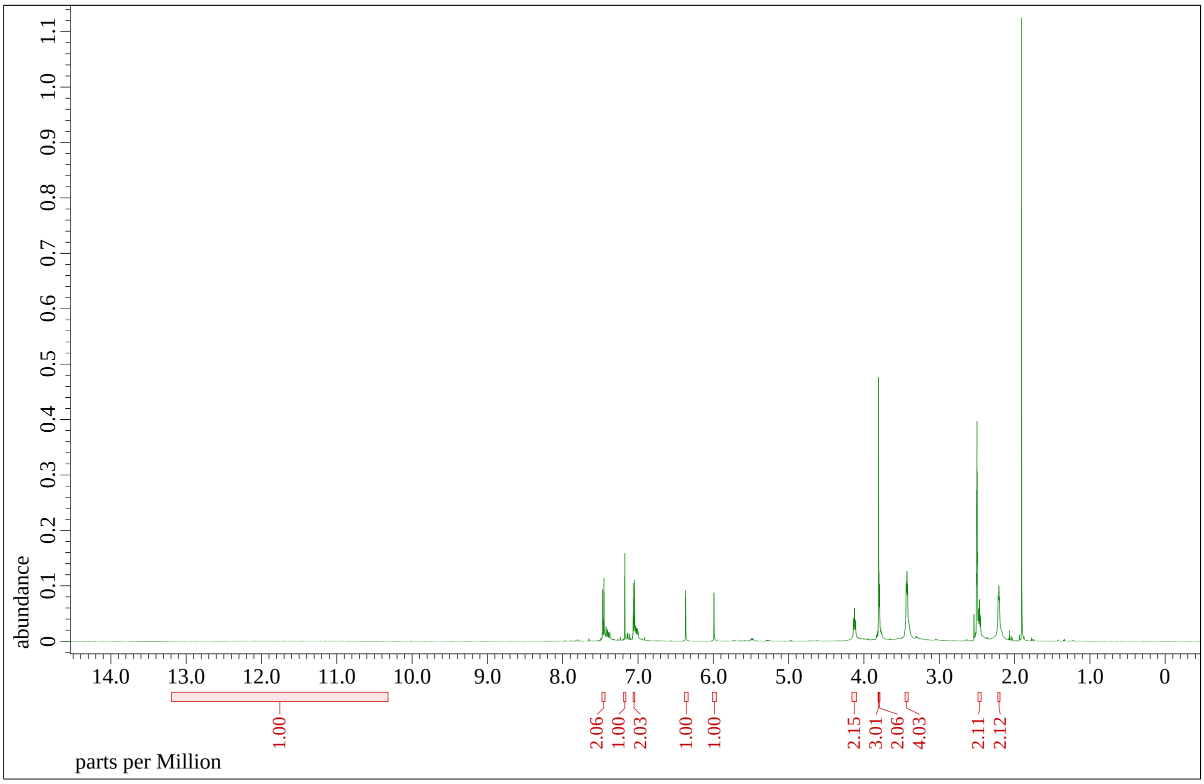

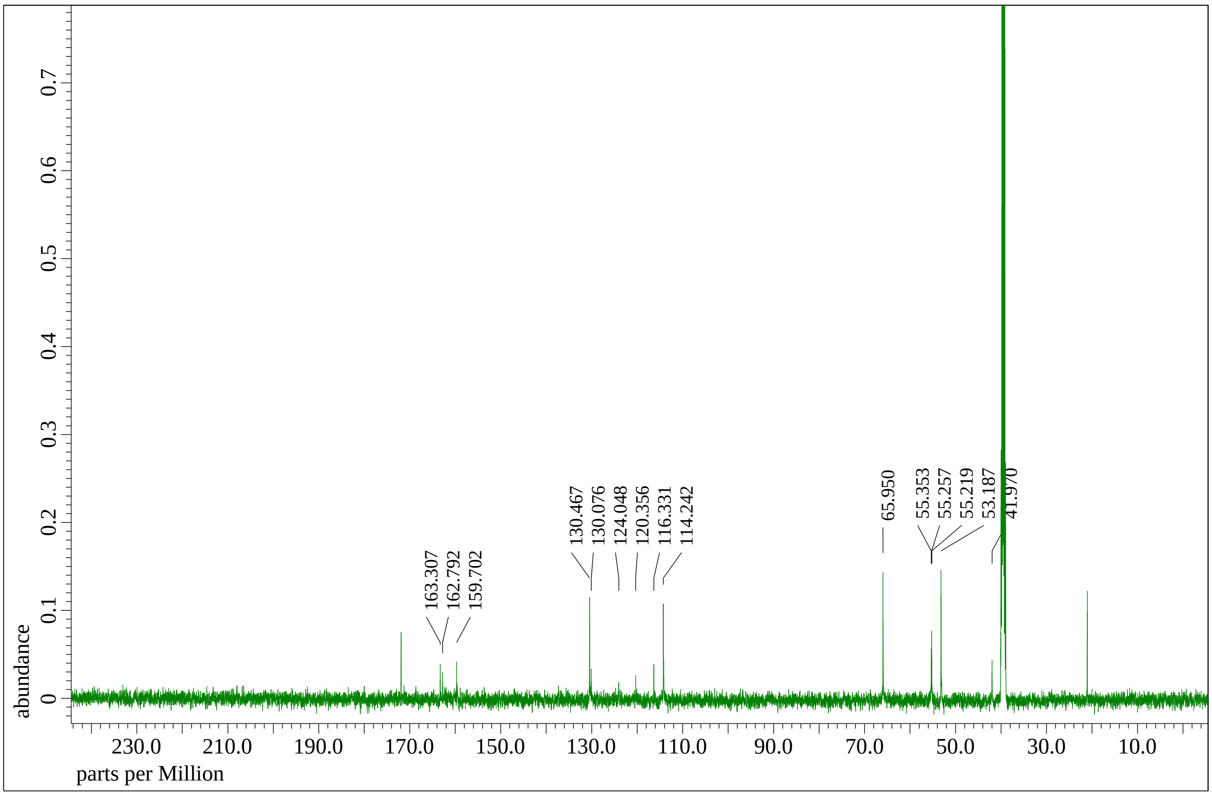

(3*R*)-7-ethyl-6-(4-fluorophenyl)-2,3-dihyhydroimidazo[2,1-*b*]thiazol-7-ium-3-carboxylate 8{*4,4*}

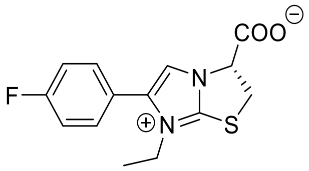

Creme amorphous solid, 16.9 mg (57%, 0.058 mmol). Cleaved from 180.0 mg of resin **2**{*4,4*} (0.565 mmol/g, 0.102 mmol of substrate). HPLC purity 99%. ^{1}H NMR (500 MHz, DMSO-d_6): δ =^{1}H NMR (500 MHz, DMSO-d_6): δ = 7.85 (s, 1H), 7.61 (br. dd, J = 8.8, 5.4 Hz, 2H), 7.41 (br. dd, J = 8.8, 8.8 Hz, 2H), 4.93 (dd, J = 8.4, 6.6 Hz, 1H), 4.35 (dd, J = 11.1, 8.4 Hz, 1H), 4.31 (dd, J = 11.1, 6.6 Hz, 1H), 4.00 (q, J = 7.3 Hz, 2H), 1.19 (t, J = 7.3 Hz, 3H). ^{13}C NMR (126 MHz, DMSO-d_6): δ = 165.35, 162.85 (d, J = 247.1 Hz), 148.88, 135.06, 131.66 (d, J = 8.4 Hz), 122.80, 119.26, 116.26 (d, J = 21.6 Hz), 64.01, 42.38, 41.56, 13.81. HRMS (ESI-TOF, pos.): *m/z* calcd for $C_{14}H_{14}FN_2O_2S$ [M+H]$^+$ 293.0755, found 293.0754.

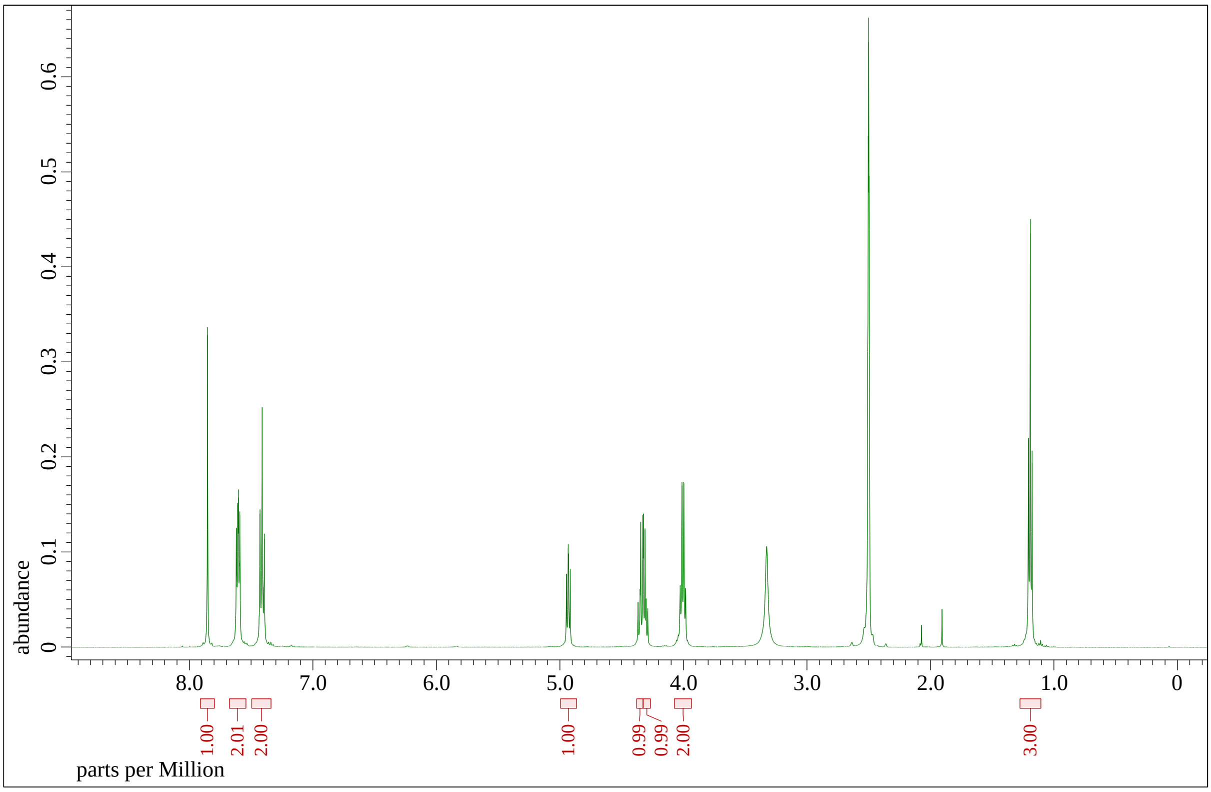
abundance
0
0.1
0.2
0.3
0.4
0.5
0.6
8.0
7.0
6.0
5.0
4.0
3.0
2.0
1.0
0
1.00
2.01
2.00
1.00
0.99
0.99
2.00
3.00
parts per Million

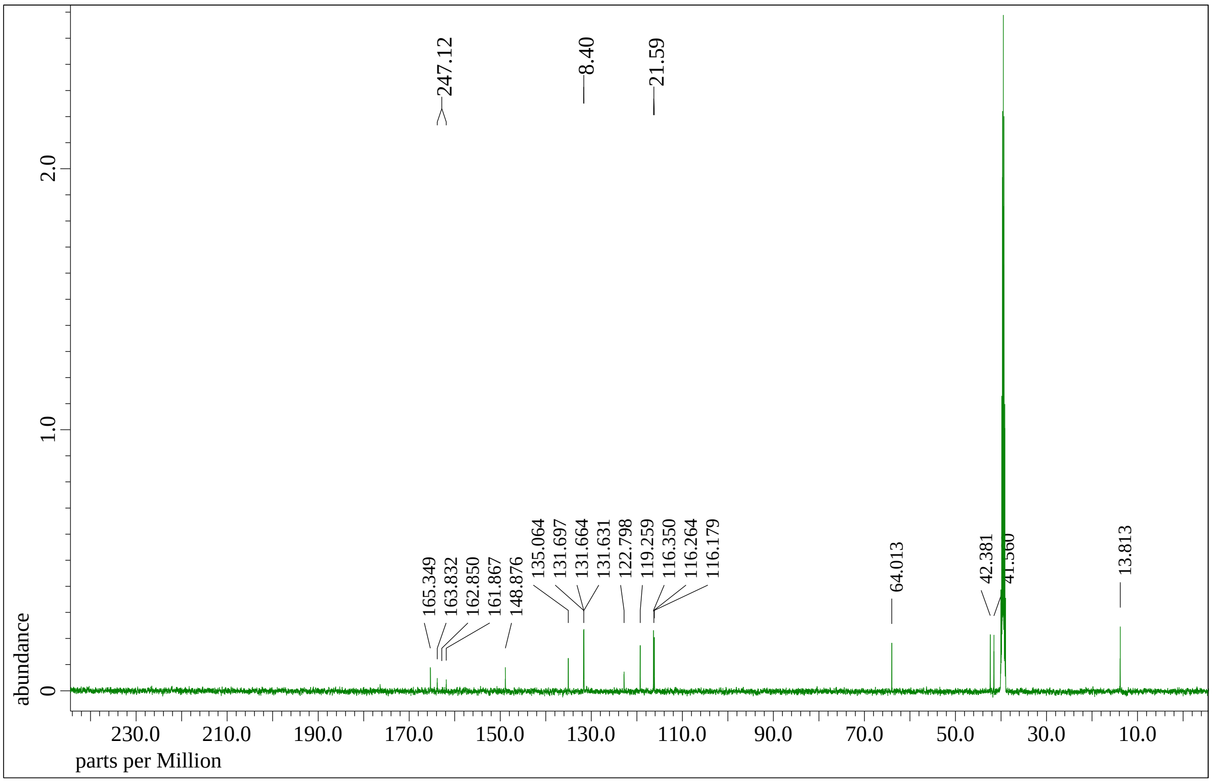
247.12
8.40
21.59
abundance
0
1.0
2.0
165.349
163.832
162.850
161.867
148.876
135.064
131.697
131.664
131.631
122.798
119.259
116.350
116.264
116.179
64.013
42.381
41.560
13.813
230.0
210.0
190.0
170.0
150.0
130.0
110.0
90.0
70.0
50.0
30.0
10.0
parts per Million

2-(3-ethyl-4-(4-fluorophenyl)-2-thioxo-2,3-dihydro-1*H*-imidazol-1-yl)acrylic acid 9{*4,4*}

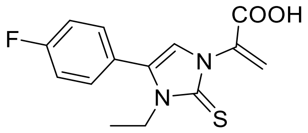

NMR: Mixture with 16% of **8**{*4,4*}. ^{1}H NMR (500 MHz, DMSO-d_6): δ = 13.23 (br. s, 1H), 7.57 (br. dd, J = 8.8, 5.4 Hz, 2H), 7.36 (br. dd, J = 8.8, 8.8 Hz, 2H), 7.31 (s, 1H), 6.41 (d, J = 0.7 Hz, 1H), 6.05 (d, J = 0.7 Hz, 1H), 4.04 (q, J = 7.1 Hz, 2H), 1.10 (t, J = 7.1 Hz, 3H). ^{13}C NMR (126 MHz, DMSO-d_6): δ = 163.13, 162.73, 162.38 (d, J = 247.1 Hz), 136.60, 131.17 (d, J = 8.4 Hz), 128.87, 124.89, 124.53 (d, J = 2.4 Hz), 117.02, 115.97 (d, J = 21.6 Hz), 38.99, 13.36. HRMS (ESI-TOF, pos.): *m/z* calcd for $C_{14}H_{14}FN_2O_2S$ $[M+H]^+$ 293.0755, found 293.0754.

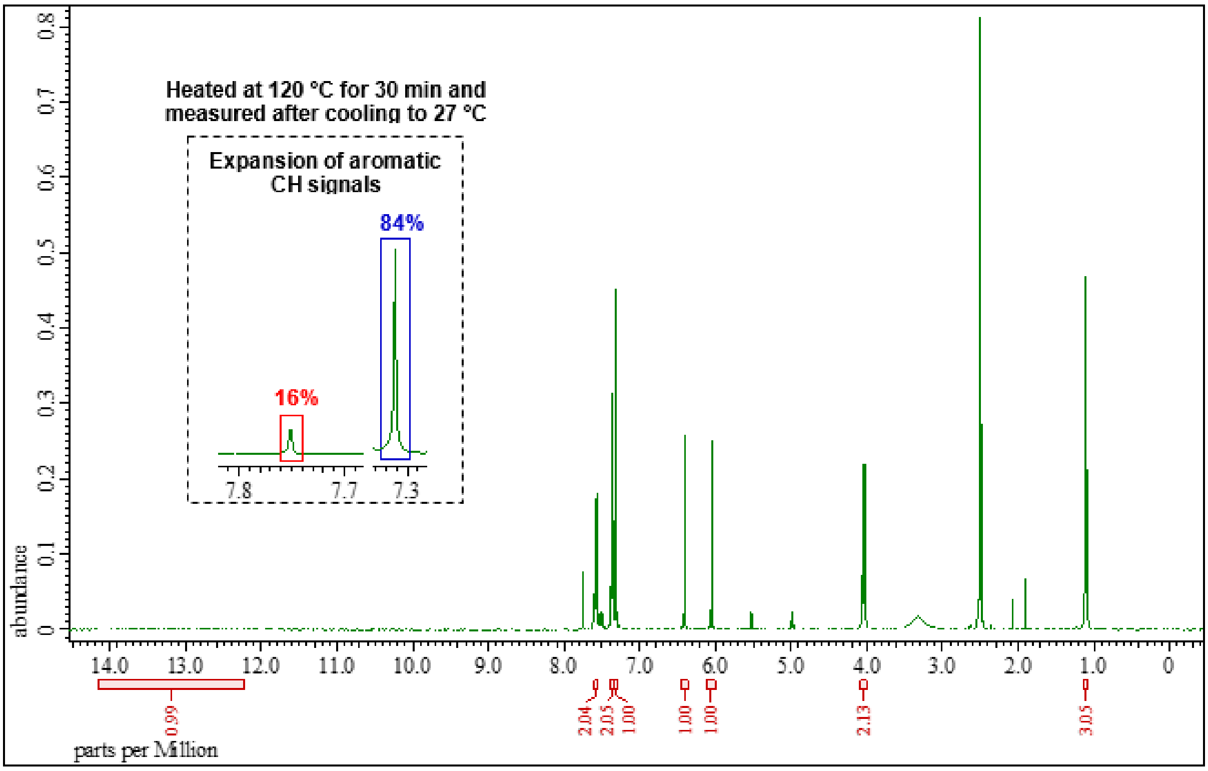

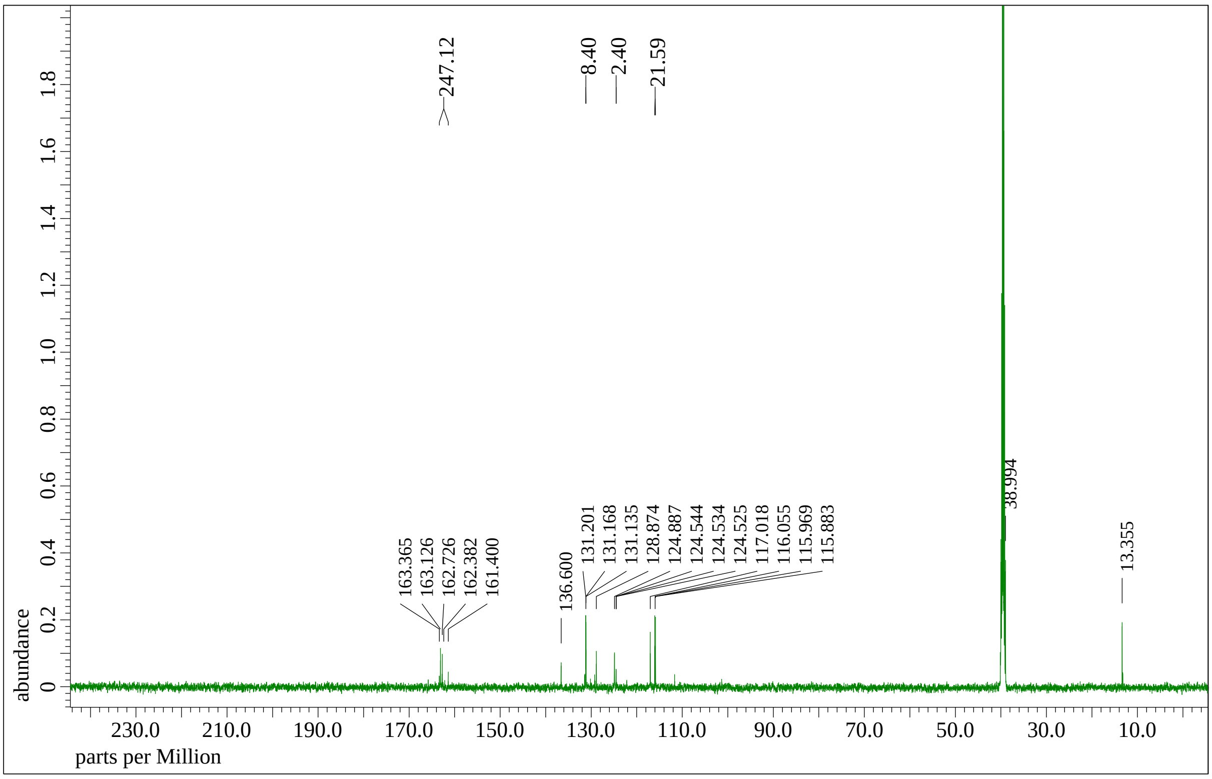
247.12
8.40
2.40
21.59
163.365
163.126
162.726
162.382
161.400
136.600
131.201
131.168
131.135
128.874
124.887
124.544
124.534
124.525
117.018
116.055
115.969
115.883
38.994
13.355
abundance
parts per Million

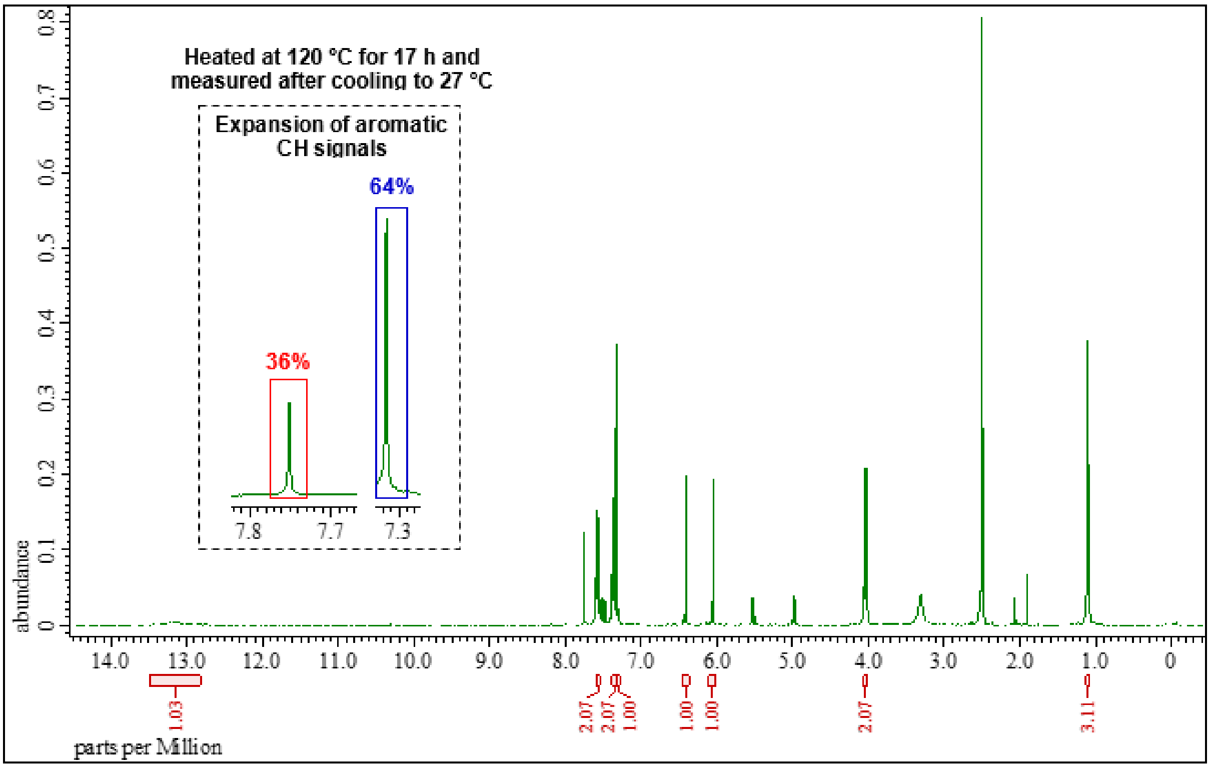
Heated at 120 °C for 17 h and measured after cooling to 27 °C
Expansion of aromatic CH signals
64%
36%
abundance
parts per Million
1.03
2.07
2.07
1.00
1.00
1.00
2.07
3.11

2-(3-(cyclohexylmethyl)-4-phenyl-2-thioxo-2,3-dihydro-1*H*-imidazol-1-yl)acrylic acid 9{*4,5*}

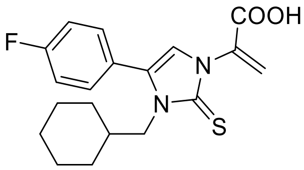

NMR: Mixture with 8% of **8**{*4,5*}. Yellow amorphous solid, 19.0 mg (19%, 0.053 mmol). Cleaved from 494.7 mg of resin **2**{*4,5*} (0.565 mmol/g, 0.280 mmol of substrate). HPLC purity 99%. ^{1}H NMR (500 MHz, DMSO-d_6): δ = 12.60 (br. s, 1H), 7.56 (br. dd, *J* = 8.8, 5.4 Hz, 2H), 7.34 (br. dd, *J* = 8.8, 8.8 Hz, 2H), 7.29 (s, 1H), 6.40 (d, *J* = 0.7 Hz, 1H), 6.03 (d, *J* = 0.7 Hz, 1H), 3.99 (d, *J* = 7.3 Hz, 2H), 1.66-1.55 (m, 1H), 1.53-1.47 (m, 2H), 1.38-1.32 (m, 2H), 1.06-0.88 (m, 4H), 0.75-0.64 (m, 2H). ^{13}C NMR (126 MHz, DMSO-d_6): δ = 163.63, 162.25 (d, *J* = 247.1 Hz), 163.13, 136.73, 131.20 (d, *J* = 8.4 Hz), 129.36, 124.94 (d, *J* = 2.4 Hz), 124.80, 116.97, 115.88 (d, *J* = 21.6 Hz), 50.08, 35.93, 29.74, 25.63, 25.01. HRMS (ESI-TOF, pos.): *m/z* calcd for $C_{19}H_{22}FN_2O_2S$ [M+H]$^+$ 361.1381, found 361.1382.

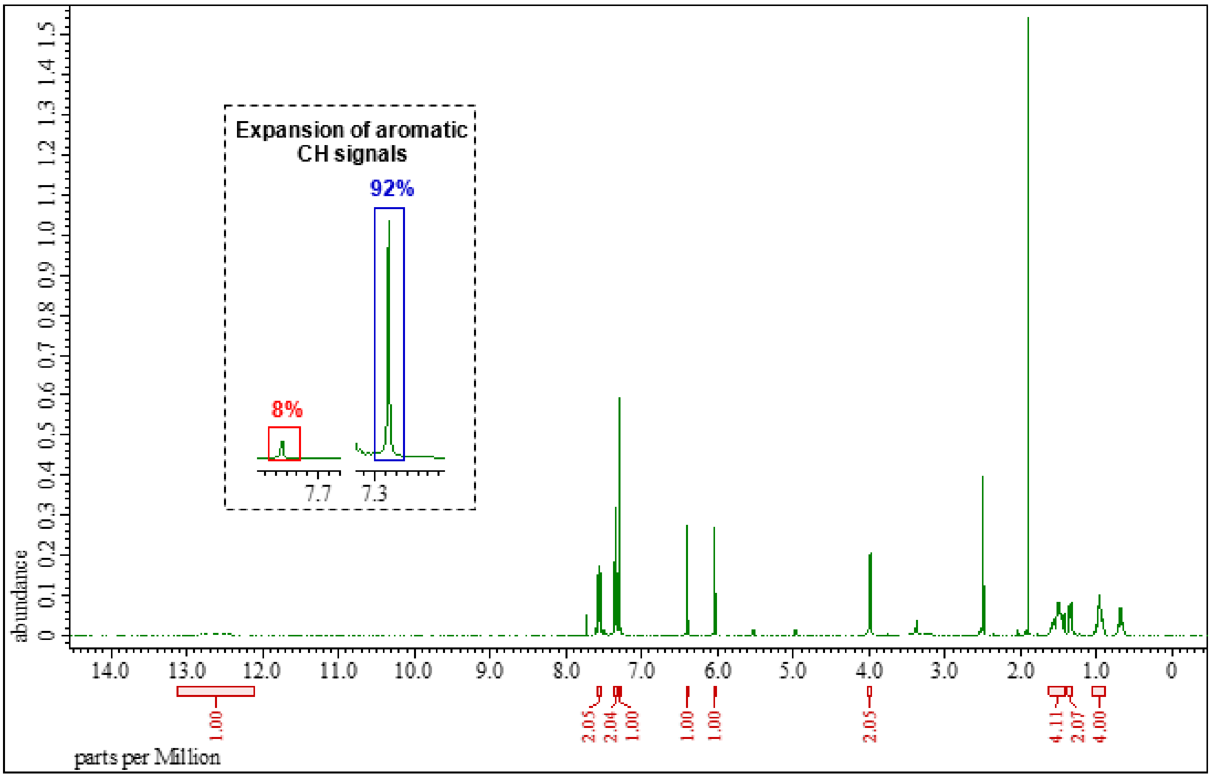

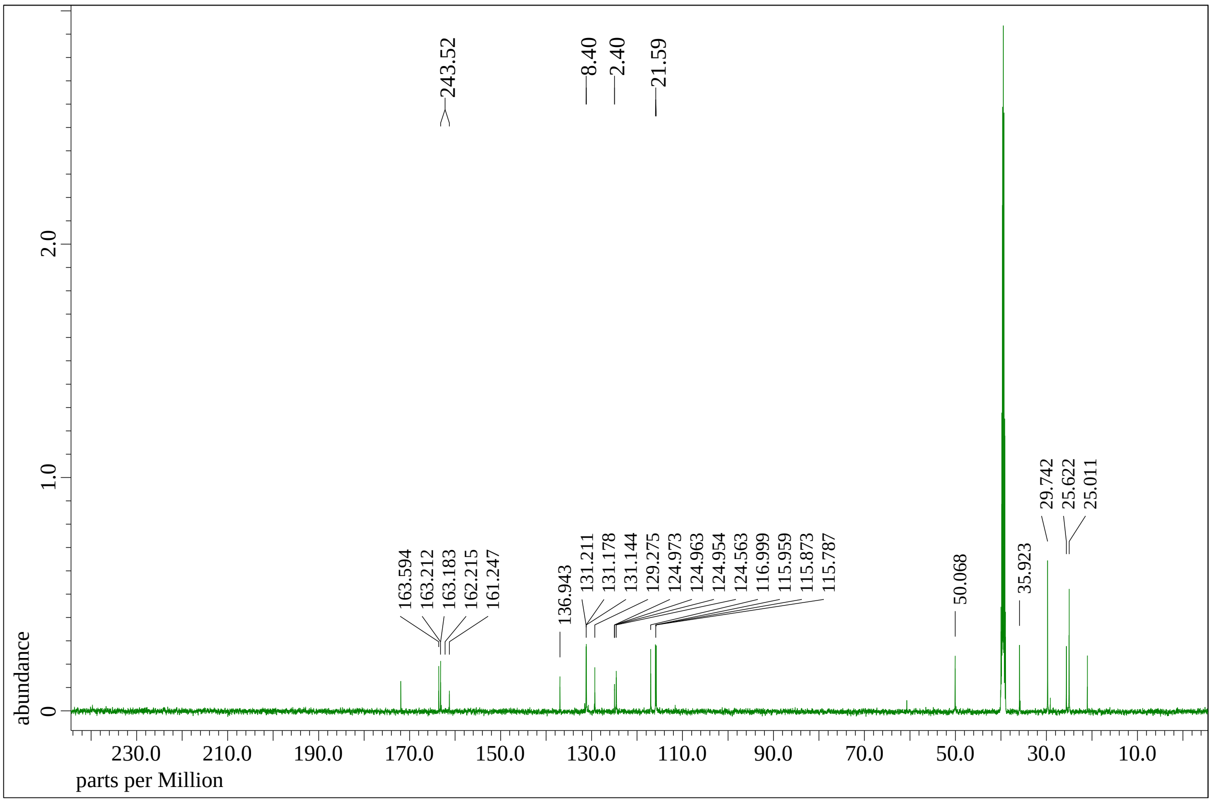

(3*R*)-7-benzyl-6-(4-bromophenyl)-2,3-dihyhydroimidazo[2,1-*b*]thiazol-7-ium-3-carboxylate 8{*5,2*}

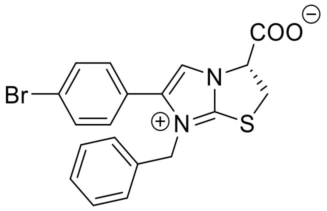

NMR: Mixture with 2% of **9**{*5,2*}. Creme amorphous solid, 26.0 mg (28%, 0.063 mmol). Cleaved from 405.1 mg of resin **2**{*5,2*} (0.556 mmol/g, 0.225 mmol of substrate). HPLC purity 98%. ^{1}H NMR (500 MHz, DMSO-d_6): δ = 7.99 (s, 1H), 7.70 (br. d, J = 8.6 Hz, 2H), 7.44 (br. d, J = 8.6 Hz, 2H), 7.34-7.35 (m, 3H), 7.12-7.14 (m, 2H), 5.29 (s, 2H), 5.02 (dd, J = 8.6, 6.0 Hz, 1H), 4.30 (dd, J = 11.1, 8.6 Hz, 1H), 4.23 (dd, J = 11.1, 6.0 Hz, 1H). ^{13}C NMR (126 MHz, DMSO-d_6): δ = 165.77, 150.50, 135.45, 133.01, 132.08, 131.15, 128.89, 128.66, 127.81, 125.41, 123.48, 119.64, 63.78, 50.28, 41.52. HRMS (ESI-TOF, pos.): *m/z* calcd for $C_{19}H_{16}BrN_2O_2S$ [M+H]$^+$ 415.0110, found 415.0117.

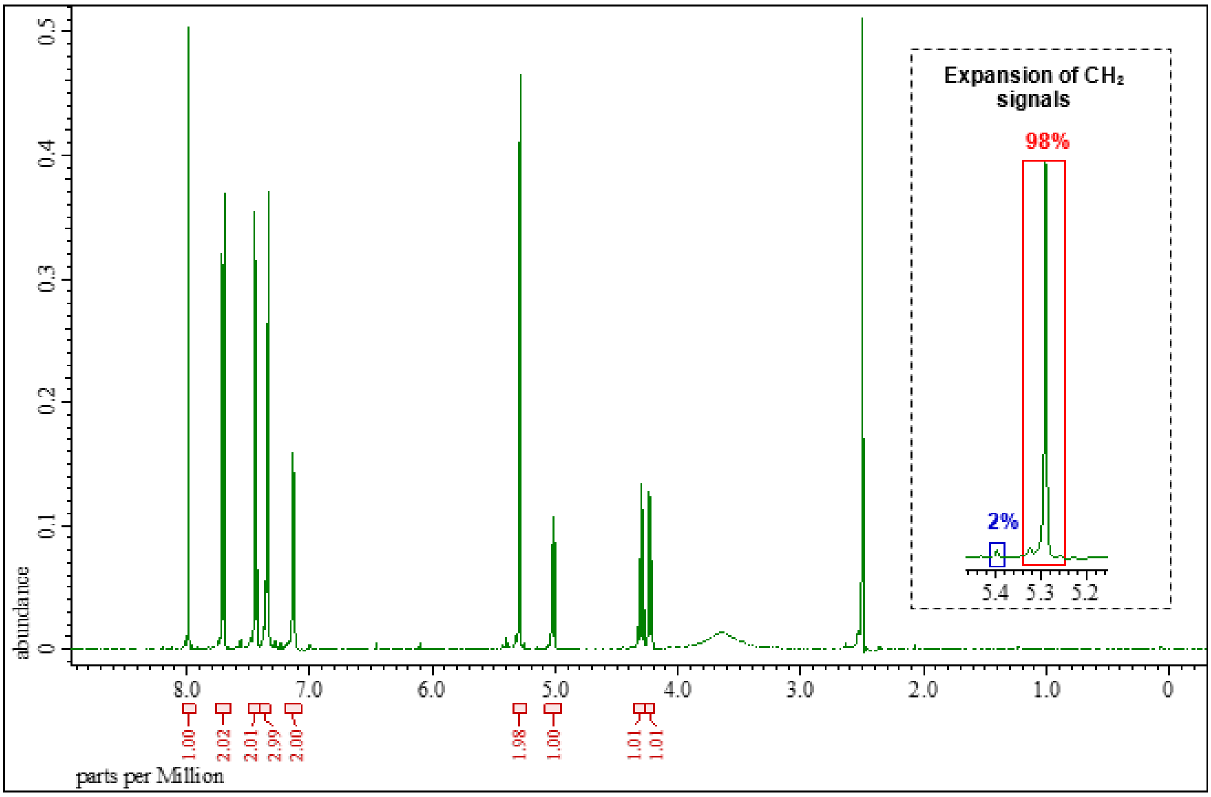
Expansion of CH₂ signals
98%
2%
5.4
5.3
5.2
abundance
0
0.1
0.2
0.3
0.4
0.5
8.0
7.0
6.0
5.0
4.0
3.0
2.0
1.0
0
1.00
2.02
2.01
2.99
2.00
1.98
1.00
1.01
1.01
parts per Million

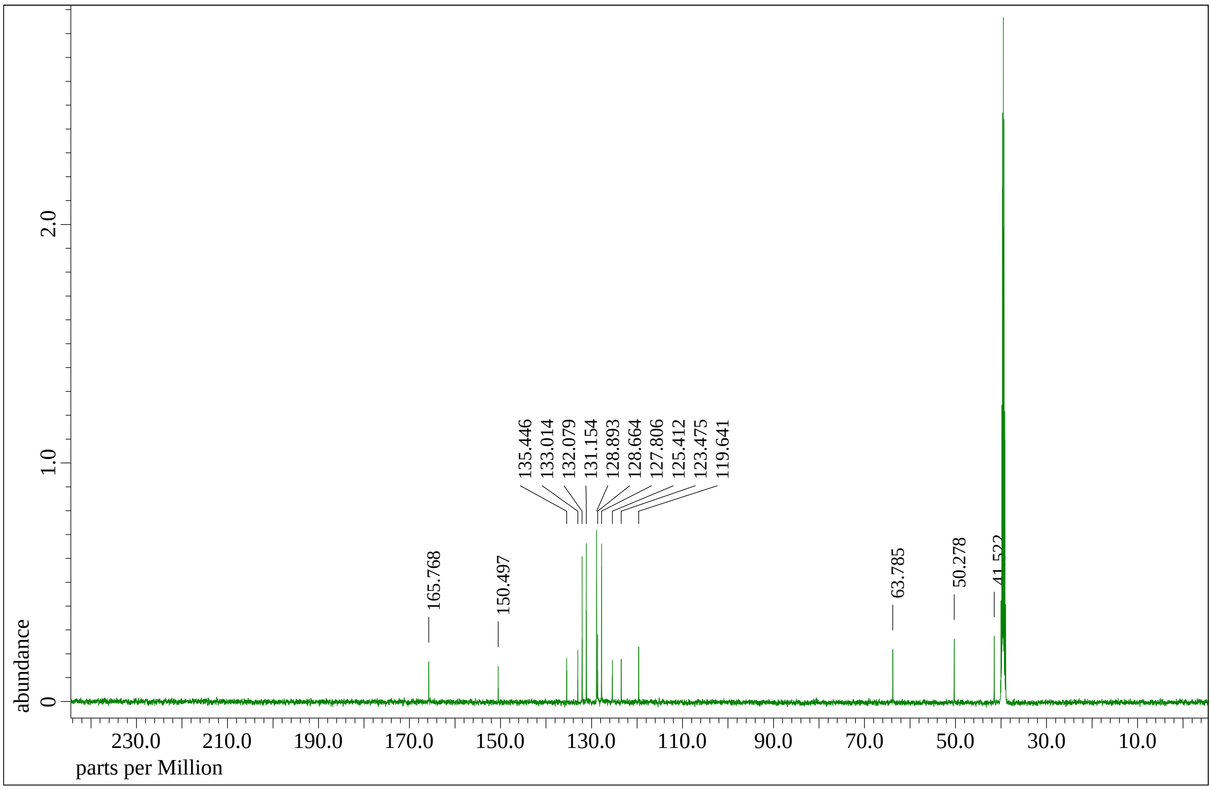
135.446
133.014
132.079
131.154
128.893
128.664
127.806
125.412
123.475
119.641
165.768
150.497
63.785
50.278
41.522
abundance
0
1.0
2.0
230.0
210.0
190.0
170.0
150.0
130.0
110.0
90.0
70.0
50.0
30.0
10.0
parts per Million

2-(3-benzyl-4-(4-bromophenyl)-2-thioxo-2,3-dihydro-1*H*-imidazol-1-yl)acrylic acid 9{*5,2*}

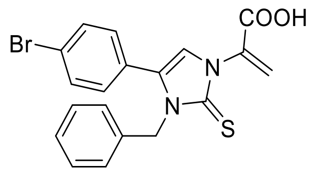

NMR: Mixture with 9% of **8**{*5,2*}. ^{1}H NMR (500 MHz, DMSO-d_6): δ = 13.37 (br. s, 1H), 7.57 (br. d, J = 8.6 Hz, 2H), 7.47 (s, 1H), 7.18-7.30 (m, 5H), 7.00 (br. d, J = 8.6 Hz, 2H), 6.47 (d, J = 0.7 Hz, 1H), 6.14 (d, J = 0.7 Hz, 1H), 5.39 (s, 2H). ^{13}C NMR (126 MHz, DMSO-d_6): δ = 164.69, 163.03, 136.58, 136.32, 131.71, 130.61, 130.36, 129.21, 128.38, 127.20, 126.43, 125.42, 122.25, 117.65, 47.97. HRMS (ESI-TOF, pos.): m/z calcd for $C_{19}H_{16}BrN_2O_2S$ $[M+H]^+$ 415.0110, found 415.0117.

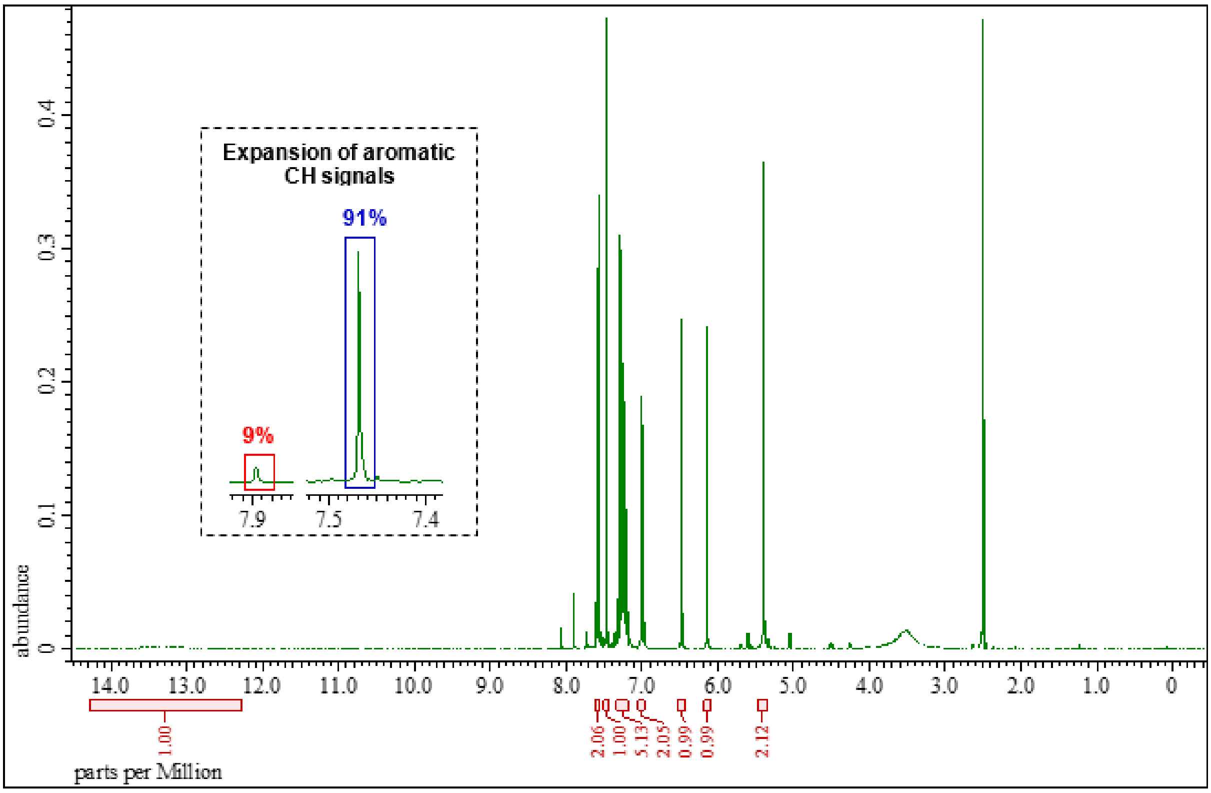

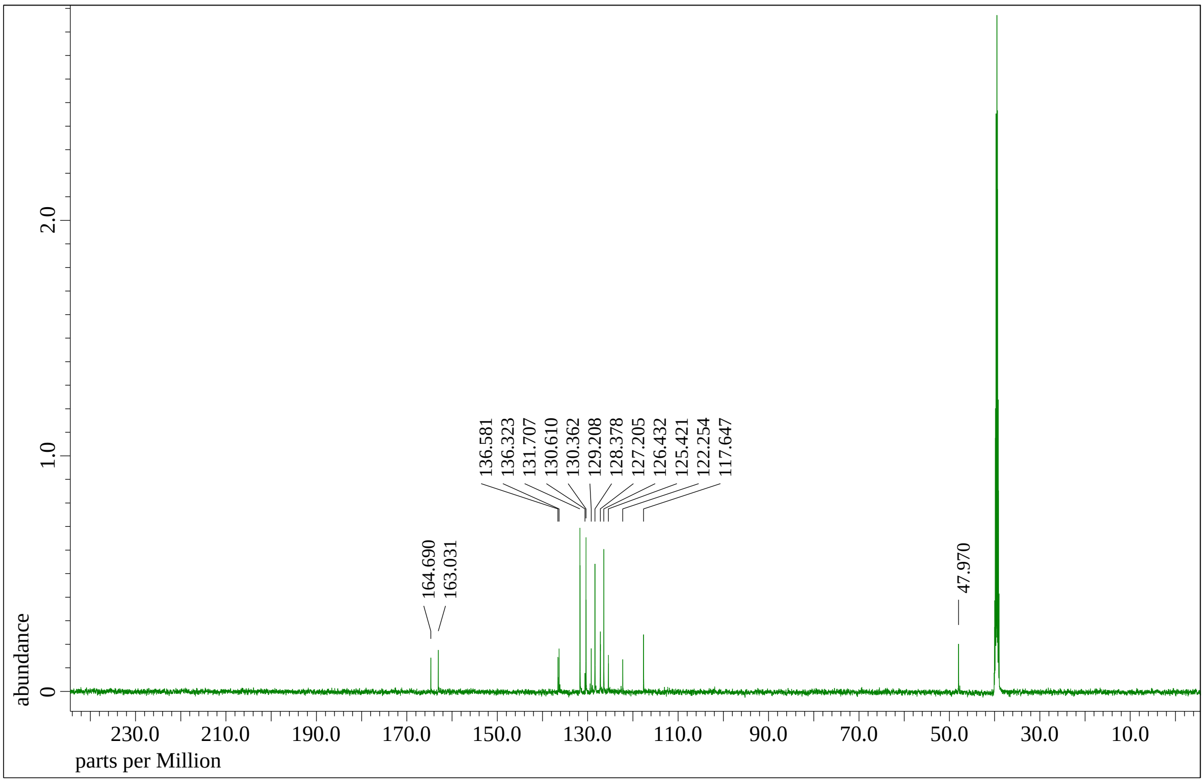

(3*R*)-7-allyl-6-(4-bromophenyl)-2,3-dihyhydroimidazo[2,1-*b*]thiazol-7-ium-3-carboxylate 8{*5,3*}

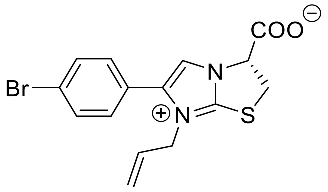

Creme amorphous solid, 17.5 mg (18%, 0.048 mmol). Cleaved from 483.9 mg of resin **2**{*5,3*} (0.556 mmol/g, 0.269 mmol of substrate). HPLC purity 99%. ^{1}H NMR (500 MHz, DMSO-d_6): δ = 7.96 (s, 1H), 7.75 (br. d, J = 8.6 Hz, 2H), 7.46 (br. d, J = 8.6 Hz, 2H), 5.86-5.94 (m, 1H), 5.32 (br. d, J = 10.4 Hz, 1H), 5.14 (br. d, J = 17.2 Hz, 1H), 4.95 (dd, J = 8.4, 6.2 Hz, 1H), 4.65 (ddd, J = 4.7, 1.4, 1.4 Hz, 2H), 4.33 (dd, J = 11.0, 8.4 Hz, 1H), 4.28 (dd, J = 11.0, 6.2 Hz, 1H). ^{13}C NMR (126 MHz, DMSO-d_6): δ = 165.35, 150.22, 135.32, 132.14, 130.90, 130.21, 125.42, 123.40, 119.65, 119.36, 64.08, 49.09, 41.68. HRMS (ESI-TOF, pos.): *m/z* calcd for $C_{15}H_{14}BrN_2O_2S$ [M+H]$^+$ 364.9954, found 364.9933.

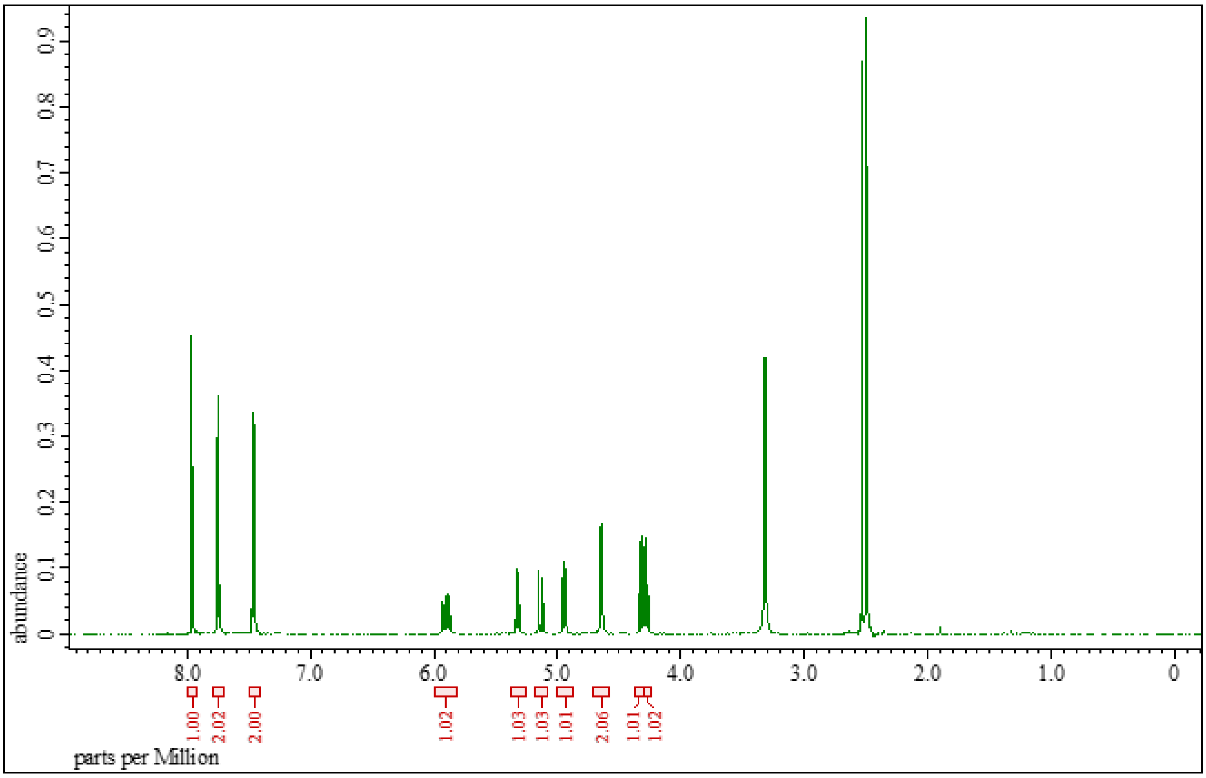
abundance
parts per Million
1.00
2.02
2.00
1.02
1.03
1.03
1.01
2.06
1.01
1.02

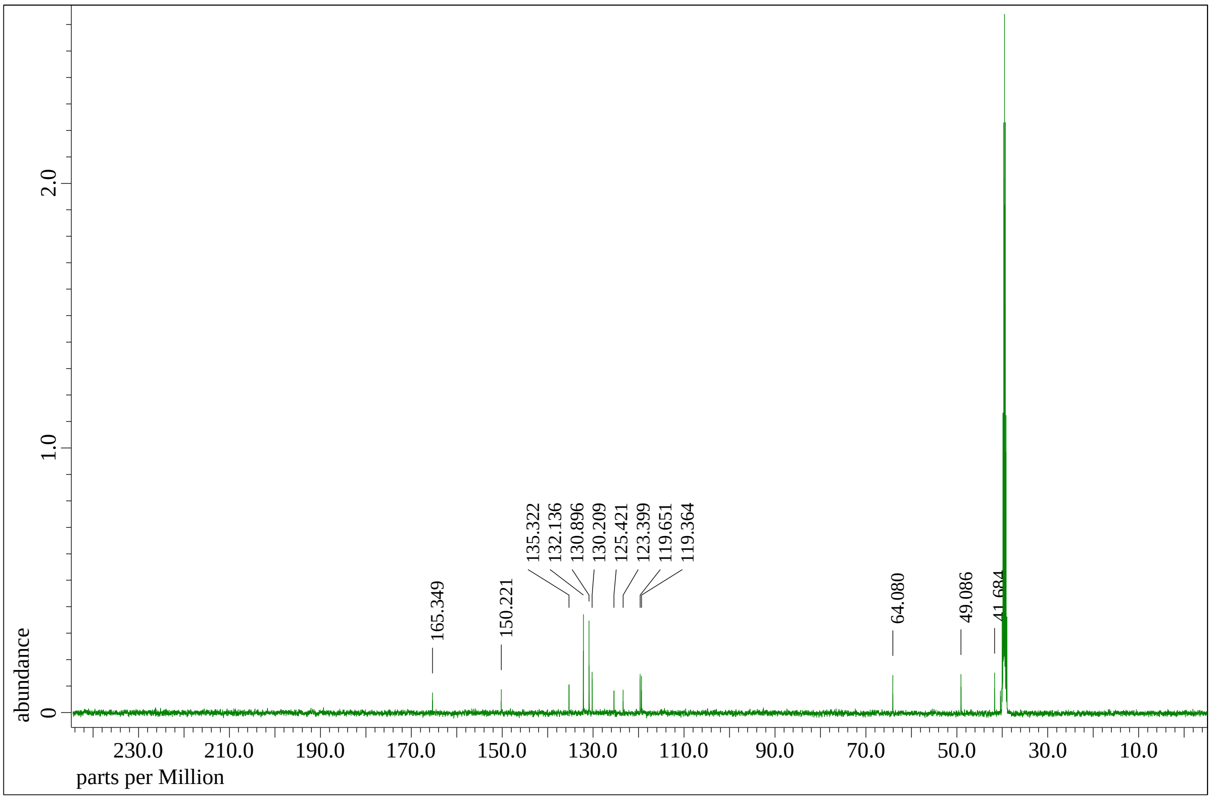
abundance
parts per Million
165.349
150.221
135.322
132.136
130.896
130.209
125.421
123.399
119.651
119.364
64.080
49.086
41.684

2-(3-allyl-4-(4-bromophenyl)-2-thioxo-2,3-dihydro-1*H*-imidazol-1-yl)acrylic acid 9{*5,3*}

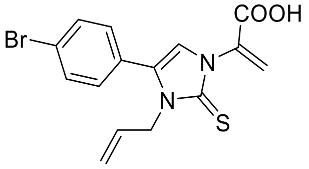

NMR: Mixture with 29% of **8**{*5,3*}. ^{1}H NMR (500 MHz, DMSO-d_6): δ = 13.27 (br. s, 1H), 7.68 (br. d, J = 8.6 Hz, 2H), 7.45 (br. d, J = 8.6 Hz, 2H), 7.44 (s, 1H), 6.44 (d, J = 0.7 Hz, 1H), 6.08 (d, J = 0.7 Hz, 1H), 5.77-5.84 (m, 1H), 5.11 (br. d, J = 10.5 Hz, 1H), 4.84 (br. d, J = 17.3 Hz, 1H), 4.72 (ddd, J = 4.7, 1.4, 1.4 Hz, 2H). ^{13}C NMR (126 MHz, DMSO-d_6): δ = 163.99, 163.04, 132.22, 131.81, 130.59, 130.36, 129.17, 127.25, 125.23, 122.24, 117.32, 116.96, 47.09. HRMS (ESI-TOF, pos.): *m/z* calcd for $C_{15}H_{14}BrN_2O_2S$ $[M+H]^+$ 364.9954, found 364.9933.

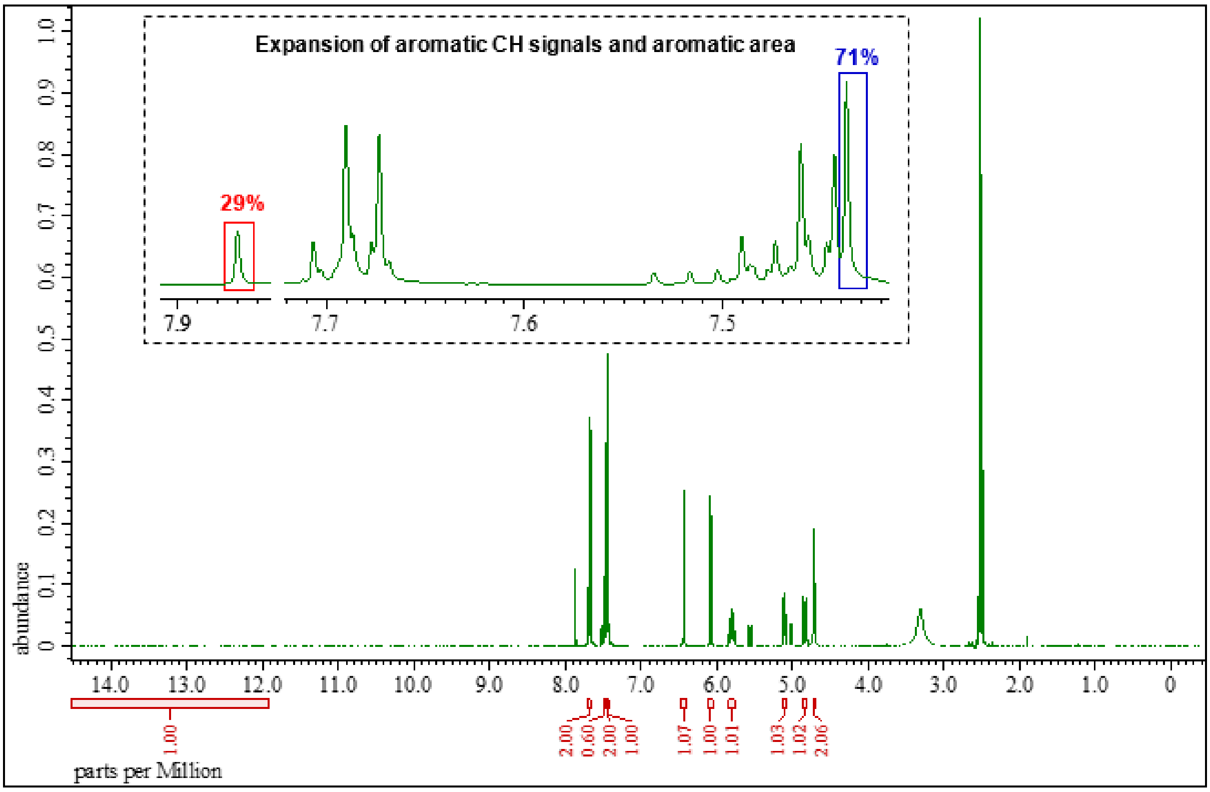

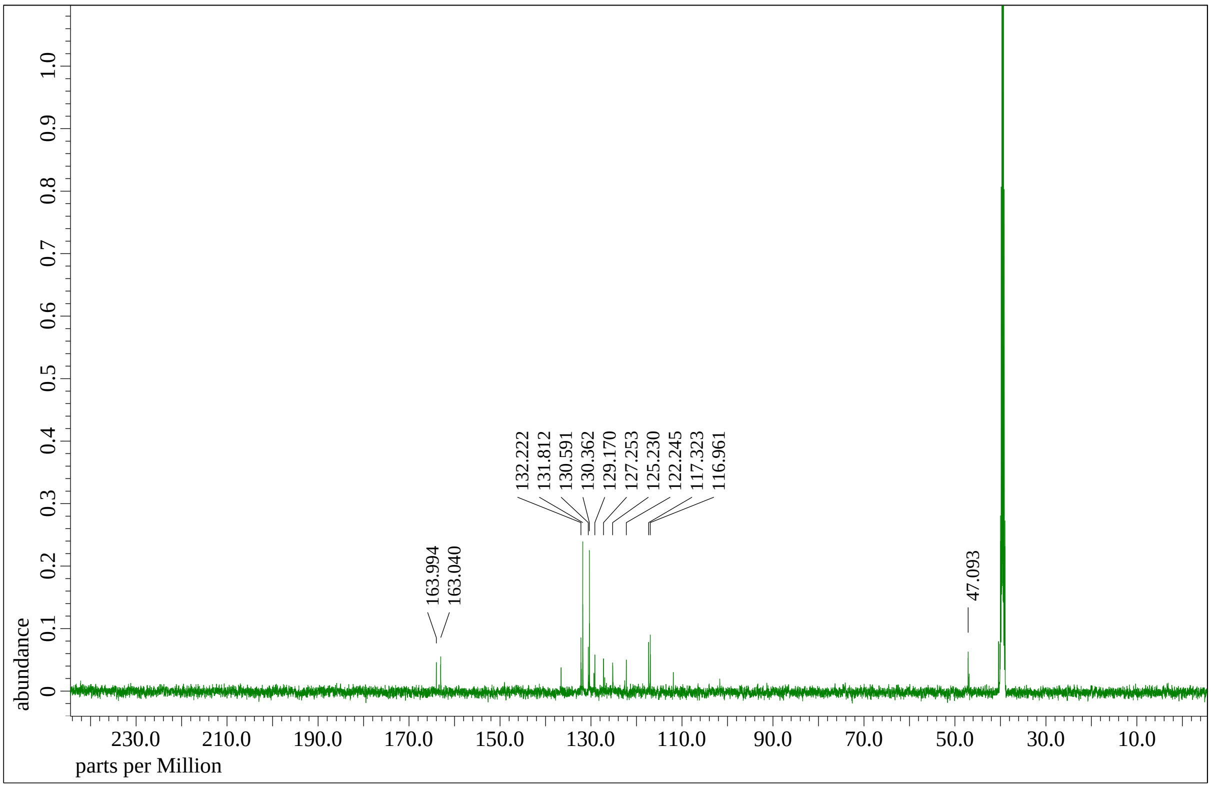

(3*R*)-6-(4-bromophenyl)-7-(2-morpholinoethyl)-2,3-dihyhydroimidazo[2,1-*b*]thiazol-7-ium-3-carboxylate 8{*5,6*}

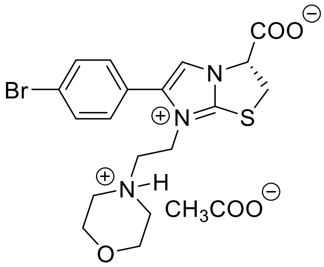

NMR: Mixture of 12% with **9**{*5,6*}. Creme amorphous solid, 10.5 mg (17.3%, 0.024 mmol) of which **8** 10.3 mg (17%, 0.024 mmol) and **9** 0.2 mg (0.3%, 0.0006 mmol). Cleaved from 244.2 mg of resin **2**{*5,6*} (0.556 mmol/g, 0.136 mmol of substrate). HPLC purity 99%. ^{1}H NMR (500 MHz, DMSO-d_6): δ = 7.89 (1H), 7.76 (d, J = 8.6 Hz, 2H), 7.52 (d, J = 8.6 Hz, 2H), 4.95 (dd, J = 8.2, 6.6 Hz, 1H), 4.29 (dd, J = 11.1, 8.2 Hz, 1H), 4.25 (dd, J = 11.1, 6.6 Hz, 1H), 4.08 (t, J = 5.5 Hz, 2H), 3.46 (t, J = 4.5 Hz, 4H), 2.44-2.48 (m, 2H), 2.21-2.29 (m, 4H). ^{13}C NMR (126 MHz, DMSO-d_6): δ = 165.42, 150.55, 135.41, 132.15, 131.21, 125.81, 123.37, 119.25, 65.93, 65.85, 56.16, 53.26, 53.16, 41.41. HRMS (ESI-TOF, pos.): *m/z* calcd for $C_{18}H_{21}BrN_3O_3S$ $[M+H]^+$ 438.0482, found 438.0482.

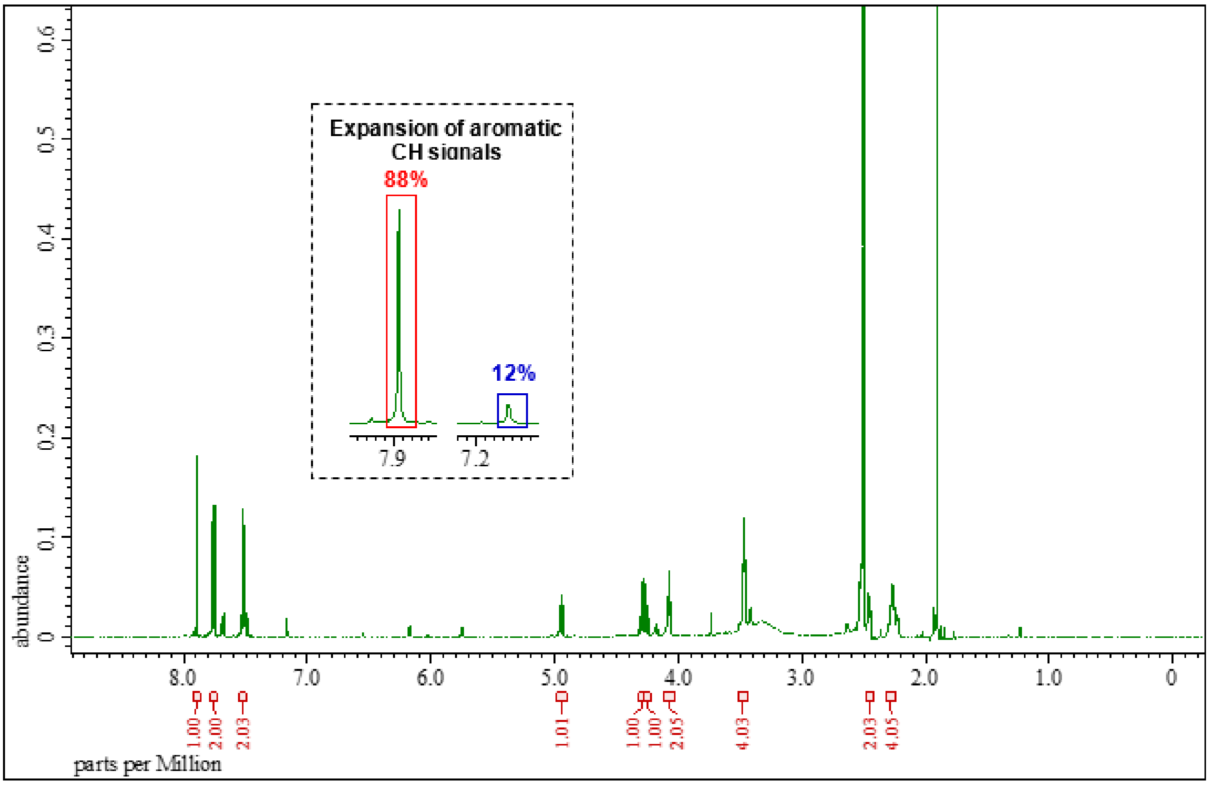

Expansion of aromatic CH signals
88%
12%
7.9
7.2
abundance
parts per Million
1.00
2.00
2.03
1.01
1.00
1.00
2.05
4.03
2.03
4.05

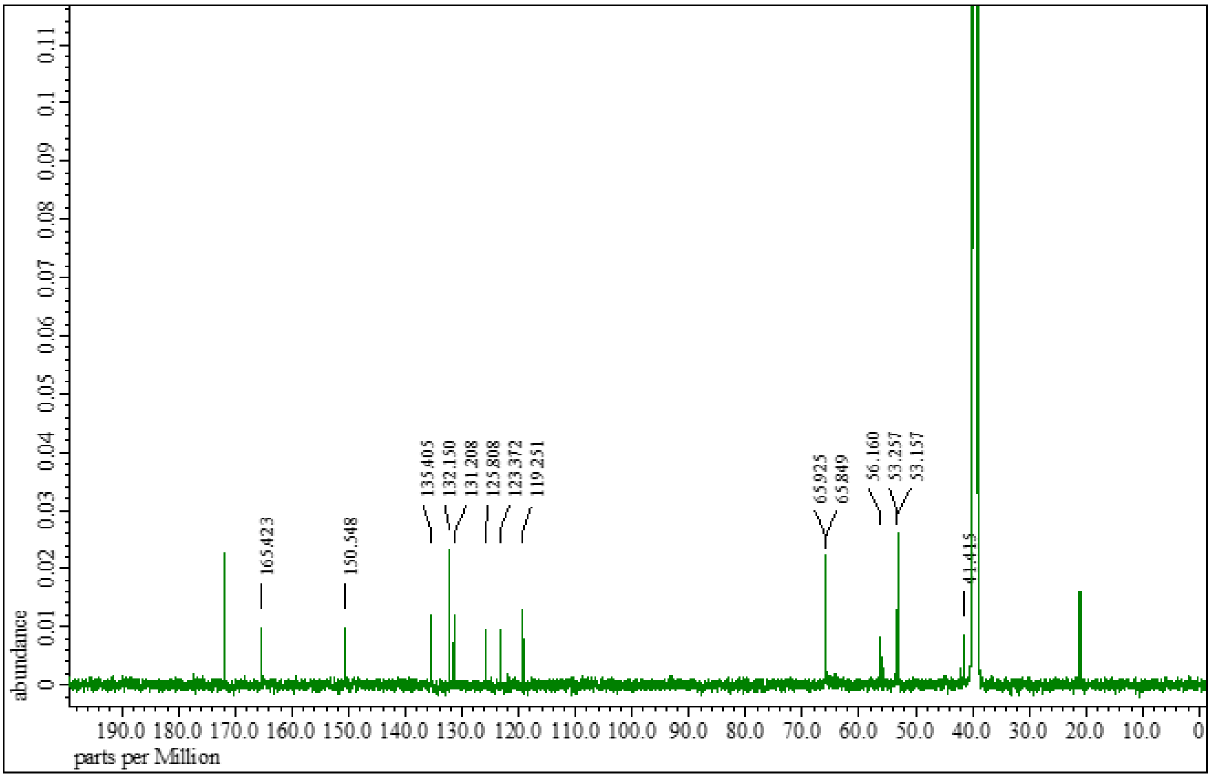

165.423
150.548
135.405
132.150
131.208
125.808
123.372
119.251
65.925
65.849
56.160
53.257
53.157
41.415
abundance
parts per Million

2-(4-(4-bromophenyl)-3-(2-morpholinoethyl)-2-thioxo-2,3-dihydro-1*H*-imidazol-1-yl)acrylic acid 9{*5,6*}

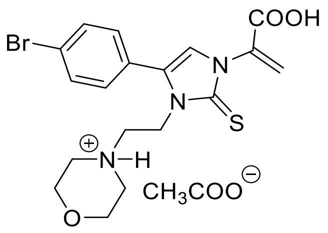

NMR purity 99%, yield quantitative (calculated from **8**{*5,6*}).^{1}H NMR (500 MHz, DMSO-d_6): δ = 12.07 (br. s, 1H), 7.71 (br. d, J = 8.6 Hz, 2H), 7.52 (br. d, J = 8.6 Hz, 2H), 7.35 (s, 1H), 6.40 (d, J = 0.7 Hz, 1H), 6.03 (d, J = 0.7 Hz, 1H), 4.17 (t, J = 6.8 Hz, 2H), 3.40 (t, J = 4.5 Hz, 4H), 3.37 (t, J = 4.5 Hz, 2H), 2.47 (t, J = 6.8 Hz, 2H), 2.21 (t, J = 4.5 Hz, 2H). ^{13}C NMR (126 MHz, DMSO-d_6): δ = 163.51, 163.09, 131.82, 131.03, 130.76, 129.31, 127.59, 122.21, 117.17, 111.69, 65.92, 55.39, 53.21, 42.24. HRMS (ESI-TOF, pos.): m/z calcd for $C_{18}H_{21}BrN_3O_3S$ $[M+H]^+$ 438.0482, found 438.0482.

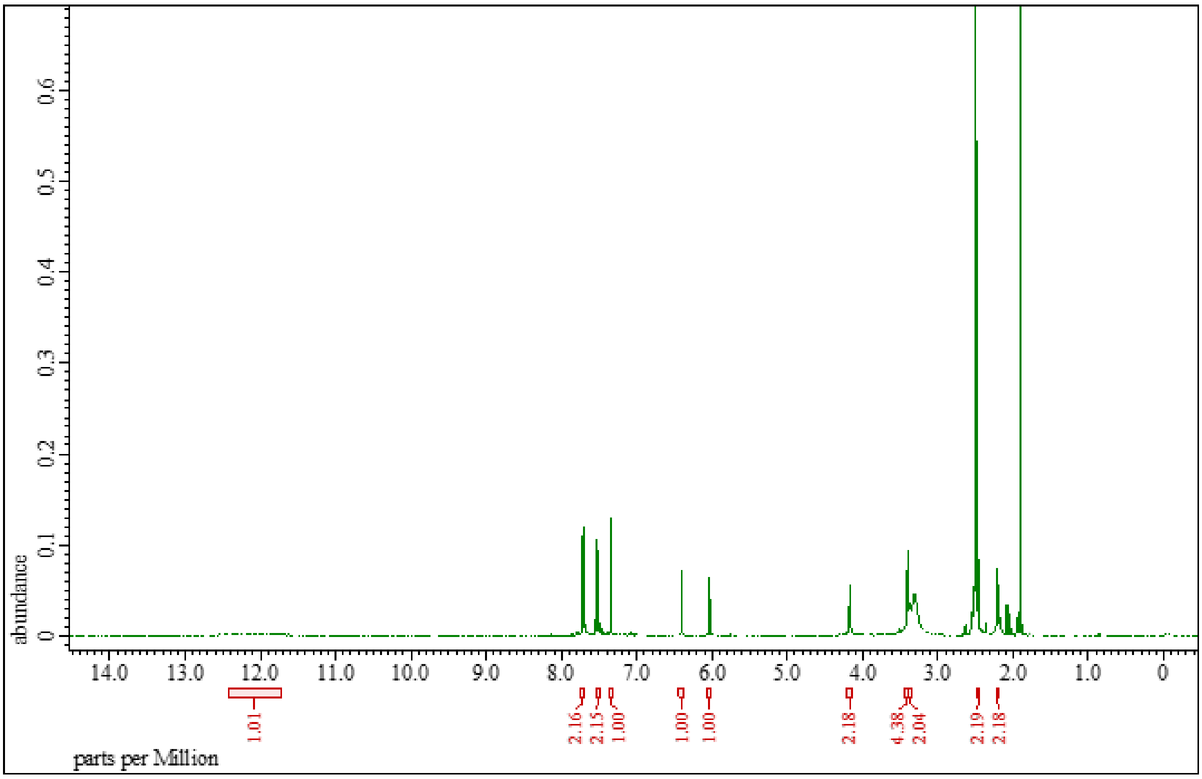

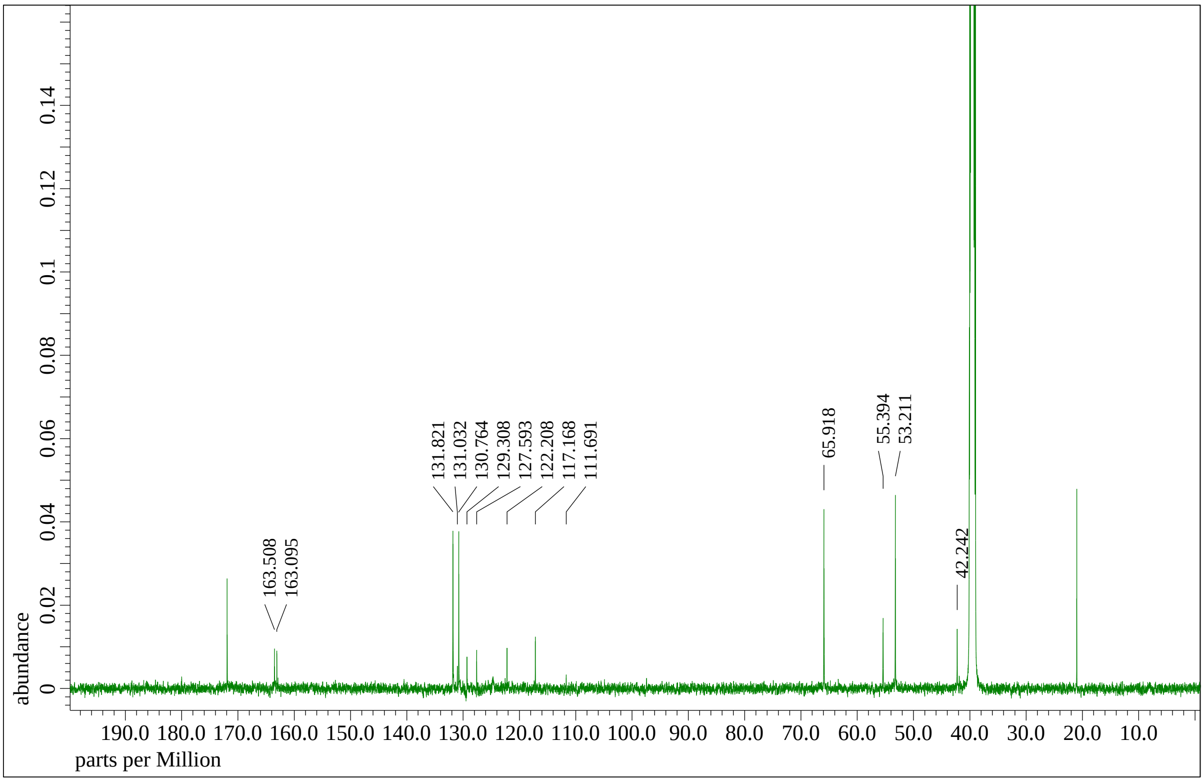

(3*R*)-7-benzyl-6-(thiophen-3-yl)-2,3-dihyhydroimidazo[2,1-*b*]thiazol-7-ium-3-carboxylate 8{*7,2*}

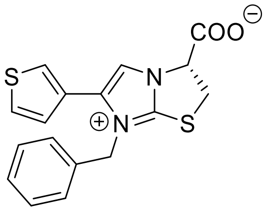

NMR: Mixture with 3% of **9**{*7,2*}. Creme amorphous solid, 22.9 mg (26%, 0.067 mmol). Cleaved from 465.3 mg of resin **2**{*7,2*} (0.556 mmol/g, 0.259 mmol of substrate). HPLC purity 98%. ^{1}H NMR (500 MHz, DMSO-d_6): δ = 8.09 (s, 1H), 7.90 (dd, *J* = 2.9, 1.4 Hz, 1H), 7.76 (dd, *J* = 5.0, 2.9 Hz, 1H), 7.37-7.38 (m, 3H), 7.31 (dd, *J* = 5.0, 1.4 Hz, 1H), 7.19 (dd, *J* = 7.6, 1.8 Hz, 2H), 5.68 (dd, *J* = 9.0, 3.9 Hz, 1H), 5.43 (d, *J* = 15.9 Hz, 1H), 5.38 (d, *J* = 15.9 Hz, 1H), 4.52 (dd, *J* = 11.7, 9.0 Hz, 1H), 4.25 (dd, *J* = 11.7, 3.9 Hz, 1H). ^{13}C NMR (126 MHz, DMSO-d_6): δ = 168.04, 151.49, 133.12, 132.71, 129.00, 128.88, 128.21, 128.03, 127.58, 127.41, 125.55, 118.77, 61.44, 50.81, 40.71. HRMS (ESI-TOF, pos.): *m/z* calcd for $C_{17}H_{15}N_2O_2S_2$ $[M+H]^+$ 343.0569, found 343.0571.

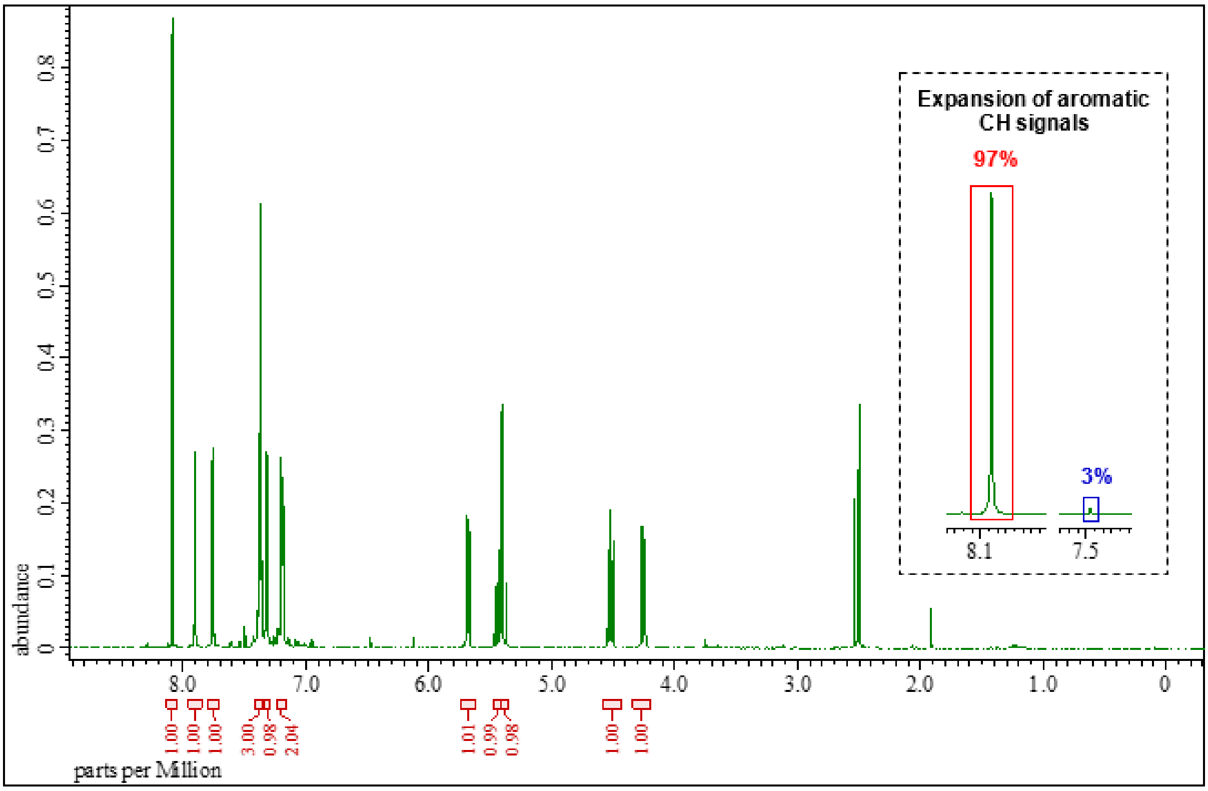
Expansion of aromatic CH signals
97%
3%
8.1
7.5
abundance
parts per Million
1.00
1.00
1.00
3.00
0.98
2.04
1.01
0.99
0.98
1.00
1.00

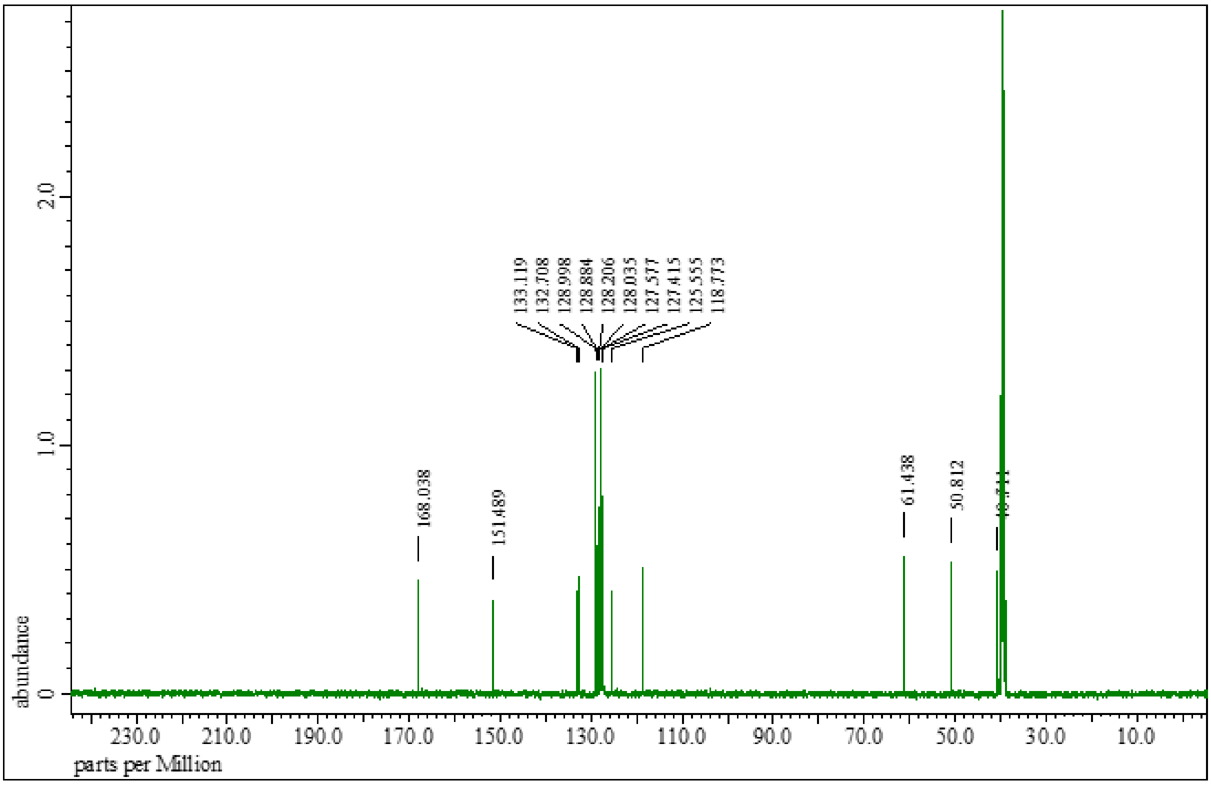
133.119
132.708
128.998
128.884
128.206
128.035
127.577
127.415
125.555
118.773
168.038
151.489
61.438
50.812
abundance
parts per Million

2-(3-benzyl-4-(thiophen-3-yl)-2-thioxo-2,3-dihydro-1*H*-imidazol-1-yl)acrylic acid 9{*7,2*}

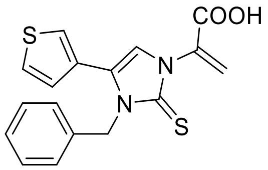

NMR purity 99%, yield quantitative (calculated from **8**{*7,2*}). ^{1}H NMR (500 MHz, DMSO-d_6): δ = 13.03 (br. s, 1H), 7.61 (dd, *J* = 5.0, 2.9 Hz, 1H), 7.54 (dd, *J* = 2.9, 1.3 Hz, 1H), 7.48 (s, 1H), 7.25-7.30 (m, 2H), 7.20-7.24 (m, 1H), 7.14 (dd, *J* = 5.0, 1.3 Hz, 1H), 7.05-7.08 (m, 2H), 6.46 (d, *J* = 0.7 Hz, 1H), 6.12 (d, *J* = 0.7 Hz, 1H), 5.46 (s, 2H). ^{13}C NMR (126 MHz, DMSO-d_6): δ = 164.27, 163.08, 136.85, 136.40, 128.40, 127.82, 127.27, 127.20, 127.17, 126.36, 125.78, 125.23, 124.11, 117.18, 48.06. HRMS (ESI-TOF, pos.): *m/z* calcd for $C_{17}H_{15}N_2O_2S_2$ [M+H]$^+$ 343.0569, found 343.0571.

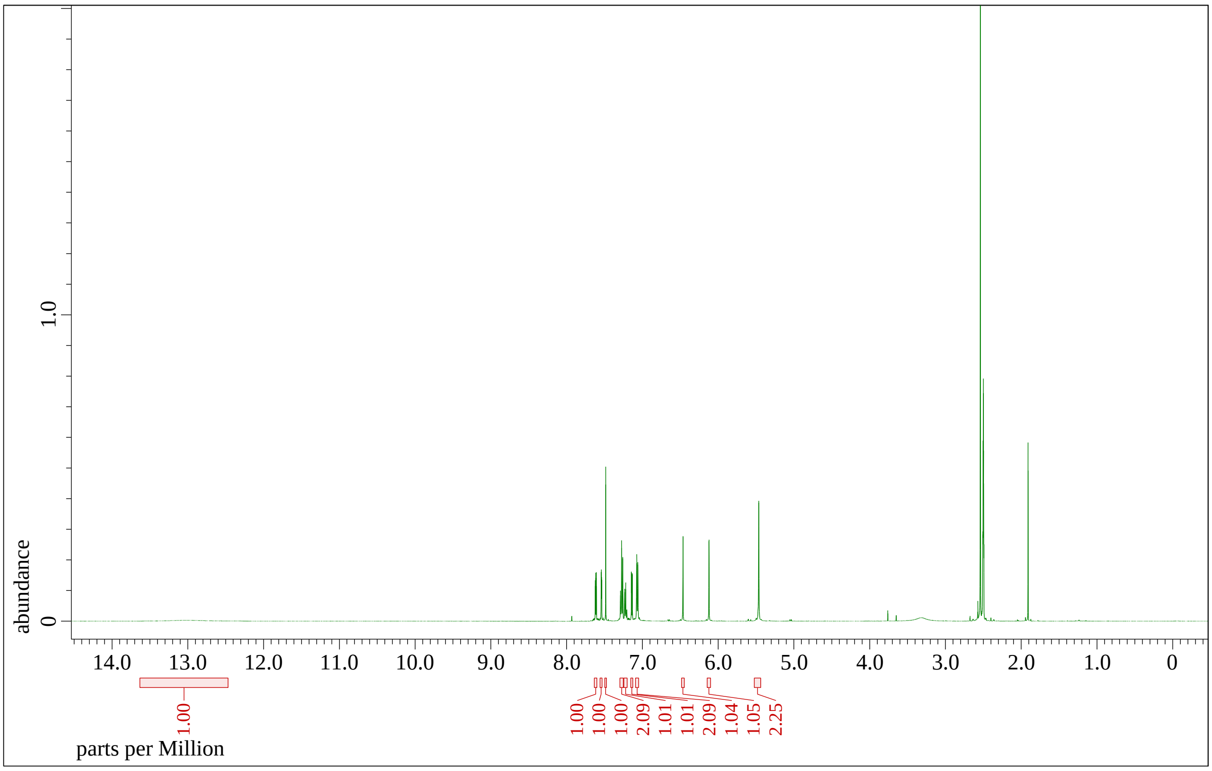

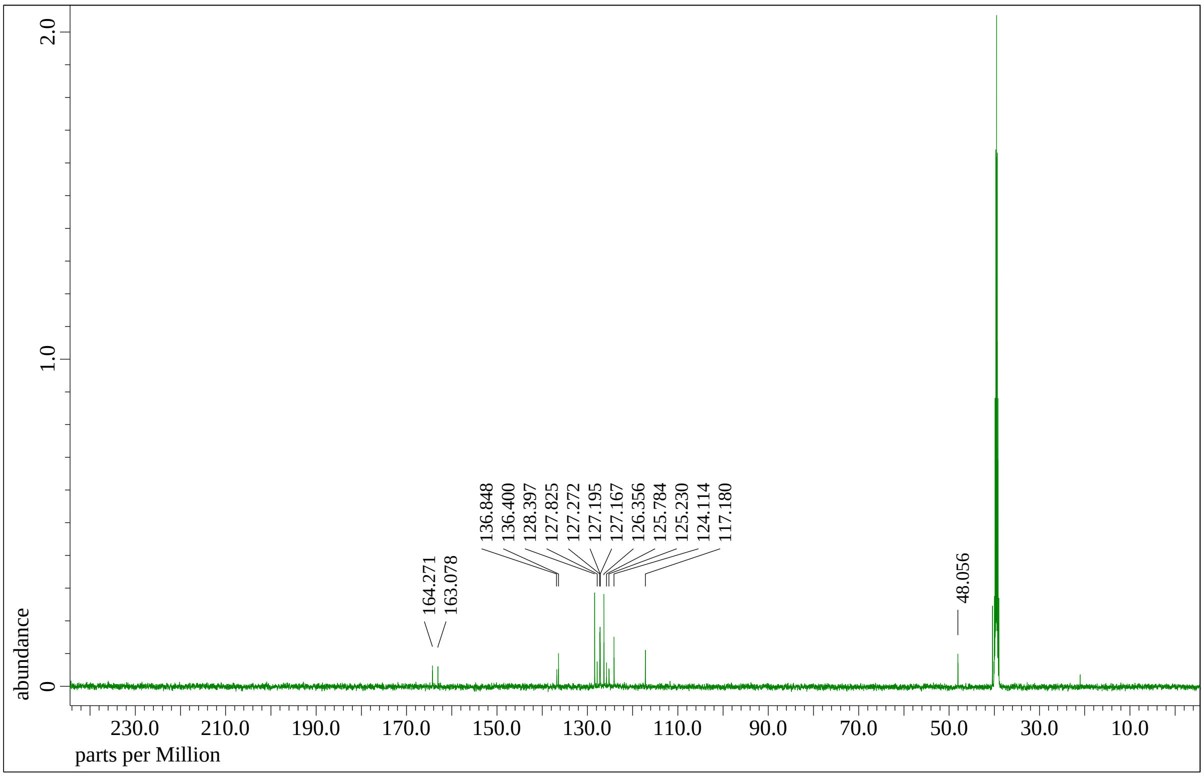

(3*R*)-7-allyl-6-(thiophen-3-yl)-2,3-dihyhydroimidazo[2,1-*b*]thiazol-7-ium-3-carboxylate 8{*7,3*}

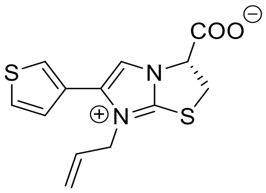

NMR: Mixture with 11% of **9**{*7,3*}. Creme amorphous solid, 16.9 mg (24.3%, 0.058 mmol) of which **8** 16.7 mg (24%, 0.057 mmol) and **9** 0.2 mg (0.3%, 0.0006 mmol). Cleaved from 433.5 mg of resin **2**{*7,3*} (0.556 mmol/g, 0.241 mmol of substrate). HPLC purity 98%. ^{1}H NMR (500 MHz, DMSO-d_6): δ = 7.97 (s, 1H), 7.86 (dd, *J* = 2.9, 1.4 Hz, 1H), 7.77 (dd, *J* = 5.0, 2.9 Hz, 1H), 7.34 (dd, *J* = 5.0, 1.4 Hz, 1H), 5.90-5.99 (m, 1H), 5.33 (br. d, *J* = 10.4 Hz, 1H), 5.13 (br. d, *J* = 17.2 Hz, 1H), 4.98 (dd, *J* = 8.5, 5.8 Hz, 1H), 4.70-4.74 (m, 2H), 4.34 (dd, *J* = 11.1, 8.5 Hz, 1H), 4.27 (dd, *J* = 11.1, 5.8 Hz, 1H). ^{13}C NMR (126 MHz, DMSO-d_6): δ = 165.66, 149.75, 131.98, 130.23, 127.98, 127.37, 126.17, 126.04, 119.41, 118.94, 64.06, 49.22, 41.62. HRMS (ESI-TOF, pos.): *m/z* calcd for $C_{13}H_{13}N_2O_2S_2$ $[M+H]^+$ 293.0413, found 293.0413.

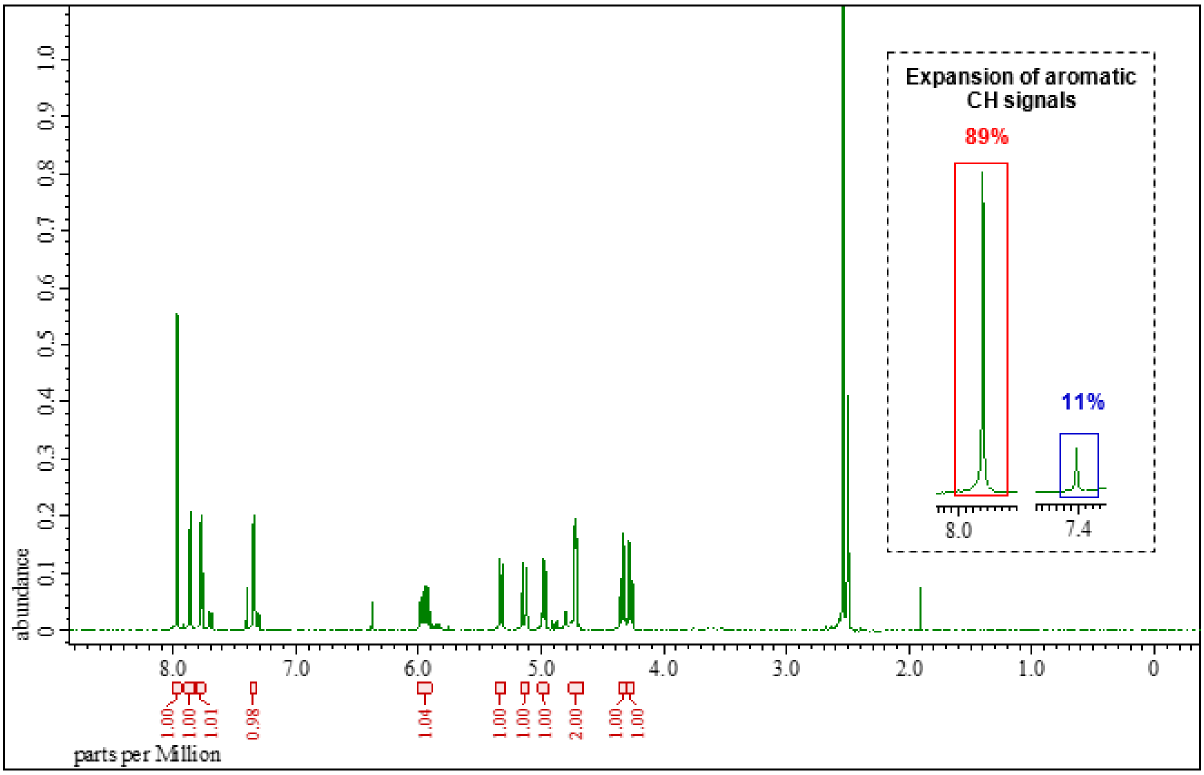
Expansion of aromatic CH signals
89%
11%
8.0
7.4
abundance
parts per Million
1.00
1.00
1.01
0.98
1.04
1.00
1.00
1.00
2.00
1.00
1.00

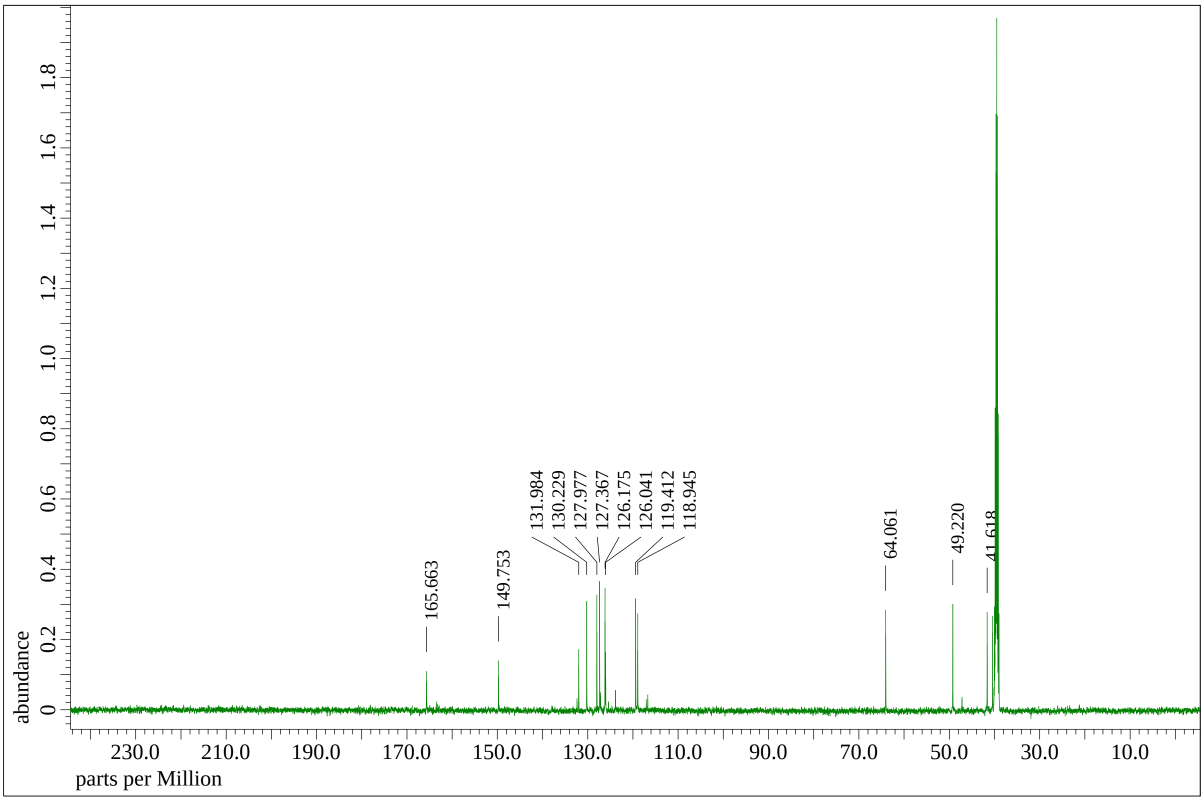
165.663
149.753
131.984
130.229
127.977
127.367
126.175
126.041
119.412
118.945
64.061
49.220
41.618
abundance
parts per Million

2-(3-allyl-4-(thiophen-3-yl)-2-thioxo-2,3-dihydro-1*H*-imidazol-1-yl)acrylic acid 9{*7,3*}

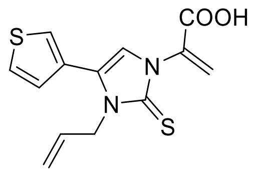

NMR purity 99%, yield quantitative (calculated from **8**{*7,3*}). ^{1}H NMR (500 MHz, DMSO-d_6): δ = 13.24 (br. s, 1H), 7.72 (dd, J = 2.9, 1.4 Hz, 1H), 7.69 (dd, J = 5.0, 2.9 Hz, 1H), 7.44 (s, 1H), 7.31 (dd, J = 5.0, 1.4 Hz, 1H), 6.44 (d, J = 0.7 Hz, 1H), 6.07 (d, J = 0.7 Hz, 1H), 5.82-5.90 (m, 1H), 5.13 (br. d, J = 10.5 Hz, 1H), 4.89 (br. d, J = 17.2 Hz, 1H), 4.79 (ddd, J = 4.7, 1.8, 1.8 Hz, 2H). ^{13}C NMR (126 MHz, DMSO-d_6): δ = 163.61, 163.07, 136.59, 132.23, 127.82, 127.39, 127.23, 125.81, 125.25, 124.09, 116.81, 116.74, 47.18. HRMS (ESI-TOF, pos.): m/z calcd for $C_{13}H_{13}N_2O_2S_2$ $[M+H]^+$ 293.0413, found 293.0413.

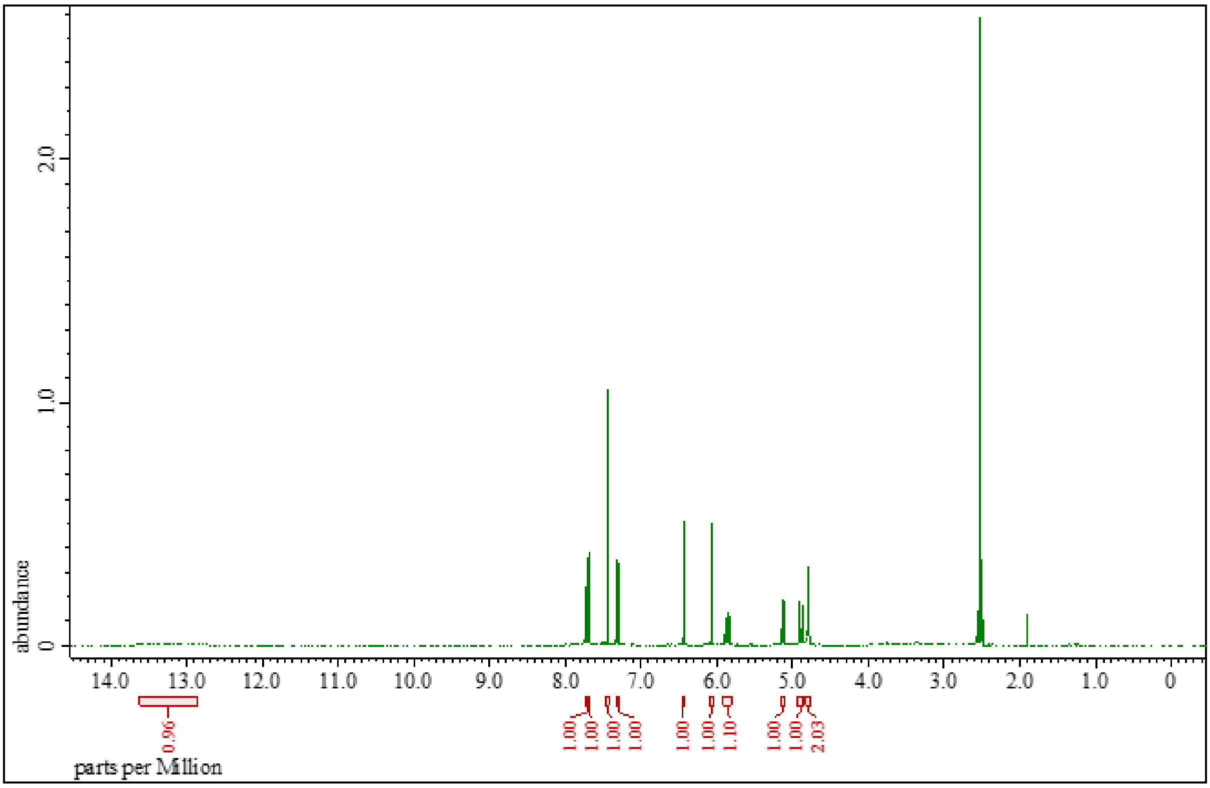

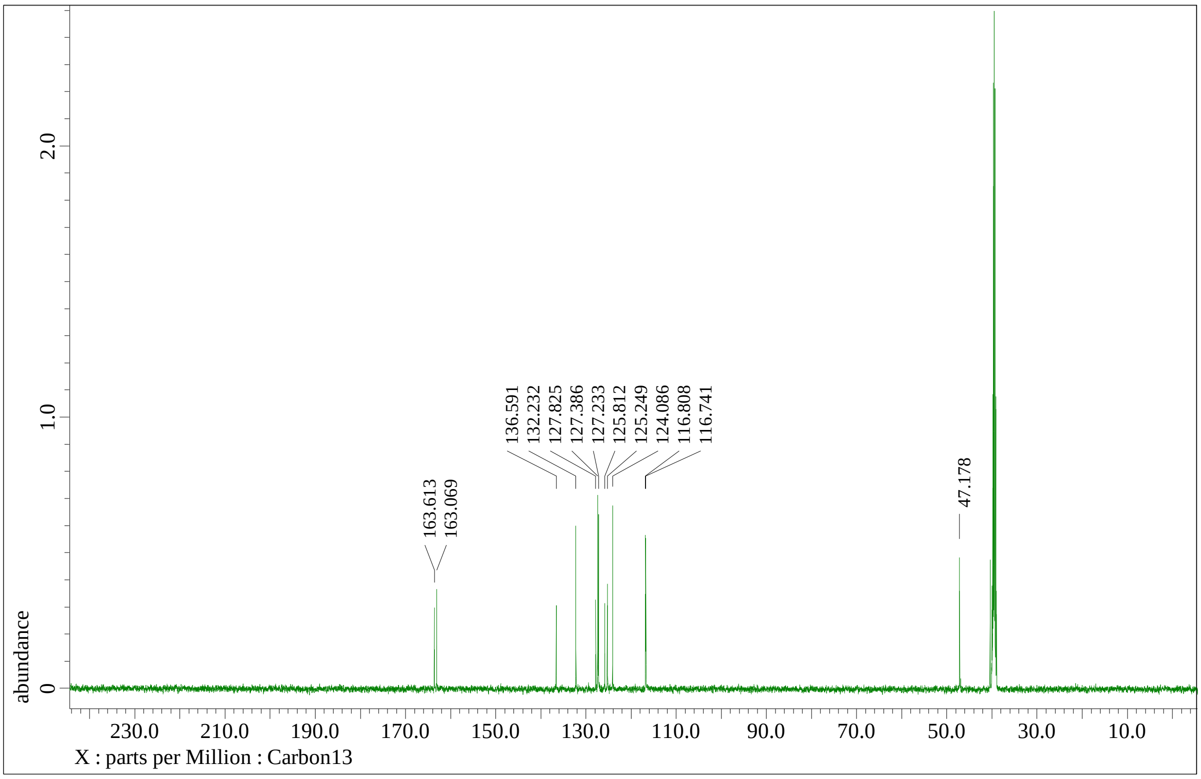

(3*R*)-7-(2-morpholinoethyl)-6-(thiophen-3-yl)-2,3-dihyhydroimidazo[2,1-*b*]thiazol-7-ium-3-carboxylate 8{*7,6*}

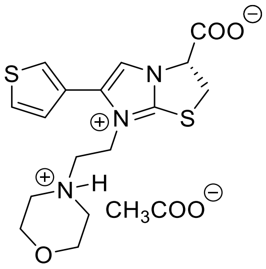

NMR: Mixture with 18% of **9**{*7,6*}. Creme amorphous solid, 27.3 mg (39%, 0.075 mmol) of which **8** 24.4 mg (33%, 0.067 mmol) and **9** 4.3 mg (6%, 0.012 mmol). Cleaved from 186.1 mg of resin **2**{*7,6*} (0.556 mmol/g, 0.103 mmol of substrate). HPLC purity 98%. ^{1}H NMR (500 MHz, DMSO-d_6): δ = 7.93 (dd, J = 2.8, 1.3 Hz, 1H), 7.89 (s, 1H), 7.78 (dd, J = 5.0, 2.8 Hz, 1H), 7.38 (dd, J = 5.0, 1.3 Hz, 1H), 5.03 (dd, J = 8.4, 5.9 Hz, 1H), 4.33 (dd, J = 11.1, 8.4 Hz, 1H), 4.24 (dd, J = 11.1, 5.9 Hz, 1H), 4.14 (t, J = 5.5 Hz, 2H), 3.52 (t, J = 4.5 Hz, 4H), 2.51-2.47 (m, 2H), 2.26-2.32 (m, 4H). ^{13}C NMR (126 MHz, DMSO-d_6): δ = 166.69, 150.35, 132.06, 128.10, 127.74, 126.73, 126.34, 118.90, 65.97, 64.00, 56.15, 53.24, 45.20, 41.33. HRMS (ESI-TOF, pos.): m/z calcd for $C_{16}H_{21}N_3O_3S_2$ $[M+H]^+$ 366.0941, found 366.0936.

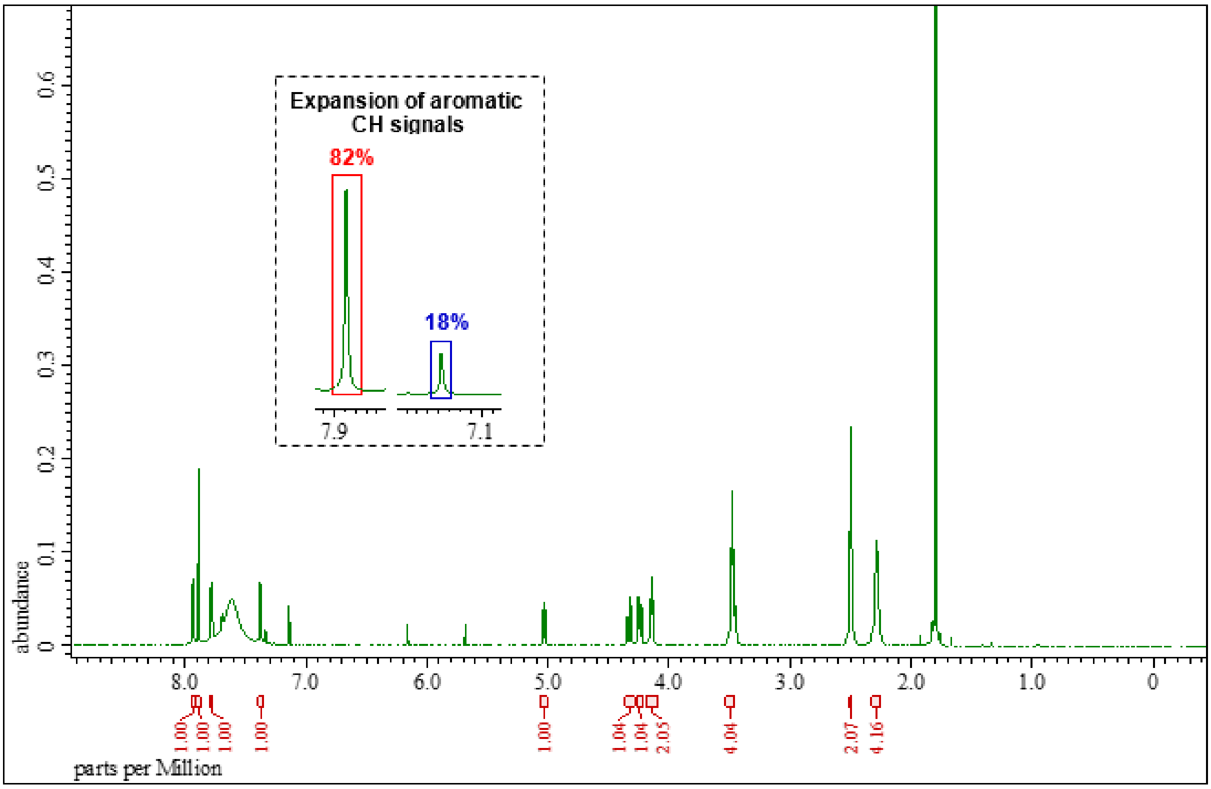
Expansion of aromatic CH signals
82%
18%
7.9
7.1
abundance
parts per Million
8.0
7.0
6.0
5.0
4.0
3.0
2.0
1.0
0
1.00
1.00
1.00
1.00
1.00
1.04
1.04
2.05
4.04
2.07
4.16

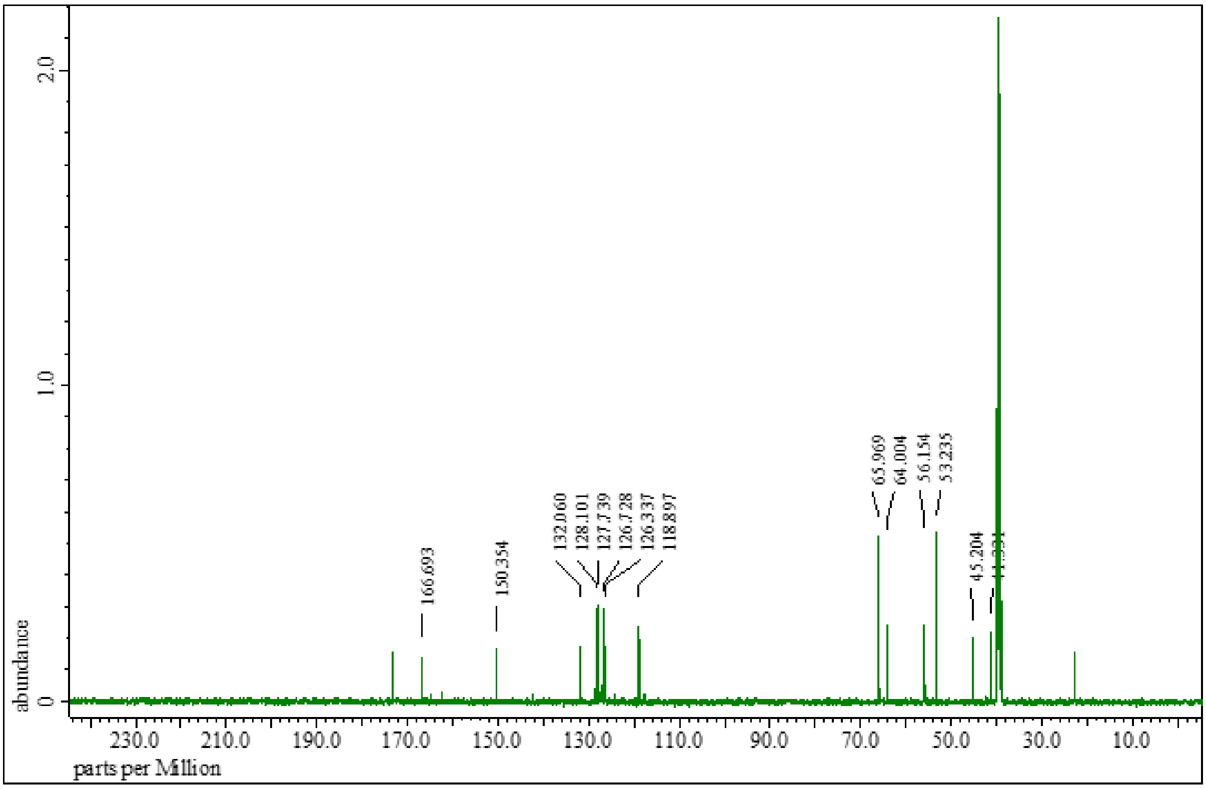
abundance
parts per Million
166.693
150.354
132.060
128.101
127.739
126.728
126.337
118.897
65.969
64.004
56.154
53.235
45.204
230.0
210.0
190.0
170.0
150.0
130.0
110.0
90.0
70.0
50.0
30.0
10.0
